# MS/MS Molecular Networking Unveils the Chemical Diversity of Biscembranoid Derivatives, Neutrophilic Inflammatory Mediators from the Cultured Soft Coral *Sarcophyton trocheliophorum*

**DOI:** 10.3390/ijms232415464

**Published:** 2022-12-07

**Authors:** Ngoc Bao An Nguyen, Lo-Yun Chen, Po-Jen Chen, Mohamed El-Shazly, Tsong-Long Hwang, Jui-Hsin Su, Chun-Han Su, Pei-Tzu Yen, Bo-Rong Peng, Kuei-Hung Lai

**Affiliations:** 1 Graduate Institute of Pharmacognosy, College of Pharmacy, Taipei Medical University, Taipei 11031, Taiwan; 2 Department of Medical Research, E-Da Hospital, Kaohsiung 824005, Taiwan; 3 Department of Pharmacognosy, Faculty of Pharmacy, Ain-Shams University, Organization of African Unity Street, Abassia, Cairo 11566, Egypt; 4 Research Center for Chinese Herbal Medicine, College of Human Ecology, Chang Gung University of Science and Technology, Taoyuan 33303, Taiwan; 5 Graduate Institute of Health Industry Technology, College of Human Ecology, Chang Gung University of Science and Technology, Taoyuan 33303, Taiwan; 6 Graduate Institute of Natural Products, College of Medicine, Chang Gung University, Taoyuan 33302, Taiwan; 7 Department of Chemical Engineering, Ming Chi University of Technology, New Taipei City 24301, Taiwan; 8 Department of Anesthesiology, Chang Gung Memorial Hospital, Taoyuan 33305, Taiwan; 9 National Museum of Marine Biology and Aquarium, Pingtung 94450, Taiwan; 10 Department of Marine Biotechnology and Resources, National Sun Yat-sen University, Kaohsiung 80424, Taiwan; 11 Department of Food Science, College of Human Ecology, Fu Jen Catholic University, New Taipei City 24205, Taiwan; 12 Jian Sheng Tang Chinese Medicine Clinic, Kaohsiung 80664, Taiwan; 13 Crystal Clear Kampo Clinic, Tainan 70156, Taiwan; 14 PhD Program in Clinical Drug Development of Herbal Medicine, College of Pharmacy, Taipei Medical University, Taipei 11031, Taiwan; 15 Traditional Herbal Medicine Research Center, Taipei Medical University Hospital, Taipei 11031, Taiwan

**Keywords:** *Sarcophyton trocheliophorum*, biscembranoids, MS/MS molecular networking, anti-inflammation, antioxidant, natural products

## Abstract

Biscembranoids are the distinctive tetraterpenoids owing a 14/6/14 membered tricyclic scaffold that have been mainly discovered in the soft corals, especially the genera *Sarcophyton*, *Lobophytum* and *Sinularia*. Recent findings have demonstrated the great anti-inflammatory potential of biscembranoid analogues in human neutrophils, motivating more chemical and biological explorations targeting these marine-derived natural products. In the current study, the chemical diversity of biscembranoids derived from the cultured-type *Sarcophyton trocheliophorum* von Marenzeller was illustrated through MS/MS molecular networking (MN) profiling approach. Based on the MN patterns, the prioritization of unknown biscembranoid derivatives was putatively analyzed. As a result, the biscembrane targeting isolation afforded two new metabolites, sarcotrochelides A (**1**) and B (**2**), along with six known analogues (**3**–**8**). Their structures and relative configurations were determined by spectroscopic methods. In vitro neutrophil inflammatory inhibition was further investigated for all isolates based on reduced superoxide anion (O_2_^•−^) generation detections. Compounds **5**–**8** showed significant dose-dependently inhibitory effects, suggesting the cruciality of 6,7-dihydrooxepin-2(5H)-one moiety and saturated γ-lactone ring in their reactive oxygen species (ROS)-dependent anti-inflammatory properties.

## 1. Introduction

Biscembranoids are the distinctive tetraterpenoids owing a 14/6/14 membered tricyclic scaffold that have been mainly discovered in the marine organisms, especially the soft corals belonging to the genus *Sarcophyton* [1,2,3,4,5,6,7,8,9,10,11,12,13,14,15,16,17,18,19,20,21,22,23,24,25,26,27,28]. These secondary metabolites was first found in 1986 [1] and exhibited various bioactivities, ranging from anti-cancer, anti-inflammatory, neuroprotective, anti-microbial, and immunomodulatory activities. The anti-inflammatory effect accounts for the majority of bioactivities of the tetraterpene derivatives. Recent studies have also shown the great anti-inflammatory potential of biscembranoid analogues in human neutrophils, which has attracted more chemical and biological explorations targeting these marine-derived natural products.

Due to the promising pharmacological actions, higher quantity of potent marine-derived compounds is required for subsequent preclinical and clinical trials, but the yields of the secondary metabolites obtained from the wild-type marine organism are usually insufficient for these purposes. Therefore, marine aquaculture has emerged as an effective approach to maintain a sustainable, consistent, and reproducible supply of marine-derived natural products.

**Figure 1:**
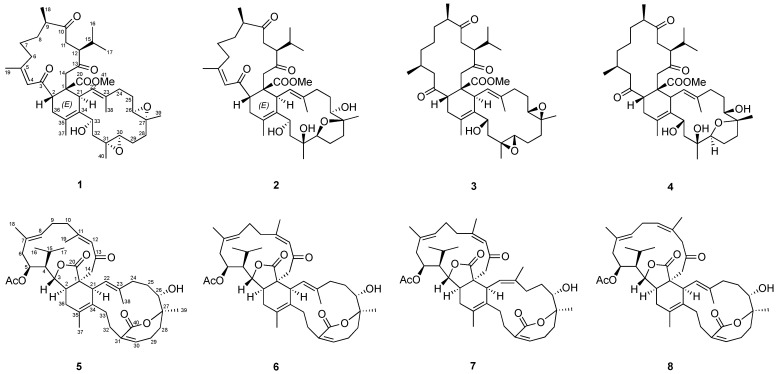
The identified biscembranoids from the cultured soft coral *Sarcophyton trocheliophorum*.

In the current study, a MS/MS molecular networking method has been utilized to assist in the discovery of new biscembranoids from the cultured soft coral *Sarcophyton trocheliophorum*. Based on the MN patterns and NMR spectra, the fraction that was putatively identified to contain biscembranoids was subjected to further purification steps. The biscembrane targeting isolation afforded two new metabolites, sarcotrochelide A (**1**) and B (**2**), along with six known analogues (**3**–**8**) (Figure 1). Their structures were determined by spectroscopic methods. In vitro neutrophil inflammatory inhibition was further investigated for all isolates based on ROS generation detections using luminol enhanced chemiluminescence. Compounds **5**–**8** showed significant dose-dependently inhibitory effects, suggesting the cruciality of 6,7-dihydrooxepin-2(5*H*)-one moiety in their anti-inflammatory properties.

## 2. Results and Discussion

### 2.1. Characterizing the Distribution of Anti-Inflammatory Biscembranoids Using Multi-informative Molecular Networking (MIMN)

In order to facilitate the operating process for probing anti-inflammatory biscembranoids, a multi-informative molecular networking (MIMN) was applied [29]. In the primary extraction and fractionation part, the organic extract (using dichloromethane:methanol = 1:1) of the cultured-type *Sarcophyton trocheliophorum* was fractionationed into the EtOAc and the aqueous layers through liquid-liquid partitioning approaches. The chromatographic separation (normal phase) on EtOAc soluble residue further afforded 20 subfractions. Then the chemical and anti-inflammatory MIMN profiles of these fractions were constructed based on the MS/MS analysis and superoxide anion (O_2_^•−^) inhibitory assessments in activated neutrophils (Table 1), respectively. The followed-up clustering, classification, and annotation were performed on the GNPS platform (https://gnps.ucsd.edu, accessed on 17 April 2022).

**Figure 2:**
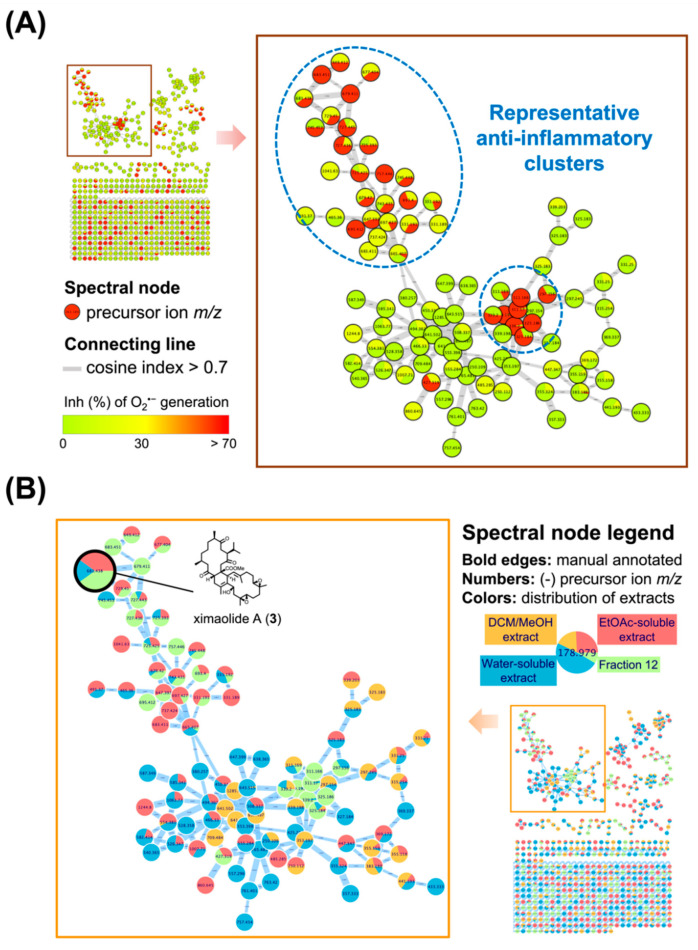
The multi-informative molecular networking (MIMN) profiles illustrated the relationships between the metabolomic diversity and anti-inflammatory property of the *Sarcophyton trocheliophorum* extract. (**A**) The established MIMN is based on the inhibition of O_2_^•−^ generation (10 μg/mL) in *N*-formylmethionyl-leucyl-phenylalanine (fMLF)-induced neutrophils and the spectral nodes were colored according to the levels of inhibition; (**B**) the MIMN spectral nodes are labeled according to the distributions of each extract and fractions.

Based on the MIMN analyzing results, the clusters of cembrane dimer (*m*/*z* 650–750) and monomer (*m*/*z* 290–350) exhibited the greatest anti-inflammatory potential with inhibition rate over 70% at the concentration of 10 μg/mL (Figure 2A). The insight MIMN patterns of metabolite distribution (Figure 2B) revealed that the fraction 12 from the EtOAc-soluble extract contained a variety of anti-inflammatory biscembranoids, resulting the further isolation targeting these characteristic tetraterpenoids from fraction 12.

### 2.2. Chemical Identification of Isolated Compounds

The target isolation of Fraction 12 of soft coral *S*. *trocheliophorum* yielded eight compounds (**1**–**8**), including six known ones (**3**–**8**) identified as ximaolide A (**3**) [9], methyl tortuoate D (**4**) [18], glaucumolide A (**5**) [20], glaucumolide B (**6**) [20], bistrochelide A (**7**) [18], and bistrochelide B (**8**) [18] by comparing their NMR spectroscopic data with those reported in the literature.

Sarcotrochelide A (**1**) was isolated as a white powder. The positive mode high resolution electrospray ionization mass spectrum (HRESIMS) showed a peak at *m*/*z* 681.4362, suggesting a molecular formula of C_41_H_60_O_8_ (calcd. for [C_41_H_60_O_8_ + H]^+^, 681.4366), and implying 12 degrees of unsaturation. The signal at 3417 cm^−1^ in the IR spectrum indicated the presence of the hydroxy group. A total of 41 carbons in the structure of compound **1** were deduced from the ^13^C NMR spectrum. The multiplicity of carbon signals was determined from distortionless enhancement by polarization transfer (DEPT) and heteronuclear single quantum coherence (HSQC) spectra, including nine methyls (including one methoxyl), eleven methylenes, ten methines, and eleven non-protonated carbons. The ^1^H and ^13^C NMR spectra signals (Table 2) showed three olefinic methyl groups [*δ*_H_ 1.89 (s); 1.74 (s); 1.62 (s); *δ*_C_ 26.3, 18.7, 17.3], two methyls attached to oxygen-bearing quaternary carbon [*δ*_H_ 1.25 (s); 1.25 (s); *δ*_C_ 16.3, 18.7], two methyls of an isopropyl group [*δ*_H_ 0.74 (d, *J* = 6.8 Hz); 0.95 (d, *J* = 6.8 Hz); *δ*_C_ 18.4, 21.2], and one methoxy group [*δ*_H_ 3.50, s; *δ*_C_ 51.3], two trisubstituted double bond [*δ*_H_ 6.59 (brs); 5.10 (d, *J* = 11.1 Hz); *δ*_C_ 126.7, CH; 126.7, CH; 134.0, C; 161.1, C]; one tetrasubstituted double bond (*δ*_C_ 130.4, C; 131.2, C); three oxygen-bearing methines [*δ*_H_ 2.29 (dd, *J* = 8.8, 4.1 Hz); 2.91 (dd, *J* = 6.1, 4.3 Hz); 4.79, (dd, *J* = 10.7, 2.1 Hz); *δ*_C_ 60.8, CH; 61.5, CH; 65.1, CH]; two oxygenated quaternary carbon (*δ*_C_ 59.4, 60.0), and four carbonyl carbons (*δ*_C_ 174.6, 203.3, 209.4, 214.8). The spectral analysis suggested the possible presence of a biscembranoid framework.

**Figure 3:**
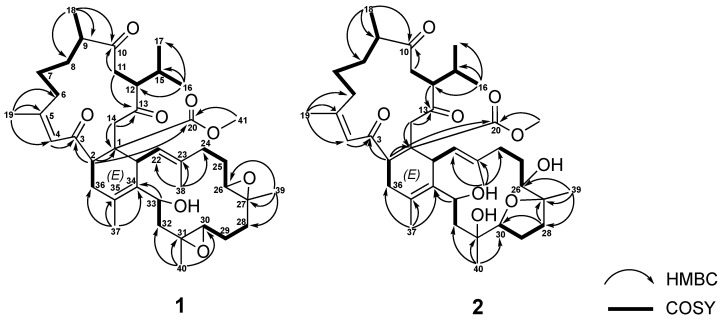
The selected ^1^H-^1^H COSY and HMBC correlations of **1** and **2**.

The correlations spectroscopy (COSY) spectrum (Figure 3) of **1** was applied to identify seven different spin systems from H-2 to H_2_-36; H_2_-6 via H_2_-7, H_2_-8, H-9, and H_3_-18; isopropyl protons H_3_-16 and H_3_-17 via H-15, H-12, and H-11; H-21 to H-22; H_2_-24 via H_2_-25 and H_2_-26; H_2_-28 via H_2_-29 and H-30; and H_2_-32 to H-33. These units were assembled by heteronuclear multiple bond correlation (HMBC) (Figure 3) of H_3_-16 to C-12, C-15, and C-17; H_3_-18 to C-8, C-9, and C-10; H_3_-19 to C-4, C-5, and C-6; H_3_-37 to C-34, C-35, and C-36; H_3_-38 to C-22, C-23, and C-24; H_3_-39 to C-26, C-27, and C-28; H_3_-40 to C-30, C-31, and C-32; H-2 to C-1, C-3, C-4, and C-20; H_2_-11 to C-10 and C-13; H_2_-14 to C-1; and H-33 to C-34. The above-mentioned group accounted for ten of the total twelve degrees of unsaturation, implying the presences of two additional rings. These were suggested to be two trisubstituted epoxide groups with methyl singlets at δ_H_ 1.24 (3H, s, H_3_-39) and 1.25 (3H, s, H_3_-40), epoxymethine multiplets at δ_H_ 2.91 (H-26) and 2.29 (H-30), and ^13^C NMR signals at 61.5 (C-26), 59.4 (C-27), 60.8 (C-30), and 60.0 (C-31). The gross structure of **1** was thus confirmed as shown in Figure 1, which possesses a biscembranoid skeleton similar to ximaolide A (**3**) [9].

**Figure 4:**
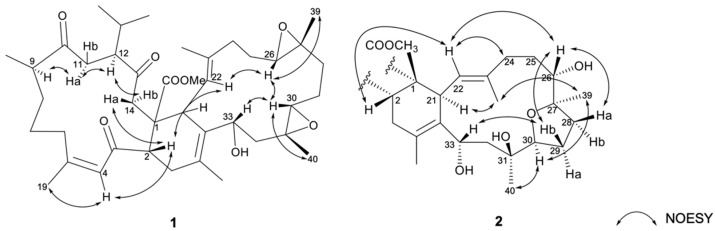
The selected NOESY correlations of **1** and **2**.

The relative stereochemistry of **1** was elucidated by correlations in the nuclear Overhauser effect relationships (NOESY) experiment. As shown in Figure 4, the NOESY correlations of H-4 (*δ*_H_ 6.59, br s) with H_3_-19 (*δ*_H_ 1.89, s), together with the obviously downfield-shifted methyl at C-19 (*δ*_C_ 26.3, CH_3_), suggested a *cis* geometry of C-4/C-5 trisubstituted double bond. Assuming the *β*-orientations of H-2 and H_3_-41 as previously reported, one of the methylene protons at C-14 (*δ*_H_ 3.05, d, *J* = 18.6 Hz) exhibited NOE correlations with H-2 and was assigned as H-14β, while the other (*δ*_H_ 2.71–2.77, m) was denoted as H-14α. The NOESY correlations observed between H-9 with H-11α (*δ*_H_ 2.71–2.80, m), H-11α (*δ*_H_ 2.71–2.80, m) with H-12 and H-14α, reflected the α-orientations of H-9 and H-12. Furthermore, the correlations of H-2 with H-22; H-22 with H-26; H-26 with H-30 and H_3_-39; H-30 with H-33 and H_3_-40, determined the *β*-orientation of the H-26, H-30, H-33, H_3_-39, and H_3_-40. Moreover, the ^13^C NMR signal of C-38 (*δ*_C_ 17.3, CH_3_) indicated the *E* geometry of the trisubstituted C-22/C-23 double bond. On the basis of the above observations and as the relative configurations of **1** determined as shown, the structure of compound **1** could be fully established as 1S*, 2S*, 9R*, 12S*, 21S*, 26R*, 27R*, 30R*, 31R*, 33R*.

Sarcotrochelide B (**2**) was isolated as a white powder. Its formula was determined as C_41_H_62_O_9_ by the HRESIMS ion at *m*/*z* 699.4467 (calcd. for [C_41_H_62_O_9_ + H]^+^, 699.4472), indicating 11 indices of hydrogen deficiency. The hydroxy-containing structure of **2** was inferred from the IR signal at 3417 cm^–1^. 41 carbons, including 9 methyls, 11 methylenes, 10 methines, and 11 quaternary carbons, were revealed by the ^13^C NMR spectrum. The NMR signals (Table 3) showed three olefinic methyl groups (δ_H_ 1.89, s; 1.61, s; 1.72, s and δ_C_ 26.4; 18.1; 16.4, respectively), two methyl groups linked to oxygen-bearing quaternary carbons (δ_H_ 1.17, s; 1.19, s and δ_C_ 20.6; 21.4, respectively), one methoxy group (δ_H_ 3.50, s; δ_C_ 51.1), two methyls of an isopropyl group (δ_H_ 2.32–2.38, m; 0.74, d, *J* = 6.8 Hz; 0.98, d, *J* = 6.8 Hz; δc 28.6, CH;18.2, CH_3_; 21.4, CH_3_), two trisubstituted double bonds (δ_H_ 6.59, s; δc 126.4, CH; 161.4, C and δ_H_ 4.99, d, *J*= 10.8 Hz; δc 128.0, CH; 137.2, C, respectively), one tetrasubstituted double bond ( δc 125.3, C and 132.2, C), three oxymethines (δ_H_ 3.27, m; 3.96, dd, *J*= 10.3, 6.3 Hz; 5.05, d, *J*= 11.2 Hz and δc 74.1, CH; 88.4, CH; 67.5, CH, respectively), two oxygen-bearing quaternary carbons (δc 86.0, C and 76.3, C), and four carbonyl carbons (δc 202.9, C; 214.7, C; 208.7, C and 174.9,C). These findings established the biscembranoid scaffold of **2**. The COSY signals (Figure 3) revealed seven different spin systems from H-2 to H_2_-36; H_2_-6 via H_2_-7, H_2_-8, H-9 and H_3_-18; H_2_-11 via H-12, H-15, H_3_-16 and H_3_-17; H-21 to H-22; H-24 via H_2_-25 and H-26; H_2_-28 via H_2_-29 and H-30; and H_2_-32 to H-33. These units were assembled by heteronuclear multiple bond correlation (HMBC) of H_3_-16 to C-12, C-15 and C-17; H_3_-18 to C-8, C-9 and C-10; H_3_-19 to C-4, C-5 and C-6; H_3_-37 to C-34, C-35 and C-36; H_3_-38 to C-22, C-23 and C-24; H_3_-39 to C-26, C-27 and C-28; H_3_-40 to C30, C-31 and C-32; H-2 to C-1, C-3, C-14 and C-20; H_2_-11 to C-10; H-12 to C-13; H_2_-14 to C-1, C-13 and C-20; and H-33 to C-34. The above-mentioned biscembrane framework put paid to ten of the total eleven unsaturated degrees, implying the existence of an additional ring. It was suggested to be of an ester ring between C-27 and C-30 established via an HMBC from H_2_-28 to C-27 and C-30. The gross structure of **2** was thereby confirmed, as shown in Figure 2, which possesses a biscembranoid skeleton similar to methyl tortuoate D (**4**) [18].

The relative configurations of rings A and B of compound **2** were identical to those of co-occurring compound **1** as determined by the similar NOESY and the NMR data. NOESY correlations (Figure 4) of H_3_-40/H-30, H-30/H_3_-39, H_3_-39/H_3_-38, and H_3_-38/H-21 were observed, suggesting that H-21, H-30, H_3_-39, and H_3_-40 were co-facial and were assigned as α-orientations. In consequence, NOE correlations of H-2/H-22, H-22/H_2_-24, H-22/H-26, H-26/H-28a (*δ*_H_ 2.34–2.40, m), H-26/H-29b (*δ*_H_ 1.54–1.60, m), H-29b/H-33 suggested that H-26 and H-33 was β-oriented. Therefore, the structure of **1** was unambiguously elucidated as shown in Figure 2. On the basis of the above observations and as the relative configurations of **2** have been determined as shown, the structure of compound **2** could be fully established as 1S*, 2S*, 9R*, 12S*, 21S*, 26R*, 27R*, 30R*, 31S*, 33R*.

### 2.3. Bioactivities of the Biscembranoids

The activation effects of *N*-formyl-methionyl-leucyl-phenylalanine (fMLF) and pathogen-associated molecular patterns (PAMPs) on neutrophils can cause a series of inflammatory responses, such as respiratory burst (O2^•−^ generation) and degranulation (elastase release) [30]. In order to evaluate the anti-inflammatory activities of the cultured soft coral *S. trocheliophorum*, the EtOAc, MeOH, and water-soluble extracts, and fraction 12 were assayed in fMLF-induced human neutrophils. All eight pure compounds obtained from fraction 12 were also evaluated for their anti-inflammatory effects in the same in vitro tests. The results showed that fraction 12 exhibited the highest inhibitory effects on superoxide anion generation and elastase release with IC_50_ 5.45 and 7.48 μg/mL, respectively, among the crude samples (Table 1).

For pure derivatives, compound **6** displayed the strongest activity against superoxide anion generation and elastase release in fMLF/CB-induced human neutrophils, followed by compounds **5**, **7**, and **8**, respectively (Table 4).

The significant difference in the anti-inflammatory effects of the isolates may be caused by some variations in their structures. The common characteristics of the four most active compounds is that they share a 6,7-dihydrooxepin-2(5H)-one moiety and a saturated γ-lactone ring. In addition, compound **6** possesses a 11*Z* and 22*E* double bonds instead of an *E* geometry of Δ^11(12)^ and Δ^22(23)^ in compound **5** and a *Z* geometry of Δ^11(12)^ and Δ^22(23)^ in compound **7**. Additionally, when compared to compound **6**, the 11,12-double bond was replaced by a 10,11-double bond in compound **8**, which suggested that the reduced anti-inflammatory effect of compound **8** might be caused by this minor change.

## 3. Materials and Methods

### 3.1. General Experimental Procedures

The optical rotation was measured by a polarimeter JASCO P-2000 (JASCO, Tokyo, Japan). The infrared spectra were obtained on a FT-IR spectrophotometer, Nicolet™ iS™ 5 FTIR Spectrometer (Thermo Fisher Scientific, Waltham, MA, USA). UV spectra were collected by Spectrophotometer U-3310 UV-Vis (Hitachi, Ltd., Tokyo, Japan). The 1D and 2D NMR spectra were obtained on an Agilent 600 MHz DD2 NMR (Agilent, Santa Clara, CA, USA). The chloroform-d was used as the internal lock. HRESIMS data in positive mode were collected on a Waters LC/Q-TOF SYNAPT G2 (Waters Corporation, Milford, MA, USA) system. All isolations were purified by MPLC and HPLC. The former is Biotage^®^ Isolera™ Systems (Biotage, Uppsala, Sweden), and the powder was filled in the flash column, Biotage^®^ SNAP Cartridge KP-Sil 10 g (Biotage, Uppsala, Sweden), the latter is HPLC system Shimazu LC-2050 (Shimazu, Kyoto, Japan) with a Galaksil column EF-C18-H (5 μm, 120 Å, 10 × 250 mm, C18; Galak Chromatography, Wuxi, Jiangsu, China).

### 3.2. Non-Targeted Fragment Ions Collection Using Ultra-Performance Liquid Chromatography-Tandem Mass Spectrometry (UPLC-MS/MS)

The acquisition of tandem mass spectral data was carried out based on a Waters SYNAPT G2 LC/Q-TOF (Waters Corporation, Milford, MA, USA) system. The extracts were filtered through a 0.45 μm membrane filter and dissolved in methanol to a final concentration of 5000 ppm for the analysis. For chromatographic part, a C18 column of Waters Acquity UPLC BEH (Waters, 1.7 µm, 2.1 mm × 100 mm) was used for the separation and was maintained in a 40 °C column oven. The analytes were eluated from the column by CH_3_CN (A, containing 0.1% formic acid)/water (W, containing 0.1% formic acid) gradient sequences: 0.01–25 min, 1–100% A; 25.01–30 min, 100% A, with the flow rate of 0.5 mL/min. Automatical injections were executed with injected volumes of 5 μL. The non-targeted MS^1^ and MS^2^ data were acquired within the range of *m*/*z* 100–2000. The automated data-dependent acquisition (DDA) approach was used for the acquisition of MS^2^ spectra, and five precursor ions were selected for further fragmentations with ramping of the collision energy from 10–50 eV. Finally, the finalization of MS data were conducted with the assistance of Waters MassFragment software (MassLynx4.1, Waters, MA, USA).

### 3.3. GNPS-Based Molecular Networking Analysis

A GNPS web-based platform (https://gnps.ucsd.edu, accessed on 17 April 2022) [31] was applied to analyze and output the MS/MS molecular networking data (job ID: edcb54569b0042fea7104782189e34af, 17 April 2022). The pre-processing of MS/MS raw data was conducted by converting into mzML file using Proteowizard MSConvert (Ver. 3, GitHub repository, Palo Alto, CA, USA). The conversions were uploaded to GNPS drive using WinSCP software (Ver. 5.21, SourceForge, San Diego, CA, USA) and performed molecule networking analysis. The MS/MS spectra were window-filtered according to the top five strongest ion peaks in the ±50 Da window throughout the spectrum. A molecular network was then created, in which the edges between nodes were kept if the cosine scores were above 0.70 and the separated consensus spectra shared at least four matched peaks. Then the appeared nodes in the network were annotated based on the experimental MS^2^ fragmentations of isolates. The data visualization was executed by Cytoscape 3.8.2 (Cytoscape 3.8.2, NRNB, San Diego, CA, USA) [32].

### 3.4. Animal Material, Extraction, and Isolation

Specimens of the wild-type *S. trocheliophorum* was originally collected by scuba diving from the coast of Pingtung, Taiwan, in 2015 (specimen No. 2015-07-ST). These corals were preserved and aquacultured in National Museum of Marine Biology and Aquarium (Pingtung, Taiwan). The aquaculture condition was mentioned below [33]: The collected wild corals were cut into several sub-strains of 4 to 5 cm, and these sub-strains are naturally placed and attached to porous tiles for domestication and cultivation. These soft corals were kept in cultured tanks (120 tons) with temperature controlled (25–28 °C) coolers and supported by natural light daily. The ecological environment was settled up with live sea rocks, live sea sands, snails, paracanthurus hepatus fishes, sea urchins, sea cucumbers, and other aquaculture soft corals, such as *Briareum* spp., *Paralemnalia* sp., *Sarcophyton* spp., and *Sinularia* spp. The specimens were then collected by hand in July 2020 and were kept in a −20 °C freezer until extraction. A voucher specimen (specimen no. 202007ST1) was deposited in the Graduate Institute of Pharmacognosy, Taipei Medical University.

The cultured-type *S. trocheliophorum* (1310 g, wet weight) was freeze-dried, then the dry material (106 g) was extracted exhaustively with dichloromethane and methanol (MeOH) to afford 38.3 g of residue after being dried under reduced pressure (Figure S51). The residue was partitioned with ethyl acetate (EtOAc) and water. The EtOAc soluble residue was subjected to silica gel flash chromatography column, using mixtures of *n*-hexane, EtOAc, and MeOH, with increasing polarity (*n*-hexane:EtOAc:MeOH, 100:0:0, 90:10:0, 84:16:0, 75:25:0, 65:35:0, 50:50:0, 0:100:0, 0:80:20) and the flow rate of 20–30 mL/min, to yield 20 fractions. After the MN-guided analyzing approach, fraction 12 was selected and then subjected to RP-HPLC, using acidic water (0.1% *v*/*v* acetic acid) and acetonitrile (CH_3_CN) with the ratio of 35:65 as the mobile phase for the isocratic mode elution, to afford glaucumolide A (**5**) (31.59 mg) (Figures S35–S37), glaucumolide B (**6**) (53.98 mg) (Figures S38–S40), and 13 other subfractions. Subfraction 1205 was purified with RP-HPLC, using 0.1% acetic acid solution:CH_3_CN (35:65) as the mobile phase for the isocratic mode elution, to yield sarcotrochelide A (**1**) (11.7 mg) and B (**2**) (2.05 mg). Subfraction 1211 was purified with RP-HPLC, using 0.1% acetic acid solution:MeOH (25:75) as the mobile phase for the isocratic mode elution, to yield bistrochelide B (**8**) (2.28 mg) (Figures S48–S50). Subfraction 1213 was purified with RP-HPLC, using 0.1% acetic acid solution: CH_3_CN (35:65) as the mobile phase for the isocratic mode elution, to yield bistrochelide A (**7**) (1.49 mg) (Figures S41–S47). Subfraction 1214 was purified with RP-HPLC, using 0.1% acetic acid solution:MeOH (25:75) as the mobile phase for the isocratic mode elution, to yield ximaolide A (**3**) (8.19 mg) (Figures S21–S27) and methyl tortuoate D (**4**) (2.29 mg) (Figures S28–S34).

Sarcotrochelide A (**1**) (Figures S1–S10 and S52): white powder; ${\left[\alpha \right]}_{D}^{25}$ = +145.0 (c 0.01, MeOH); UV (MeOH) λ_max_ 206 and 240 nm; IR (KBr) ν_max_ 3417, 2929, 1741, 1704, 1668, 1604, 1434, 1385, 1254, 1207, 1111, 1032 cm^−1^; ^13^C and ^1^H NMR data, Table 2; HRESIMS m/z 681.4362 [M + H]^+^(calcd. for C_41_H_60_O_8_ + H, 681.4366).

Sarcotrochelide B (**2**) (Figures S11–S20 and S53): white powder; ${\left[\alpha \right]}_{D}^{25}$ = +173.6 (c 0.01, MeOH); UV (MeOH) λ_max_ 206 and 240 nm; IR (KBr) ν_max_ 3417, 2923, 1738, 1701, 1667, 1601, 1416, 1372, 1257, 1206, 1110, 1060 cm^−1^; ^13^C and ^1^H NMR data, Table 3; HRESIMS m/z 699.4467 [M + H]^+^ (calcd. for C_41_H_62_O_9_ + H, 699.4472).

### 3.5. Preparation of Human Neutrophils

Venous blood sampling was performed on human donors (aged 20–30 years) according to an approved protocol (IRB No. 202002493A3). The purification of neutrophils was achieved according to a reported procedure [34].

### 3.6. Determination of Superoxide Anion (O_2_^•−^) Generation

Under the treatment of compounds **1**–**8**, the O_2_^•−^ generation of human neutrophils was determined by superoxidase dismutase (SOD) inhibitable reduction in ferricytochrome c as previously described [35].

### 3.7. Measurement of Elastase Release

MeO-Suc-Ala-Ala-Pro-Val-p-nitroanilide was used as a substrate in an elastase release assay to evaluate the degranulation of azurophilic granules as previously described [36].

### 3.8. Statistics

Statistical analysis was performed using Student’s *t*-test for calculations. *p* values < 0.05 were considered to be statistically significant.

## 4. Conclusions

The chemical investigation of the cultured soft coral *Sarcophyton trocheliophorum* led to the isolation of two novel metabolites **1**–**2**, along with six known analogues of biscembranoid **3**–**8**. The in vitro tests showed that compound **5**–**8** exhibited significant inhibitory effects on the superoxide anion generation and elastase release in fMLF/CB-induced human neutrophils. The difference in their bioactivities suggested the importance of the lactone rings and geometry of double bonds in the structures.

The discovery of two novel biscembranoids demonstrated the chemical diversity of this type of metabolite in the aquaculture *Sarcophyton trocheliophorum*. In addition, the two most bioactive compounds, glaucumolide A (**5**) and glaucumolide B (**6**), were obtained with relatively high quantity, measured at 31.59 mg and 53.98 mg, respectively. The results suggested that aquaculture of soft coral could be a prolific and sustainable resource for the drug discovery and development [37].

## Figures and Tables

**Table 1:** Effects of crude samples on superoxide anion generation and elastase release in fMLF/CB-induced human neutrophils.

Sample	Superoxide Anion		Elastase Release	
	IC_50_ (μg/mL) ^a^	Inh%	IC_50_ (μg/mL) ^a^	Inh%
DCM/MeOH extract		3.54 ± 1.96		38.95 ± 4.18 ***
EtOAc-soluble extract		18.97 ± 4.17 *		27.13 ± 8.44 *
Water-soluble extract ^b^		2.08 ± 1.90		−0.48 ± 0.70
Fraction 12	5.45 ± 0.66	70.40 ± 2.57 ***	7.48 ± 0.99	61.14 ± 4.59 ***

Percentage of inhibition (Inh%) at 10 μg/mL. Results are presented as mean ± S.E.M. (*n* = 3). * *p* < 0.05, *** *p* < 0.001 compared with the control (DMSO). ^a^ Concentration necessary for 50% inhibition (IC_50_). ^b^ The water fraction was dissolved in H_2_O.

**Table 2:** ^1^H (600 MHz, CDCl_3_) and ^13^C (150 MHz, CDCl_3_) NMR data for **1**.

Position	δ_H_ (*J* in Hz) ^a^	δ_C_ ^b^ Mult. ^c^	COSY	HMBC
1.		50.8, qC		
2	3.74, dd (8.3, 8.3)	44.6, CH	H-36	C-1, -3, -4, -6, -14, -20, -21, -35, -36
3		203.3, qC		
4	6.59, brs	126.7, CH	H-19	C-3, -6, -19
5		161.1, qC		
6	1.56–1.65, m; 3.25–3.31, m	32.4, CH_2_		C-4, -5, -7, -8, -19
7	1.53–1.59, m; 1.24–1.28, m	24.6, CH_2_	H-8	C-8, -9
8	1.63–1.71, m; 1.26–1.36, m	30.9, CH_2_	H-7, -9	C-9, -10
9	2.73–2.81, m	43.9, CH	H-8, -18	C-7, -8, -10, 18
10		214.8, qC		
11	2.71–2.80, m; 2.05–2.11, m	34.8, CH_2_	H-12	C-10, -12, -13, -15
12	2.95, ddd (10.0, 4.9, 2.8)	53.2, CH	H-11, -15	C-14, -15, -16
13		209.4, qC		
14	2.71–2.77, m; 3.05, d (18.6)	47.0, CH_2_		C-1, -2, -12, -13, -20, -21
15	2.14–2.23, m	29.2, CH	H-12, -16, -17	C-11, -12, -13, -17
16	0.74, d (6.8)	18.4, CH_3_	H-15	C-12, -15, -17
17	0.95, d (6.8)	21.2, CH_3_	H-15	C-12, -15, -16
18	1.06, d (6.8)	17.4, CH_3_	H-9	C-8, -9, -10
19	1.89, s	26.3, CH_3_	H-4	C-3, -4, -5, -6, -7
20		174.6, qC		
21	3.26, d (11.1)	43.2, CH	H-22	C-1, -2, -20, -22, -23, -33, -34
22	5.10, d (11.1)	126.7, CH	H-21, -38	C-1, -21, -24, -34, -38
23		134.0, qC		
24	2.19–2.30, m; 2.03–2.12, m	36.3, CH_2_	H-25	C-22, -23, -25, -26, -38
25	1.71–1.79, m; 1.47–1.58, m	26.3, CH_2_	H-24, -26	C-23, -24, -26, -27
26	2.91, dd (6.1, 4.3)	61.5, CH	H-25	C-24, -25, -27, -28
27		59.4, qC		
28	2.03–2.12, m	36.3, CH_2_	H-29	C-27, -29, -39
29	1.52–1.59, m; 1.60–1.66, m	24.0, CH_2_	H-28, -30	C-28
30	2.29, dd (8.8, 4.1)	60.8, CH	H-29	C-28, -29, -32
31		60.0, qC		
32	1.95, dd (14.5, 10.0); 1.78–1.88, m	39.7, CH_2_	H-33	C-30, -31, -33, -34, -40
33	4.79, dd (10.7, 2.1)	65.1, CH	H-32	C-21, -31, -32, -35
34		131.2, qC		
35		130.4, qC		
36	2.20–2.34, m	33.2, CH_2_	H-2	C-1, -2, -3, -34, -35, -37
37	1.74, s	18.7, CH_3_		C-1, -2, -21, -34, -35, -36
38	1.62, s	17.3, CH_3_	H-22	C-1, -21, -22, -23, -24
39	1.24, s	16.3, CH_3_		C-26, -27, -28
40	1.25, s	18.7, CH_3_		C-30, -31, -32
41	3.50, s	51.3, CH_3_		C-20

^a^ Spectroscopic data of **1** were recorded at 600 MHz in CDCl_3_. ^b^ Spectroscopic data of **1** was recorded at 150 MHz in CDCl_3_. ^c^ Attached protons were deduced by DEPT experiments.

**Table 3:** ^1^H (600 MHz, CDCl_3_) and ^13^C (150 MHz, CDCl_3_) NMR data for **2**.

Position	δ_H_ (*J* in Hz) ^a^	δ_C_ ^b^ Mult. ^c^	COSY	HMBC
1		50.5, qC		
2	3.45–3.49, m	44.1, CH	H-36	C-1, -3, -14, -20, -36
3		202.9, qC		
4	6.59, s	126.4, CH	H-19	C-3, -6, -19
5		161.4, qC		
6	1.59–1.67, m; 3.20–3.26, m	33.0, CH_2_	H-7	C-5, -7, -19
7	1.22–1.29, m; 1.53–1.60, m	25.0, CH_2_	H-6, -8	
8	1.75–1.82, m; 1.27–1.34, m	31.0, CH_2_	H-7, -9	
9	2.87–2.94, m	43.6, CH	H-8, -18	C-8, -10, -18
10		214.7, qC		
11	2.74, dd (16.2, 9.7); 2.13–2.21, m	34.6, CH_2_	H-12	C-10, -12, -13, -15
12	2.99–3.03, m	53.9, CH	H-11, -15	
13		208.7, qC		
14	3.18, d (18.2); 2.58, d (18.2)	46.0, CH_2_		C-1, -2, -13, -20, -21
15	2.32–2.38 m	28.6, CH	H-12, -16, -17	C-11, -12, -16, -17
16	0.74, d (6.5)	18.2, CH_3_	H-15	C-12, -15, -17
17	0.98, d (6.9)	21.4, CH_3_	H-15	C-12, -15, -16
18	1.07, d (6.6)	17.9, CH_3_	H-9	C-8, -9, -10
19	1.89, s	26.4, CH_3_	H-4	C-4, -5, -6, -7
20		174.9, qC		
21	3.67, d (10.8)	42.9, CH	H-22	C-1, -2, -14, -22, -23, -33, -34, -35
22	4.99, d (10.8)	128.0, CH	H-21, -38	C-24
23		137.2, qC		
24	2.08–2.17, m	37.0, CH_2_	H-25	C-22, -23, -25, -26, -38
25	1.91–2.00, m; 1.26–1.33, m	29.7, CH_2_	H-24, -26	C-23
26	3.24–3.30 m	74.1, CH	H-25	
27		86.0, qC		
28	2.34–2.40, m; 1.64–1.71, m	36.1, CH_2_	H-29	C-26, -27, -29, -30, -39
29	1.82–1.87, m; 1.54–1.60 m	27.0, CH_2_	H-28, -30	C-27, -28, -31
30	3.96, dd (10.3, 6.3)	88.4, CH	H-29	C-31, -32, -40
31		76.3, qC		
32	2.19–2.26, m; 1.03–1.08, m	39.5, CH_2_	H-33	C-31, -32, -40
33	5.05, d (11.2)	67.5, CH	H-32	C-21, -31, -32, -34, -35
34		125.3, qC		
35		132.2, qC		
36	2.41–2.51, m; 1.97–2.04, m	33.4, CH_2_	H-2, -37	C-1, -2, -34, -35, 37
37	1.61, s	18.1, CH_3_	H-36	C-21, -34, -35, -36
38	1.72, s	16.4, CH_3_	H-22	C-1, -22, -23, -24
39	1.17, s	20.6, CH_3_		C-26, -27, -28
40	1.19, s	21.4, CH_3_		C-30, -31, -32
41	3.50, s	51.1, CH_3_		C-20

^a^ Spectroscopic data of **2** were recorded at 600 MHz in CDCl_3_. ^b^ Spectroscopic data of **2** was recorded at 150 MHz in CDCl_3_. ^c^ Attached protons were deduced by DEPT experiments.

**Table 4:** Effects of compounds on superoxide anion generation and elastase release in fMLF/CB-induced human neutrophils.

Compound	Superoxide Anion		Elastase Release	
	IC_50_ (μM) ^a^	Inh%	IC_50_ (μM) ^a^	Inh%
Sarcotrochelide A (**1**)		16.92 ± 5.98 *		13.86 ± 5.87
Sarcotrochelide B (**2**)		10.15 ± 2.39 *		10.79 ± 4.60
Ximaolide A (**3**)		19.69 ± 5.00 *		26.64 ± 5.02 **
Methyl tortuoate D (**4**)		17.61 ± 1.99 ***		25.67 ± 5.27 **
Glaucumolide A (**5**)	5.46 ± 0.57	73.76 ± 3.84 ***	6.22 ± 0.36	67.50 ± 1.73 ***
Glaucumolide B (**6**)	1.98 ± 0.32	98.52 ± 0.50 ***	2.76 ± 0.47	101.94 ± 3.57 ***
Bistrochelide A (**7**)	8.29 ± 0.48	56.19 ± 2.83 ***		48.61 ± 0.96 ***
Bistrochelide B (**8**)		45.39 ± 4.30 ***		38.67 ± 4.81 **
LY294002 ^b^	1.62 ± 0.42	92.61 ± 3.81 ***	2.22 ± 0.49	86.85 ± 6.37 ***

Percentage of inhibition (Inh%) at 10 μM. Results are presented as mean ± S.E.M. (*n* = 3–5). * *p* < 0.05, ** *p* < 0.01, *** *p* < 0.001 compared with the control (DMSO). ^a^ Concentration necessary for 50% inhibition (IC_50_). ^b^ Positive control.

## Data Availability

The data presented in this study are available in the article.

## Supplementary Materials

The following supporting information can be downloaded at: https://www.mdpi.com/article/10.3390/ijms232415464/s1.
