# Supplementary material for: MS/MS Molecular Networking Unveils the Chemical Diversity of Biscembranoid Derivatives, Neutrophilic Inflammatory Mediators from the Cultured Soft Coral Sarcophyton trocheliophorum
Source: Int J Mol Sci. 2022 Dec 7;23(24):15464. doi: 10.3390/ijms232415464 (PMC9779164; doi:10.3390/ijms232415464)
Supplement: Supplementary file 1 [file ijms-23-15464-s001.zip › ijms-2025609-supplementary.pdf]

Supplementary Materials

MS/MS Molecular Networking Unveils the Chemical Diversity of Biscembranoid Derivatives, Neutrophilic Inflammatory Mediators from the Cultured Soft Coral Sarcophyton trocheliophorum

Ngoc Bao An Nguyen [1,†], Lo-Yun Chen [1,†], Po-Jen Chen [2], Mohamed El-Shazly [3], Tsong-Long Hwang [4,5,6,7,8], Jui-Hsin Su [9,10], Chun-Han Su [11], Pei-Tzu Yen [12,13], Bo-Rong Peng [1,14,*] and Kuei-Hung Lai [1,14,15,*]

1 Graduate Institute of Pharmacognosy, College of Pharmacy, Taipei Medical University, Taipei 11031, Taiwan
2 Department of Medical Research, E-Da Hospital, Kaohsiung 824005, Taiwan
3 Department of Pharmacognosy, Faculty of Pharmacy, Ain-Shams University, Organization of African Unity Street, Abassia, Cairo 11566, Egypt
4 Research Center for Chinese Herbal Medicine, College of Human Ecology, Chang Gung University of Science and Technology, Taoyuan 33303, Taiwan
5 Graduate Institute of Health Industry Technology, College of Human Ecology, Chang Gung University of Science and Technology, Taoyuan 33303, Taiwan
6 Graduate Institute of Natural Products, College of Medicine, Chang Gung University, Taoyuan 33302, Taiwan
7 Research Center for Food and Cosmetic Safety, College of Human Ecology, Chang Gung University of Science and Technology, Taoyuan 33303, Taiwan
8 Department of Chemical Engineering, Ming Chi University of Technology, New Taipei City 24301, Taiwan
9 National Museum of Marine Biology & Aquarium, Pingtung 94450, Taiwan
10 Department of Marine Biotechnology and Resources, National Sun Yat-sen University, Kaohsiung 80424, Taiwan
11 Department of Food Science, College of Human Ecology, Fu Jen Catholic University, New Taipei City, 24205, Taiwan
12 Jian Sheng Tang Chinese Medicine Clinic, Kaohsiung 80664, Taiwan
13 Crystal Clear Kampo Clinic, Tainan 70156, Taiwan
14 PhD Program in Clinical Drug Development of Herbal Medicine, College of Pharmacy, Taipei Medical University, Taipei 11031, Taiwan
15 Traditional Herbal Medicine Research Center, Taipei Medical University Hospital, Taipei 11031, Taiwan
* Correspondence: kueihunglai@tmu.edu.tw (K.-H.L.); peng_br@tmu.edu.tw (B.-R.P.); Tel.: +886-2-2736-1661 ext. 6157 (K.-H.L. & B.-R.P.)
† These authors contributed equally to this work.

Table of content

Figure S1. UV spectrum of Sarcotrochelide A (**1**) 4
Figure S2. IR spectrum of Sarcotrochelide A (**1**) 5
Figure S3. HRESIMS spectrum of Sarcotrochelide A (**1**) 5
Figure S4. ^{1}H NMR spectrum of Sarcotrochelide A (**1**) in $CDCl_3$ at 600 MHz 6
Figure S5. ^{13}C NMR spectrum of Sarcotrochelide A (**1**) in $CDCl_3$ at 150 MHz 7
Figure S6. DEPT spectrum of Sarcotrochelide A (**1**) in $CDCl_3$ at 150 MHz 8
Figure S7. HSQC spectrum of Sarcotrochelide A (**1**) in $CDCl_3$ 9
Figure S8. COSY spectrum of Sarcotrochelide A (**1**) in $CDCl_3$ 10
Figure S9. HMBC spectrum of Sarcotrochelide A (**1**) in $CDCl_3$ 11
Figure S10. NOESY spectrum of Sarcotrochelide A (**1**) in $CDCl_3$ 12
Figure S11. UV spectrum of Sarcotrochelide B (**2**) 13
Figure S12. IR spectrum of Sarcotrochelide B (**2**) 14
Figure S 13. HRESIMS spectrum of Sarcotrochelide B (**2**) 14
Figure S14. ^{1}H NMR spectrum of Sarcotrochelide B (**2**) in $CDCl_3$ at 600 MHz 15
Figure S15. ^{13}C NMR spectrum of Sarcotrochelide B (**2**) in $CDCl_3$ at 150 MHz 16
Figure S16. DEPT spectrum of Sarcotrochelide B (**2**) in $CDCl_3$ at 150 MHz 17
Figure S17. HSQC spectrum of Sarcotrochelide B (**2**) in $CDCl_3$ 18
Figure S18. COSY spectrum of Sarcotrochelide B (**2**) in $CDCl_3$ 19
Figure S19. HMBC spectrum of Sarcotrochelide B (**2**) in $CDCl_3$ 20
Figure S20. NOESY spectrum of Sarcotrochelide B (**2**) in $CDCl_3$ 21
Figure S21. ^{1}H NMR spectrum of Ximaolide A (**3**) in $CDCl_3$ at 600 MHz 22
Figure S22. ^{13}C NMR spectrum of Ximaolide A (**3**) in $CDCl_3$ at 150 MHz 23
Figure S23. DEPT spectrum of Ximaolide A (**3**) in $CDCl_3$ at 150 MHz 24
Figure S24. HSQC spectrum of Ximaolide A (**3**) in $CDCl_3$ 25
Figure S25. COSY spectrum of Ximaolide A (**3**) in $CDCl_3$ 26
Figure S26. HMBC spectrum of Ximaolide A (**3**) in $CDCl_3$ 27
Figure S27. NOESY spectrum of Ximaolide A (**3**) in $CDCl_3$ 28
Figure S28. ^{1}H NMR spectrum of Methyl tortuoate D (**4**) in $CDCl_3$ at 600 MHz 29
Figure S29. ^{13}C NMR spectrum of Methyl tortuoate D (**4**) in $CDCl_3$ at 150 MHz 30
Figure S30. DEPT spectrum of Methyl tortuoate D (**4**) in $CDCl_3$ at 150 MHz 31
Figure S31. HSQC spectrum of Methyl tortuoate D (**4**) in $CDCl_3$ 32
Figure S32. COSY spectrum of Methyl tortuoate D (**4**) in $CDCl_3$ 33
Figure S33. HMBC spectrum of Methyl tortuoate D (**4**) in $CDCl_3$ 34
Figure S34. NOESY spectrum of Methyl tortuoate D (**4**) in $CDCl_3$ 35
Figure S35. ^{1}H NMR spectrum of Glaucumolide A (**5**) in $CDCl_3$ at 600 MHz 36
Figure S36. ^{13}C NMR spectrum of Glaucumolide A (**5**) in $CDCl_3$ at 150 MHz 37
Figure S37. DEPT spectrum of Glaucumolide A (**5**) in $CDCl_3$ at 150 MHz 38

Figure S38. ^{1}H NMR spectrum of Glaucumolide B (**6**) in $CDCl_3$ at 600 MHz 39
Figure S39. ^{13}C NMR spectrum of Glaucumolide B (**6**) in $CDCl_3$ at 150 MHz 40
Figure S40. DEPT spectrum of Glaucumolide B (**6**) in $CDCl_3$ at 150 MHz 41
Figure S41. ^{1}H NMR spectrum of Bistrochelide A (**7**) in $CDCl_3$ at 600 MHz..................... 42
Figure S42. ^{13}C NMR spectrum of Bistrochelide A (**7**) in $CDCl_3$ at 150 MHz.................... 43
Figure S43. DEPT spectrum of Bistrochelide A (**7**) in $CDCl_3$ at 150 MHz 44
Figure S44. HSQC spectrum of Bistrochelide A (**7**) in $CDCl_3$... 45
Figure S45. COSY spectrum of Bistrochelide A (**7**) in $CDCl_3$.. 46
Figure S46. HMBC spectrum of Bistrochelide A (**7**) in $CDCl_3$.. 47
Figure S47. NOESY spectrum of Bistrochelide A (**7**) in $CDCl_3$... 48
Figure S48. ^{1}H NMR spectrum of Bistrochelide B (**8**) in $CDCl_3$ at 600 MHz 49
Figure S49. ^{13}C NMR spectrum of Bistrochelide B (**8**) in $CDCl_3$ at 150 MHz.................... 50
Figure S50. DEPT spectrum of Bistrochelide B (**8**) in $CDCl_3$ at 150 MHz 51
Figure S51. The process of isolation from soft coral Sarcophyton trocheliophorum....... 52
Figure S52. The LC-MS/MS fragment in negative mode of (**1**) 53
Figure S53. The LC-MS/MS fragment in negative mode of (**2**) 53

Report Date: 10:20:39, 09/27/2022

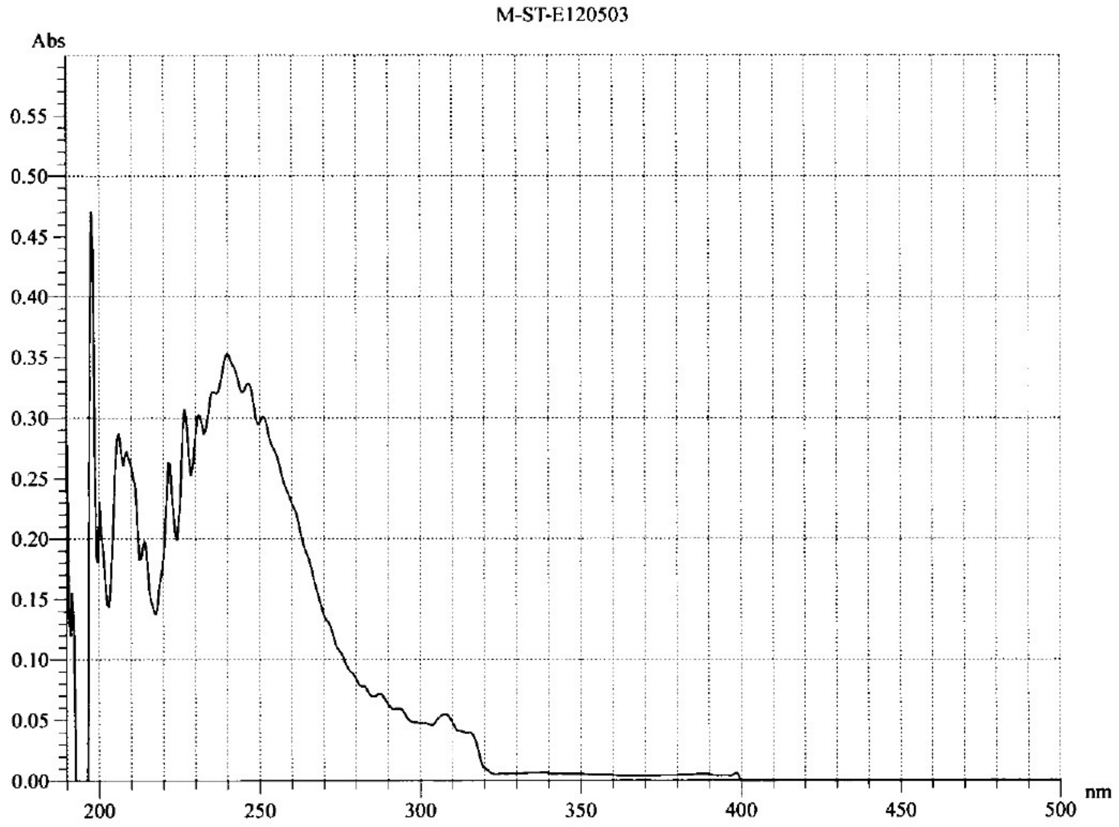

Sample: M-ST-E120503
File name: M-ST-E120503.UDS
Run Date: 10:16:55, 09/27/2022
Operator: Empower
Comment:

Peak Integration

Method: Rectangular
Sensitivity: 1
Threshold: 0.0100

Peaks

Peak #	Start (nm)	Apex (nm)	End (nm)	Height (Abs)	Area (Abs*nm)	Valley (nm)	Valley (/
1	663.60	663.10	402.10	0.0088	-2.0780	402.10	-0.0211
2	402.10	240.10	228.60	0.3532	14.9237	228.60	0.2529
3	228.60	226.60	224.10	0.3069	1.2972	224.10	0.1990
4	224.10	221.60	217.60	0.2634	1.4044	217.60	0.1375
5	217.60	206.10	203.10	0.2872	3.2287	203.10	0.1436
6	203.10	197.60	194.60	0.4708	0.8882	194.60	-0.4522

Figure S1. UV spectrum of Sarcotrochelide A (**1**)

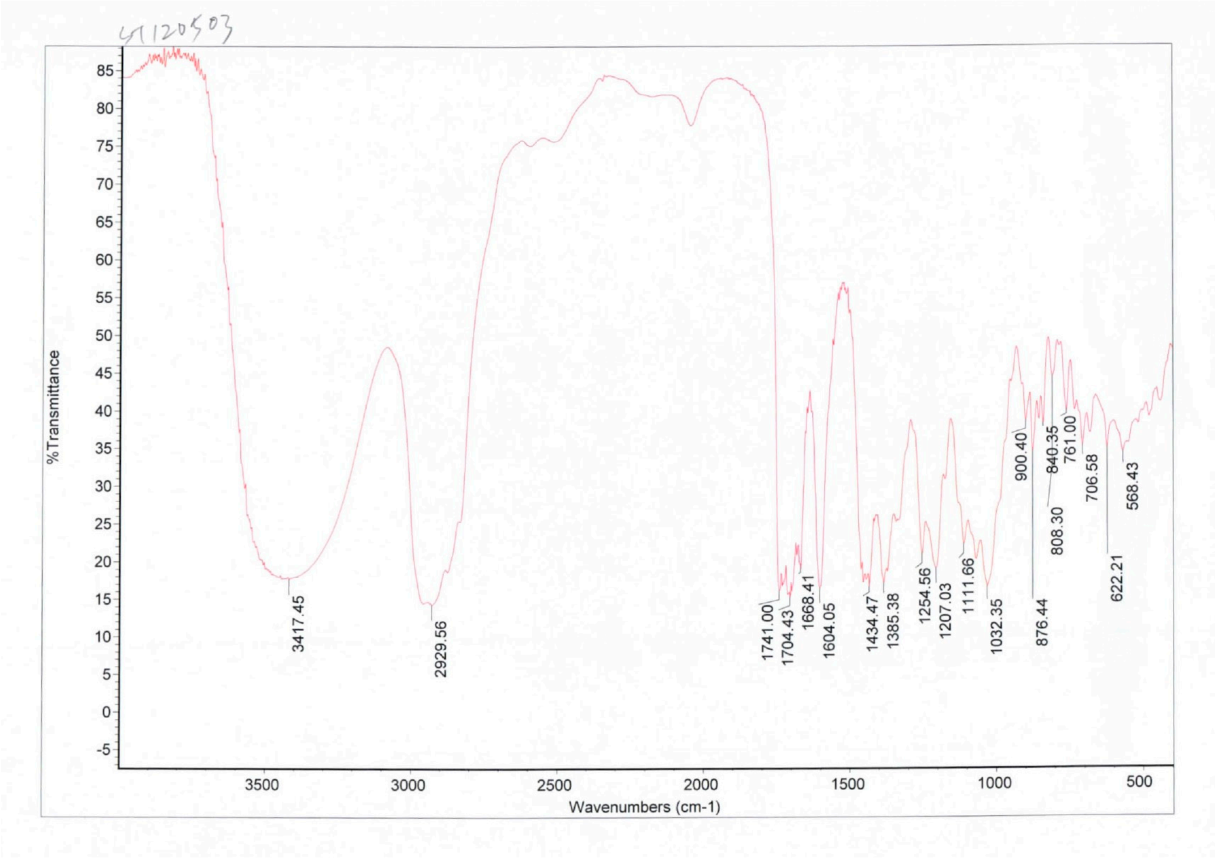

Figure S2. IR spectrum of Sarcotrochelide A (**1**)

Elemental Composition Report **Page 1**

Single Mass Analysis
Tolerance = 5.0 PPM / DBE: min = -1.5, max = 50.0
Element prediction: Off
Number of isotope peaks used for i-FIT = 3

Monoisotopic Mass, Even Electron Ions
106 formula(e) evaluated with 2 results within limits (up to 50 closest results for each mass)
Elements Used:
C: 0-50 H: 0-70 O: 0-10 Na: 0-1
20220818_pos_0503 185 (6.329) AM2 (Ar,18000.0,556.28,0.00,LS 3); ABS; Cm (181:186-147:176)
1: TOF MS ES+

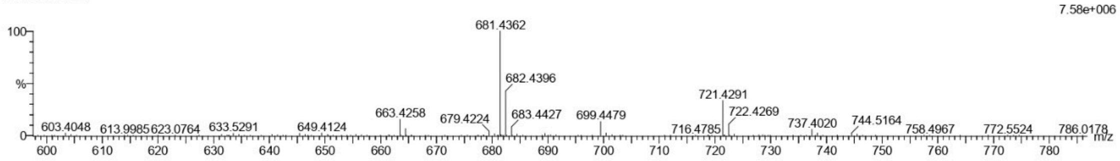

Minimum:				-1.5				
Maximum:		5.0	5.0	50.0				
Mass	Calc. Mass	mDa	PPM	DBE	i-FIT	Norm	Conf(%)	Formula
681.4362	681.4366	-0.4	-0.6	11.5	106.5	0.571	56.50	C41 H61 O8
	681.4342	2.0	2.9	8.5	106.7	0.833	43.50	C39 H62 O8 Na

Figure S3. HRESIMS spectrum of Sarcotrochelide A (**1**)

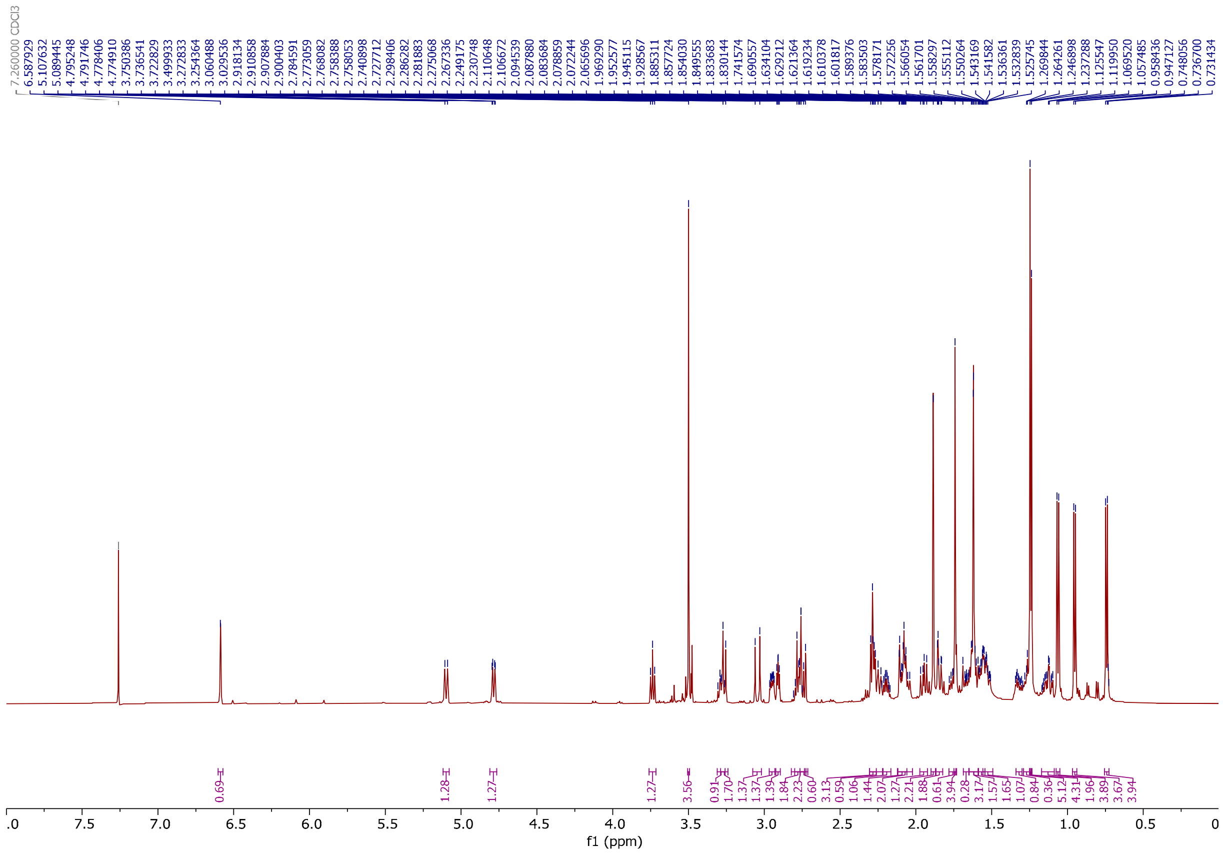

Figure S4. ^{1}H NMR spectrum of Sarcotrochelide A (**1**) in $CDCl_3$ at 600 MHz

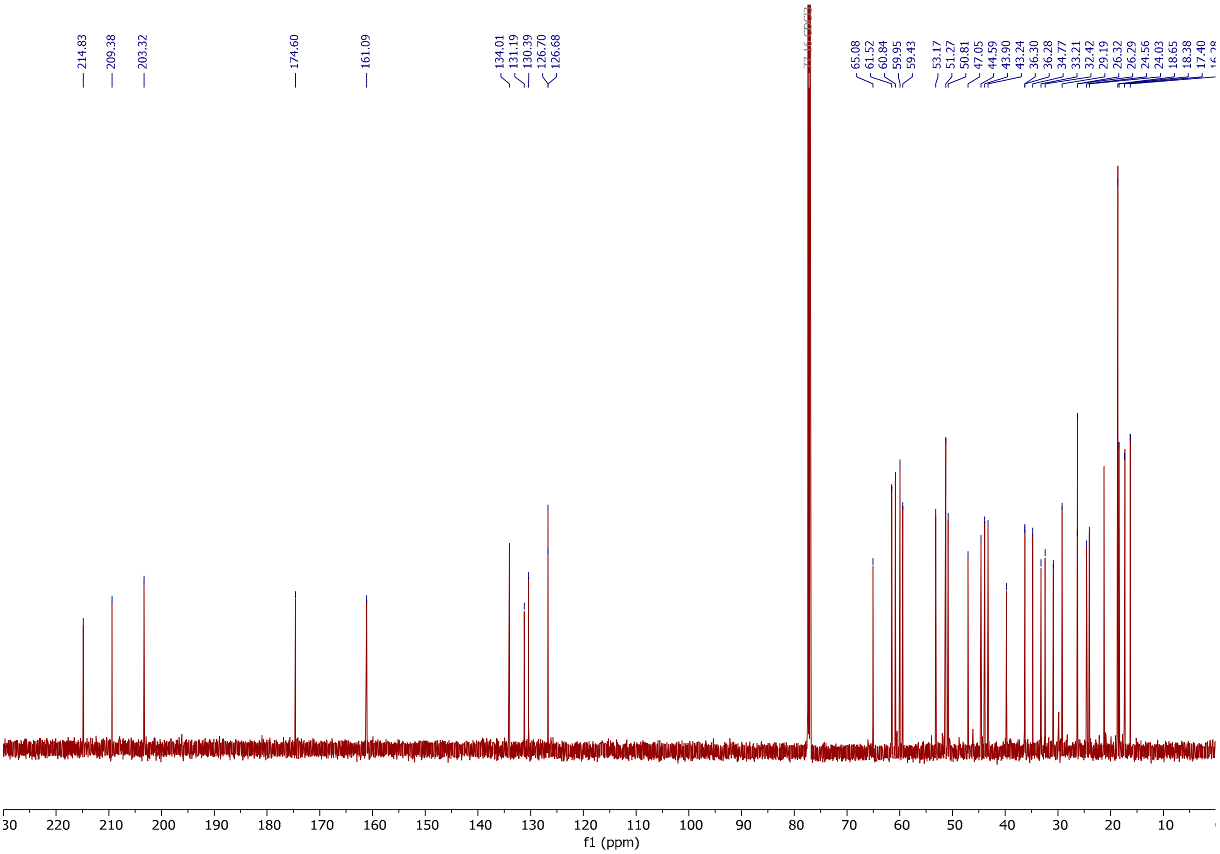

Figure S5. ^{13}C NMR spectrum of Sarcotrochelide A (**1**) in $CDCl_3$ at 150 MHz

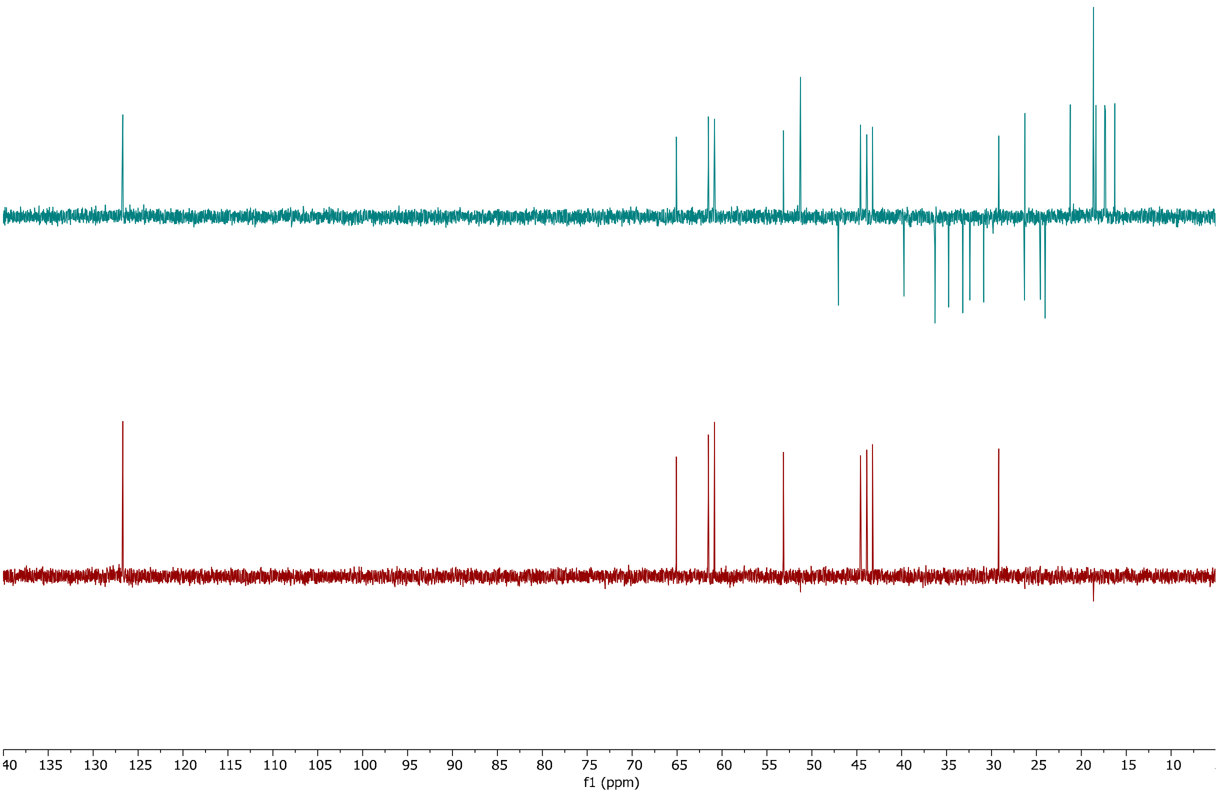

Figure S6. DEPT spectrum of Sarcotrochelide A (**1**) in $CDCl_3$ at 150 MHz

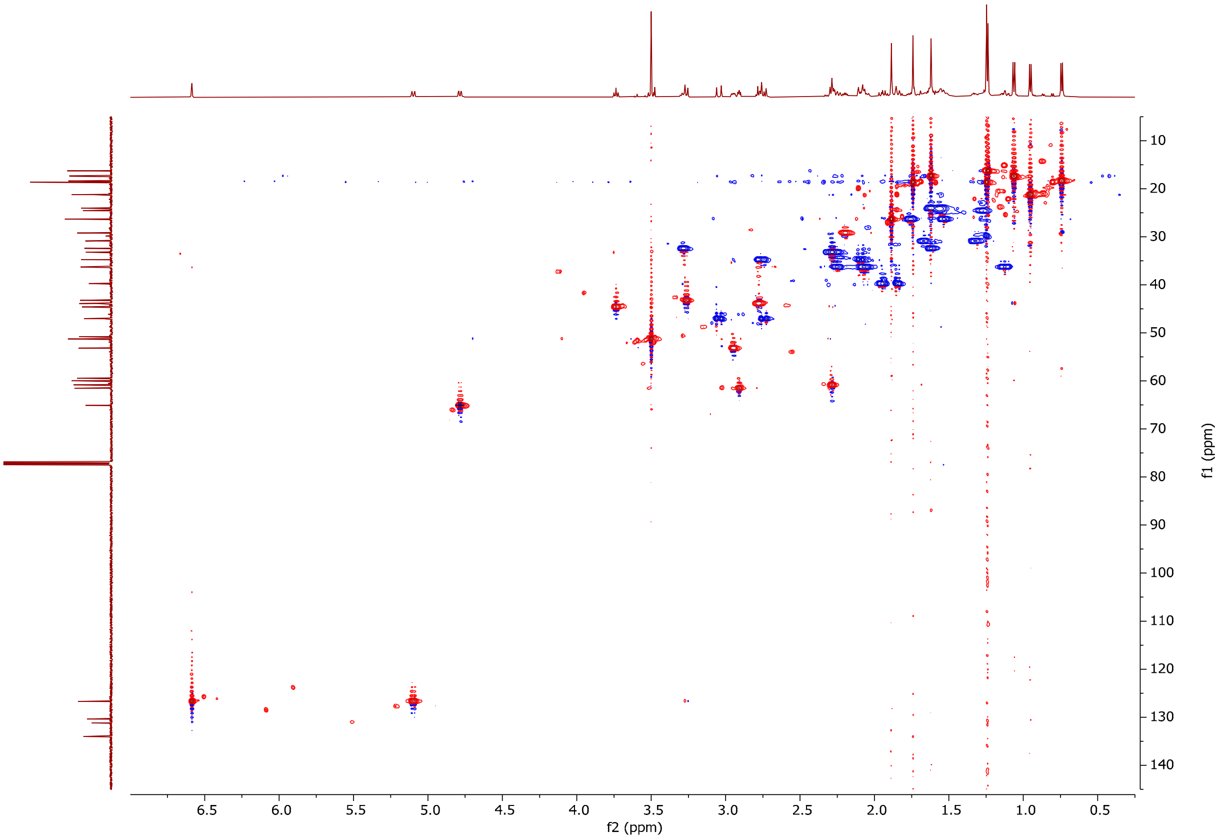

Figure S7. HSQC spectrum of Sarcotrochelide A (**1**) in $CDCl_3$

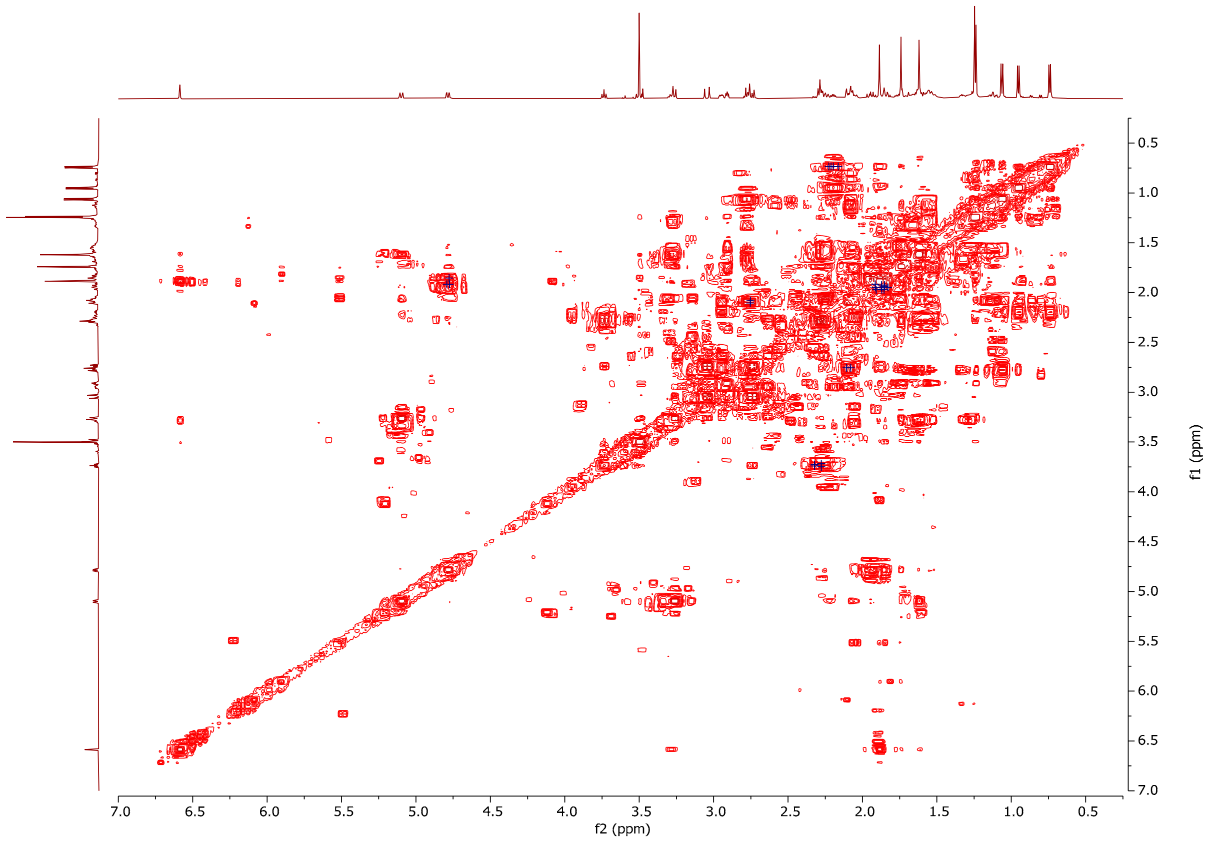

Figure S8. COSY spectrum of Sarcotrochelide A (**1**) in $CDCl_3$

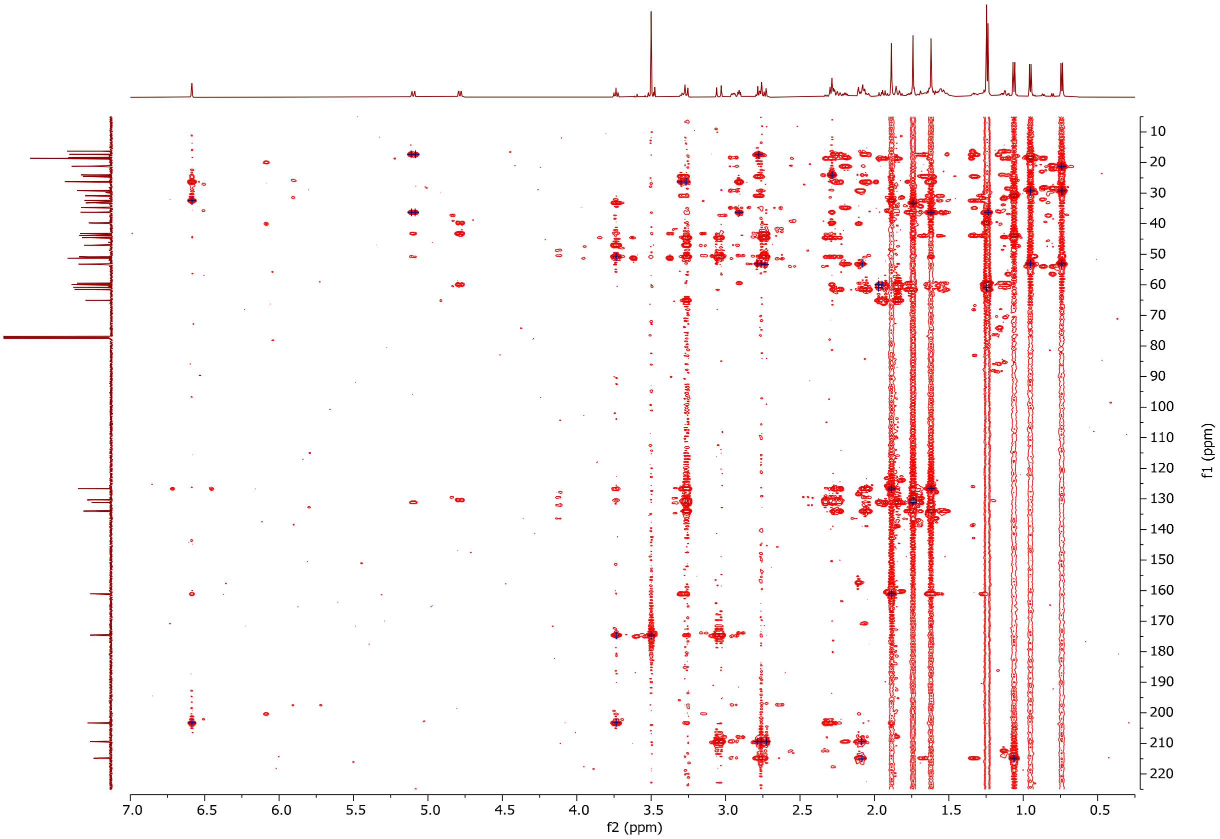

Figure S9. HMBC spectrum of Sarcotrochelide A (**1**) in $CDCl_3$

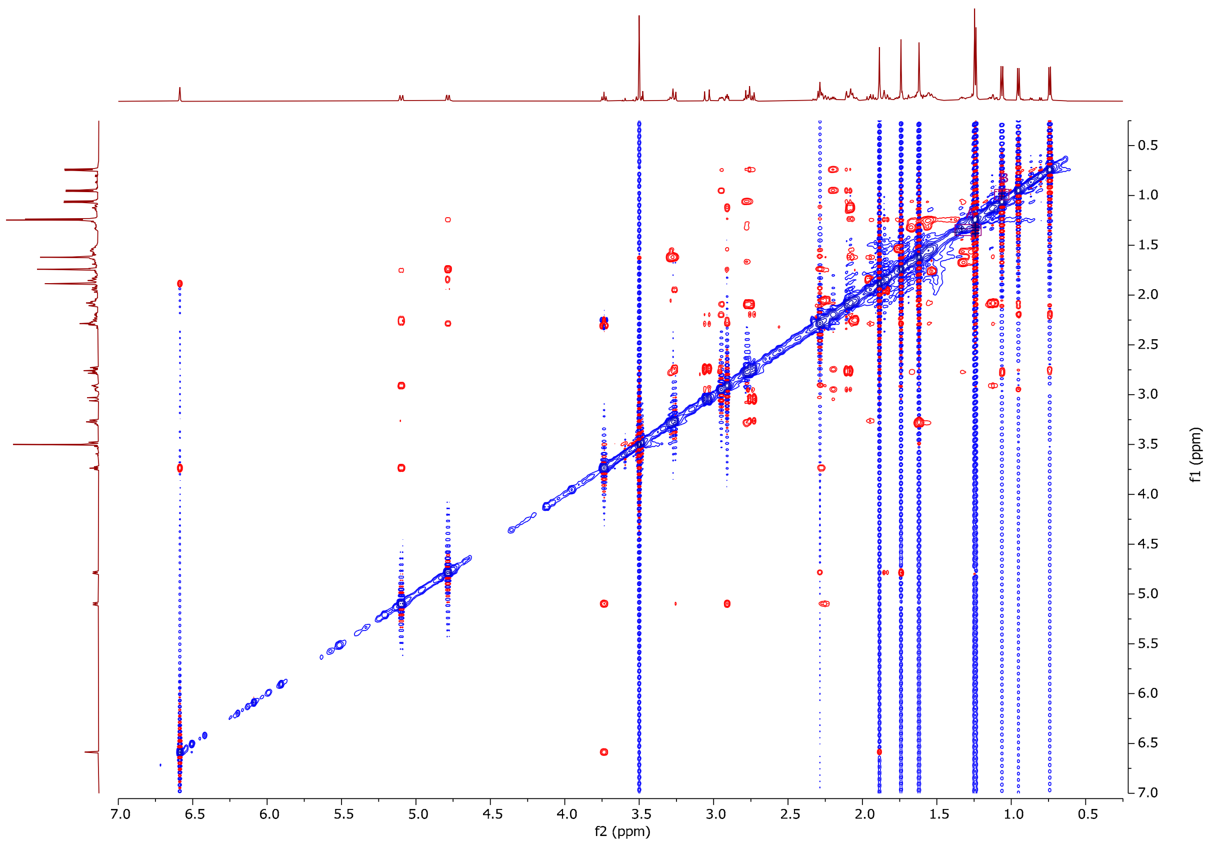

Figure S10. NOESY spectrum of Sarcotrochelide A (**1**) in $CDCl_3$

Report Date: 10:27:15, 09/27/2022

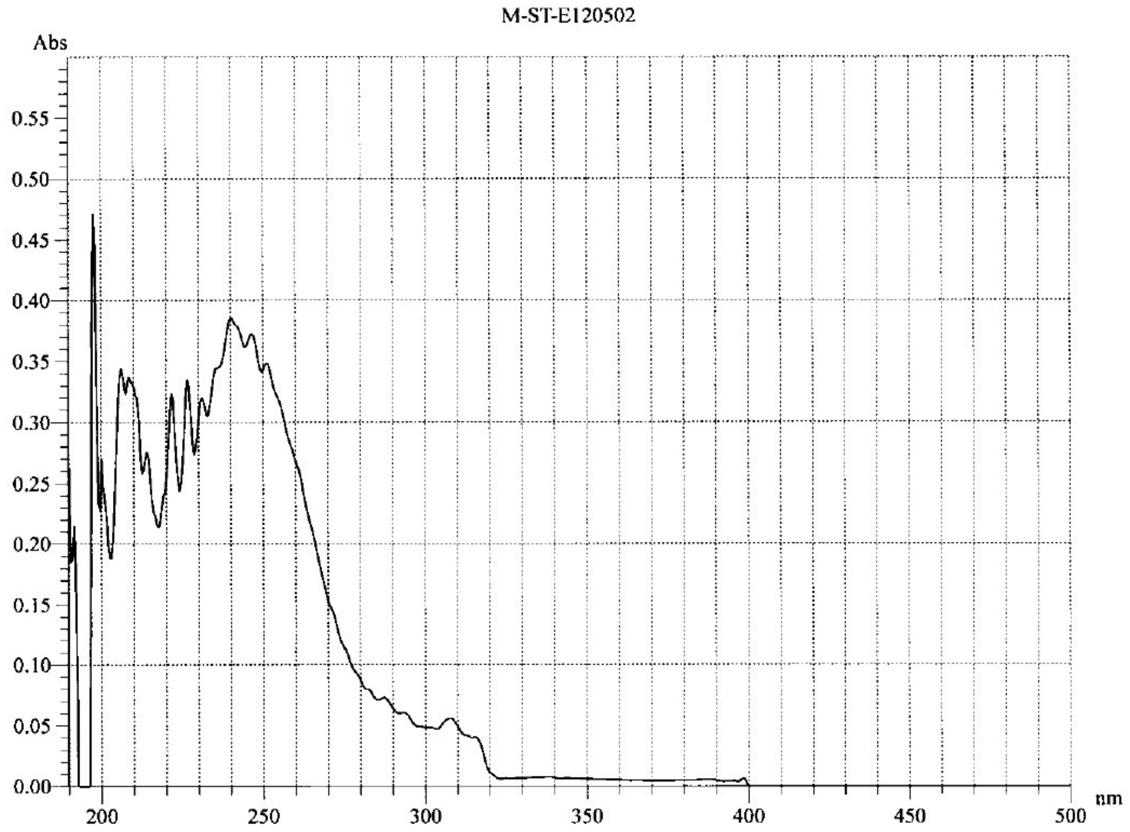

Sample: M-ST-E120502
File name: M-ST-E120502.UDS
Run Date: 10:24:00, 09/27/2022
Operator: Empower
Comment:

Peak Integration
Method: Rectangular
Sensitivity: 1
Threshold: 0.0100

Peaks

Peak #	Start (nm)	Apex (nm)	End (nm)	Height (Abs)	Area (Abs*nm)	Valley (nm)	Valley (/
1	663.60	663.60	402.10	0.0085	-2.0485	402.10	-0.0211
2	402.10	240.10	228.60	0.3858	16.5096	228.60	0.2734
3	228.60	226.60	224.10	0.3354	1.4531	224.10	0.2436
4	224.10	221.60	217.60	0.3238	1.8416	217.60	0.2130
5	217.60	206.10	203.10	0.3441	4.2360	203.10	0.1884
6	203.10	197.60	194.60	0.4711	1.2803	194.60	-0.3917

Figure S11. UV spectrum of Sarcotrochelide B (**2**)

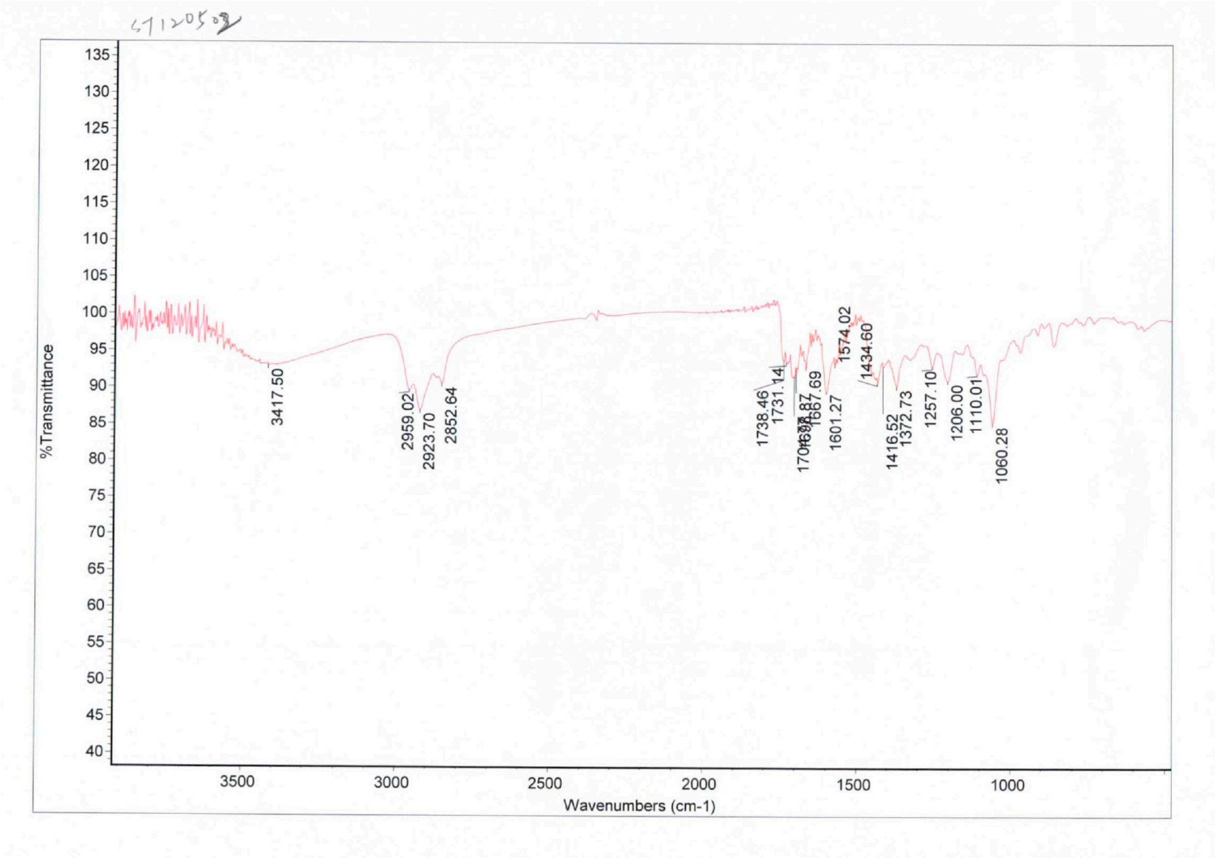

Figure S12**.** IR spectrum of Sarcotrochelide B (**2**)

Elemental Composition Report **Page 1**

Single Mass Analysis
Tolerance = 5.0 PPM / DBE: min = -1.5, max = 50.0
Element prediction: Off
Number of isotope peaks used for i-FIT = 3

Monoisotopic Mass, Even Electron Ions
90 formula(e) evaluated with 2 results within limits (up to 50 closest results for each mass)
Elements Used:
C: 0-50 H: 0-70 O: 0-10 Na: 0-1
20220818_pos_0502 175 (6.302) AM2 (Ar,18000.0,556.28,0.00,LS 3); ABS; Cm (175:176-157:172)
1: TOF MS ES+

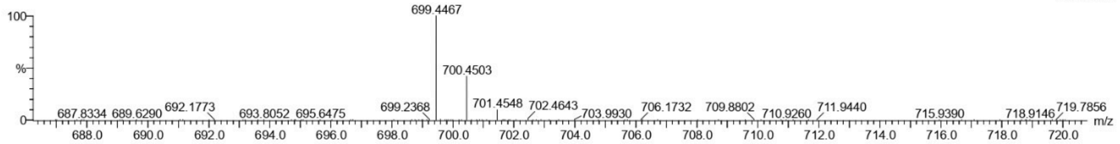

Minimum:				-1.5				
Maximum:		5.0	5.0	50.0				
Mass	Calc. Mass	mDa	PPM	DBE	i-FIT	Norm	Conf(%)	Formula
699.4467	699.4472	-0.5	-0.7	10.5	210.8	1.308	27.04	C41 H63 O9
	699.4448	1.9	2.7	7.5	209.8	0.315	72.96	C39 H64 O9 Na

Figure S13. HRESIMS spectrum of Sarcotrochelide B (**2**)

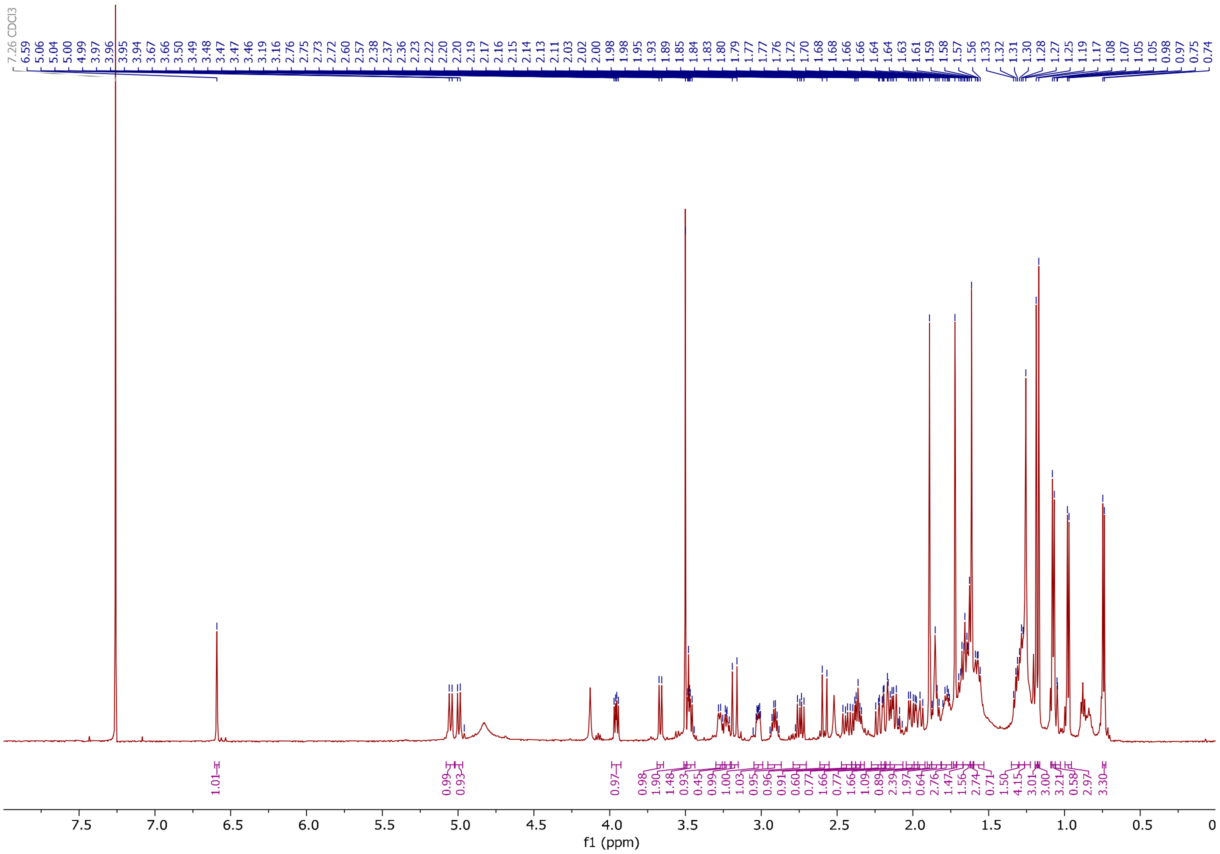

Figure S14. ^{1}H NMR spectrum of Sarcotrochelide B (**2**) in $CDCl_3$ at 600 MHz

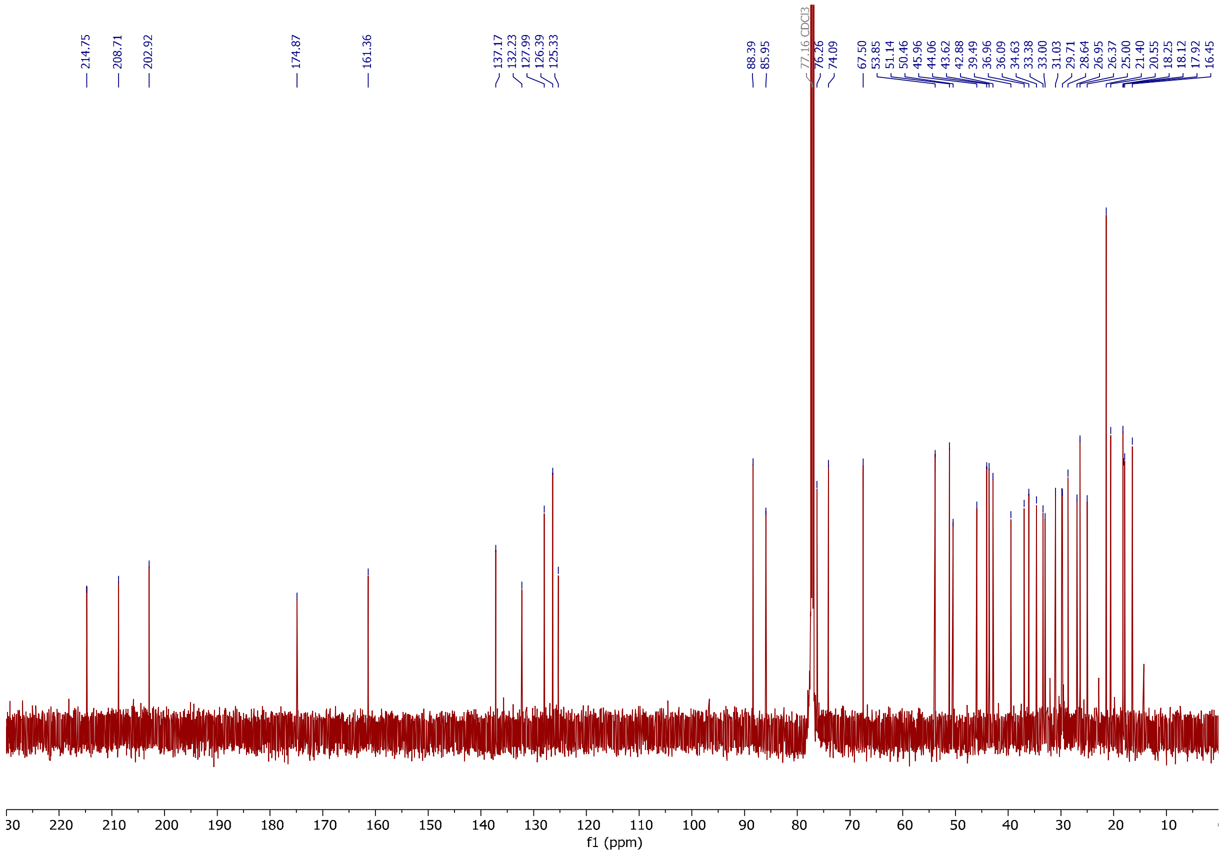

Figure S15. ^{13}C NMR spectrum of Sarcotrochelide B (**2**) in $CDCl_3$ at 150 MHz

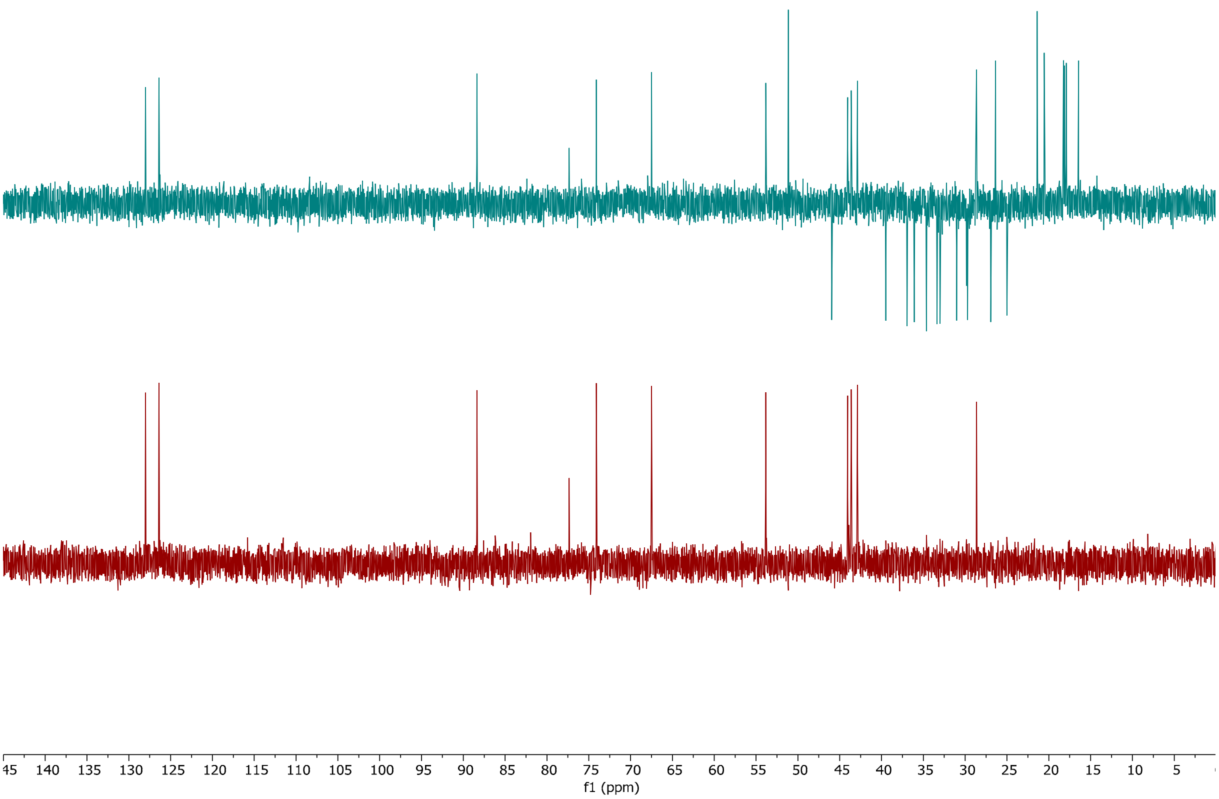

Figure S16. DEPT spectrum of Sarcotrochelide B (**2**) in $CDCl_3$ at 150 MHz

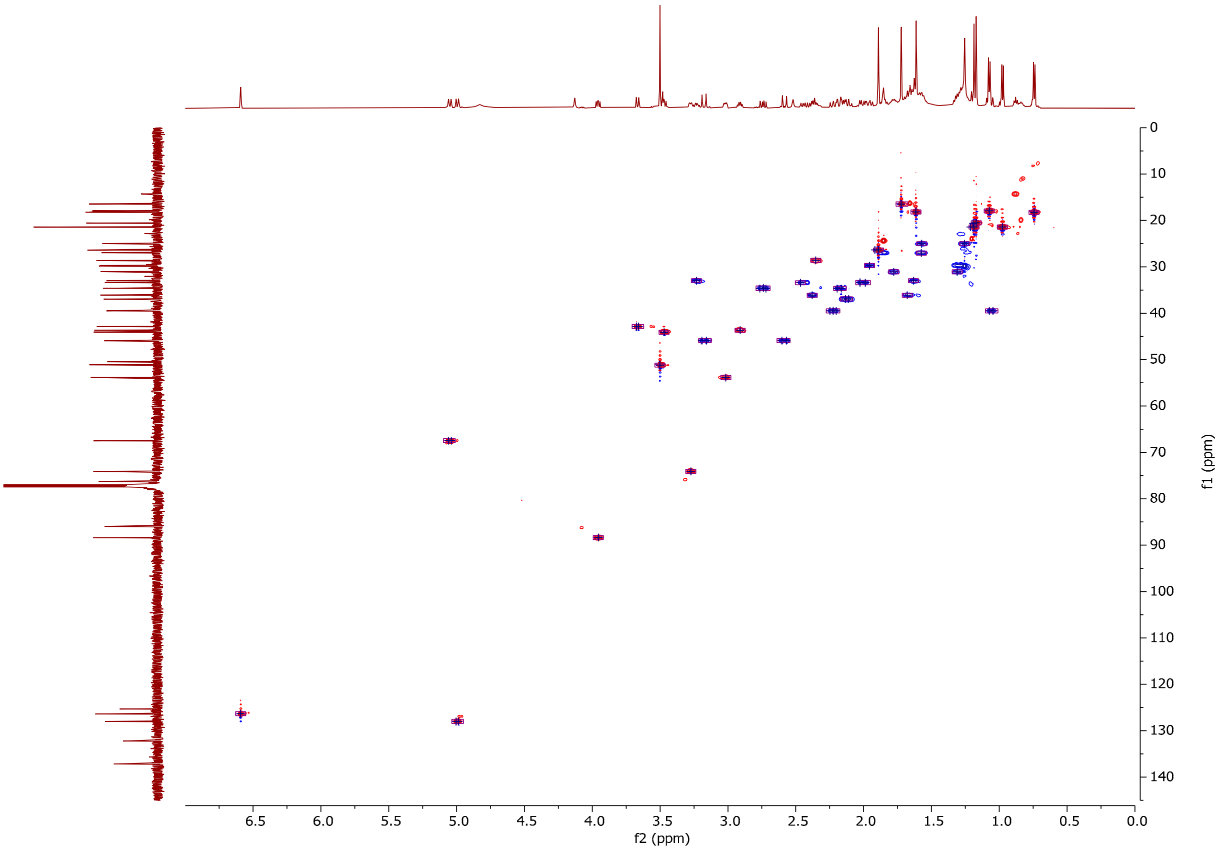

Figure S17. HSQC spectrum of Sarcotrochelide B (**2**) in $CDCl_3$

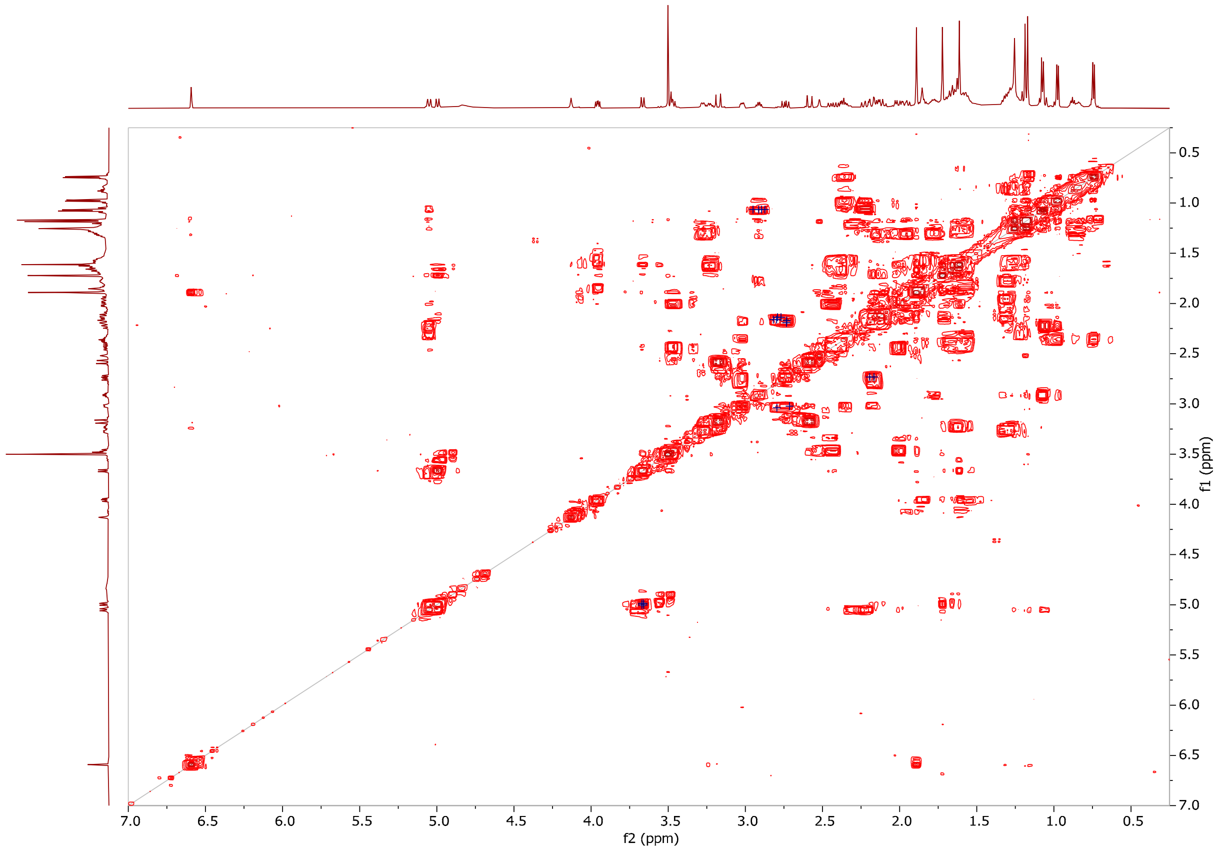

Figure S18. COSY spectrum of Sarcotrochelide B (**2**) in $CDCl_3$

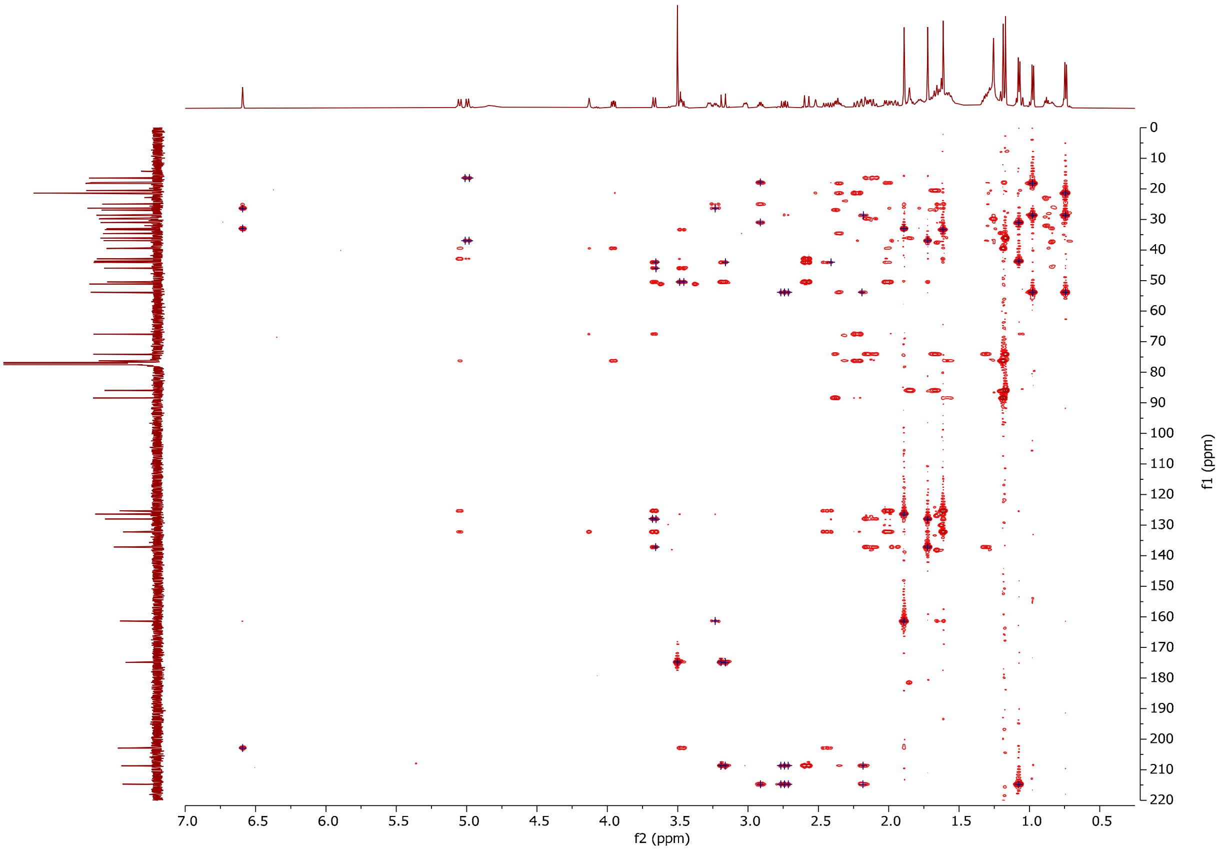

Figure S19. HMBC spectrum of Sarcotrochelide B (**2**) in $CDCl_3$

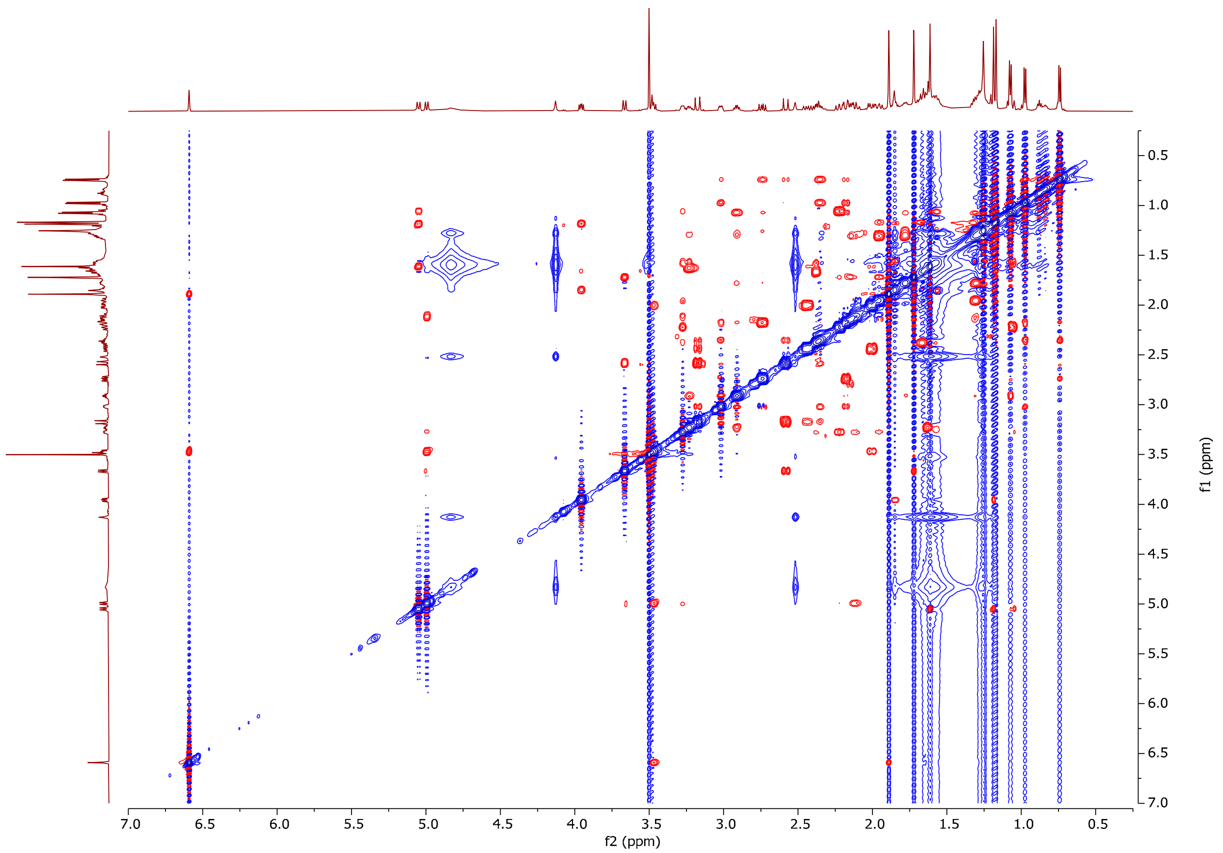

Figure S20. NOESY spectrum of Sarcotrochelide B (**2**) in $CDCl_3$

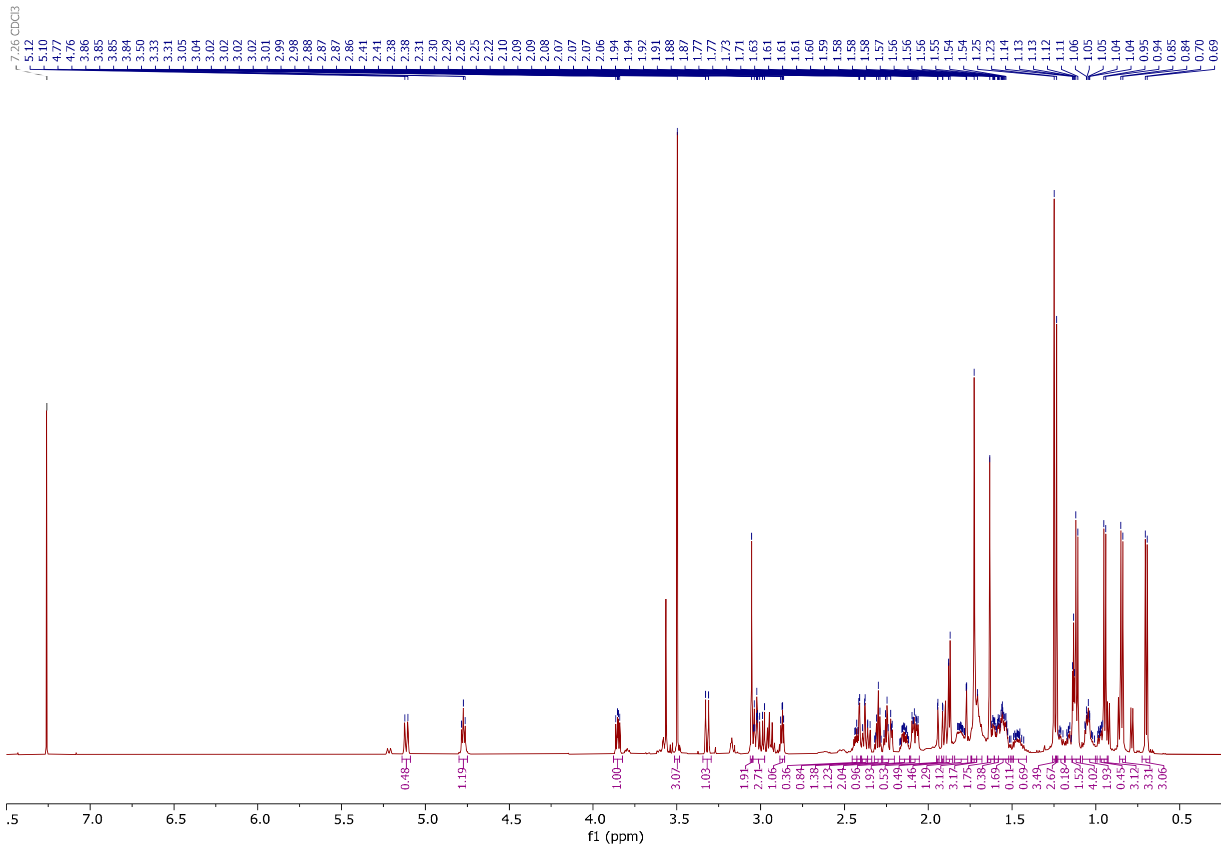

Figure S21. ^{1}H NMR spectrum of Ximaolide A (**3**) in $CDCl_3$ at 600 MHz

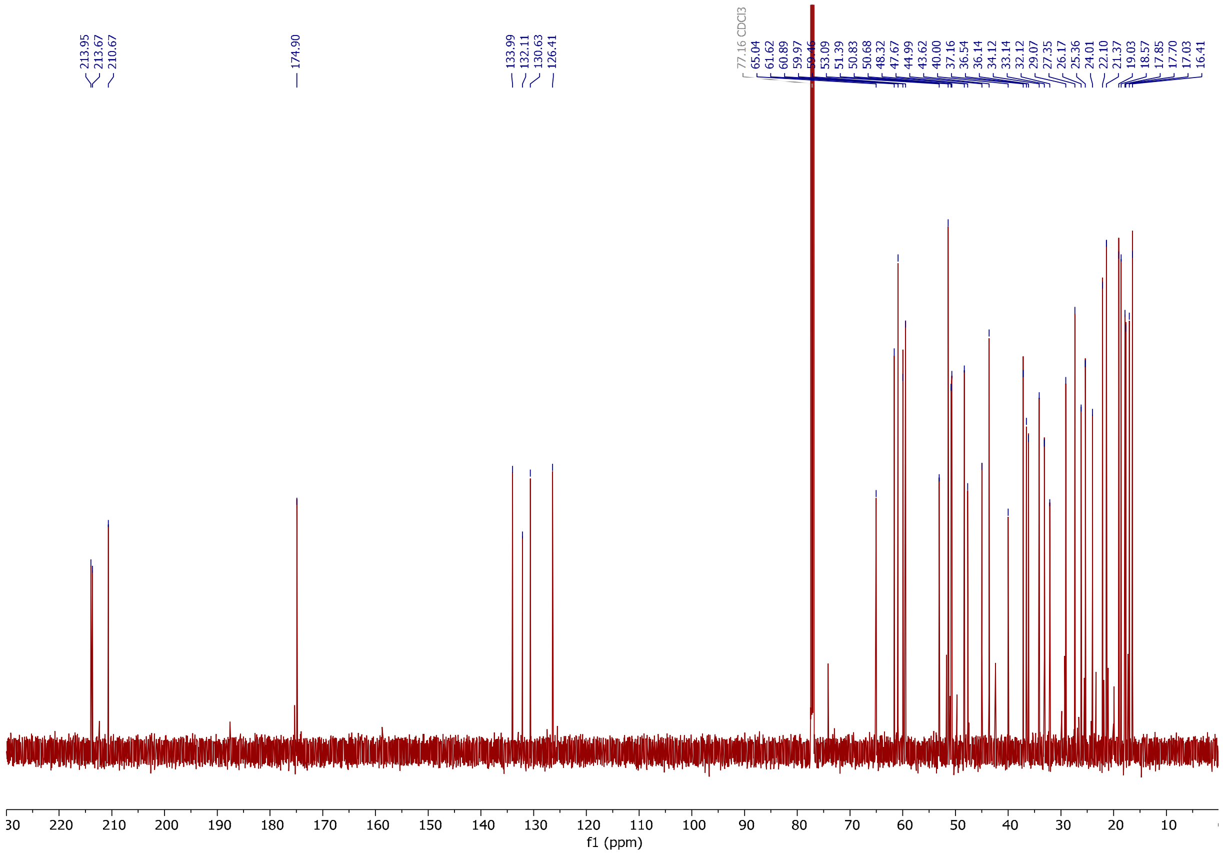

Figure S22. ^{13}C NMR spectrum of Ximaolide A (**3**) in $CDCl_3$ at 150 MHz

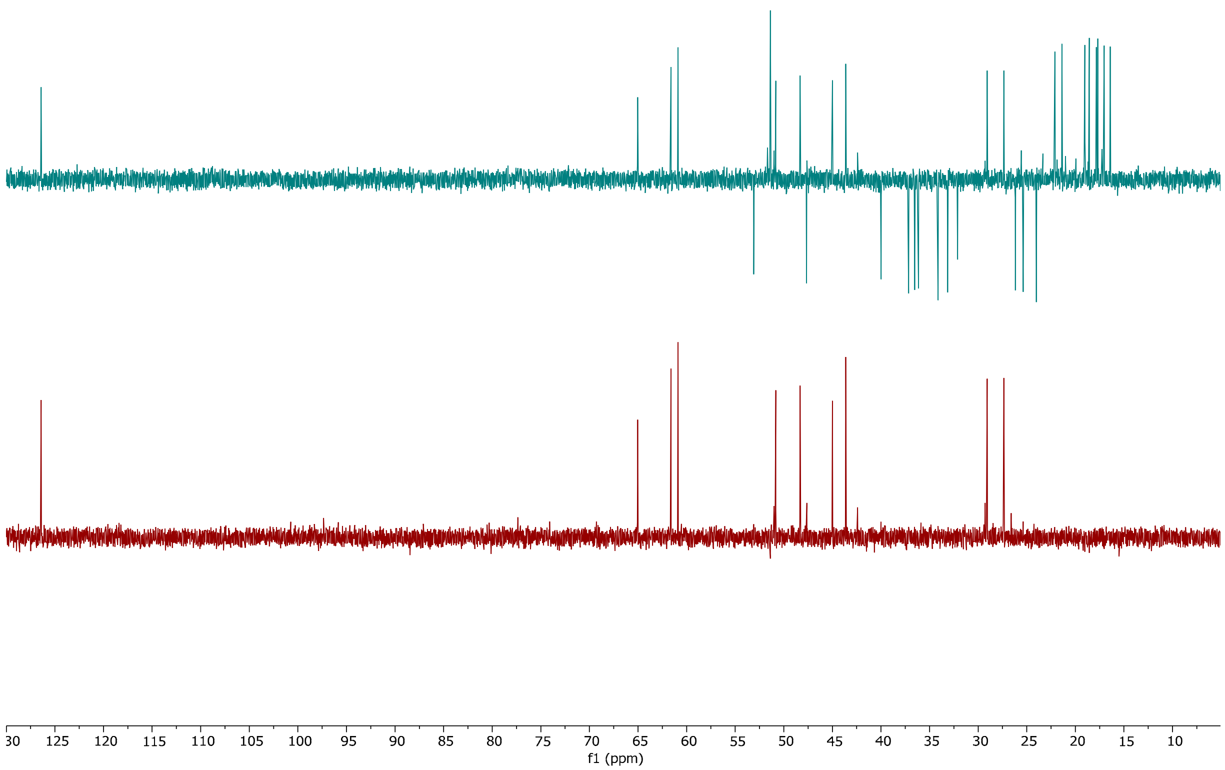

Figure S23. DEPT spectrum of Ximaolide A (**3**) in $CDCl_3$ at 150 MHz

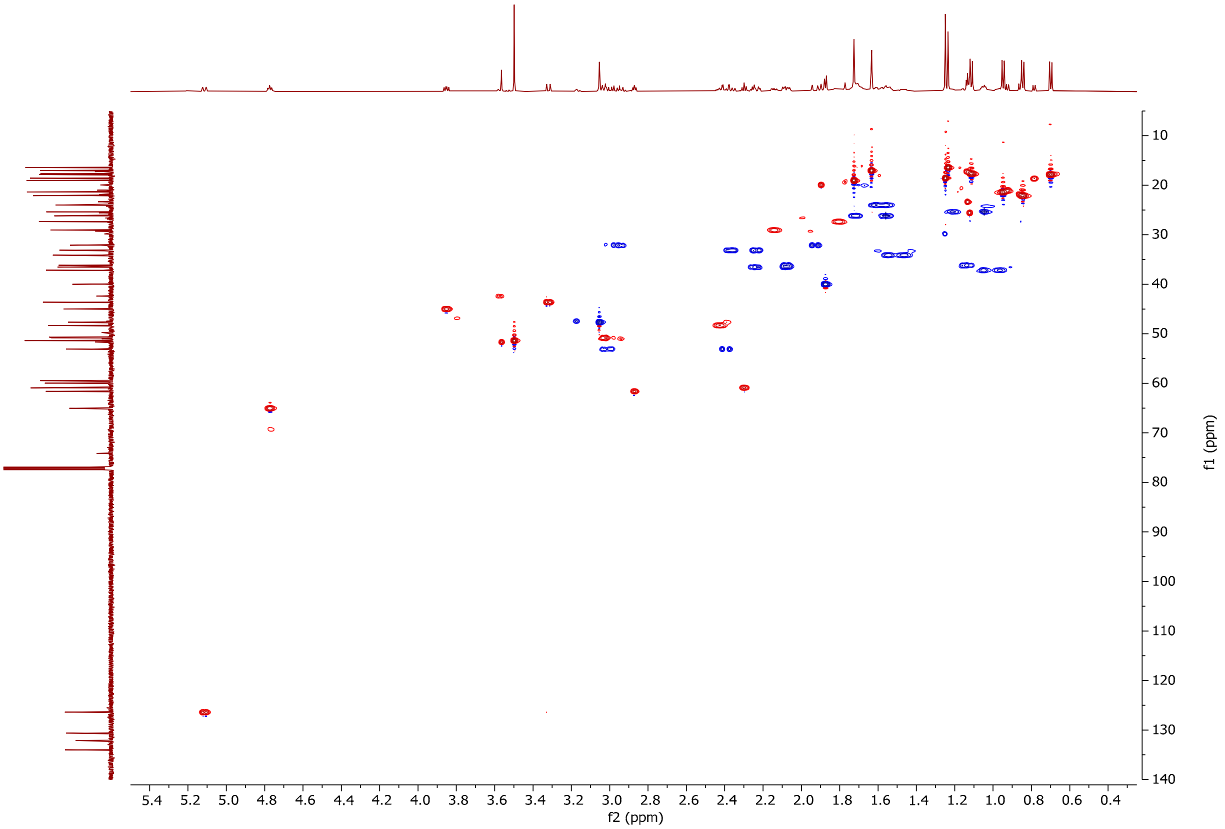

Figure S24. HSQC spectrum of Ximaolide A (**3**) in $CDCl_3$

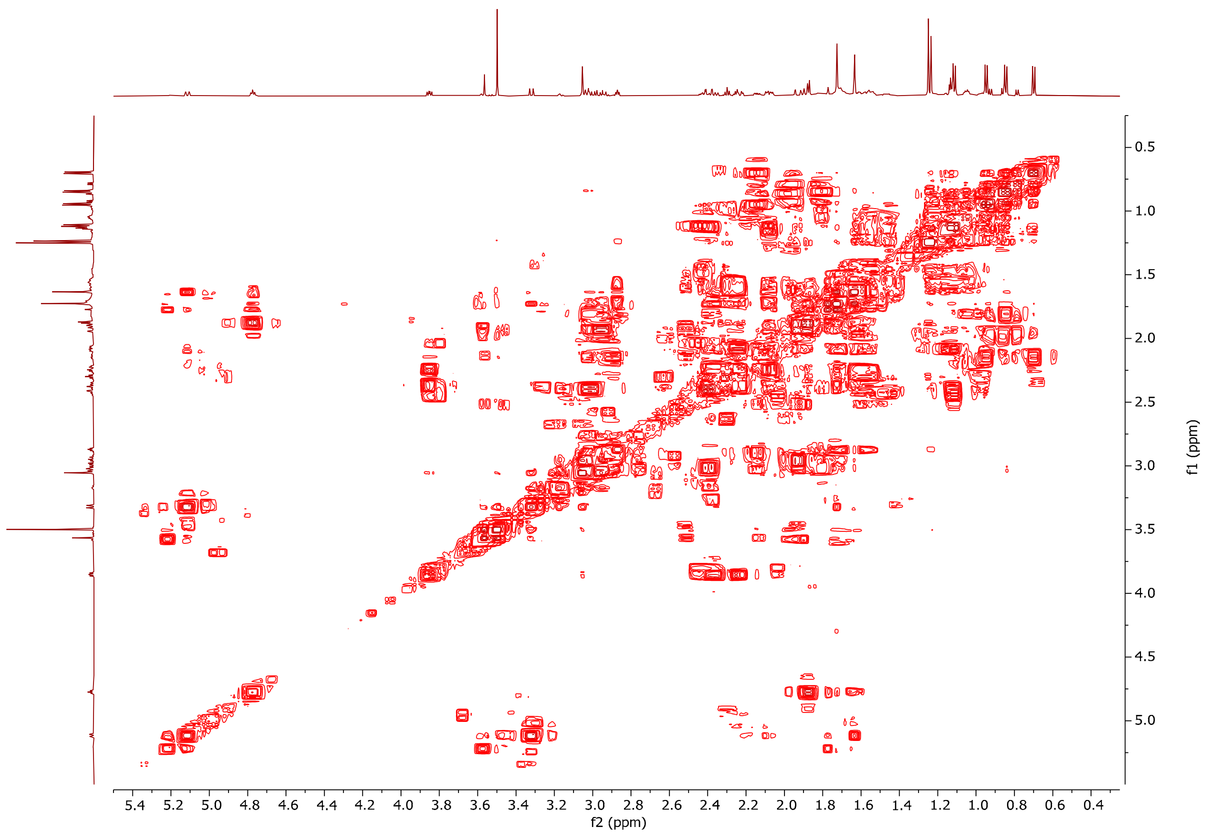

Figure S25. COSY spectrum of Ximaolide A (**3**) in $CDCl_3$

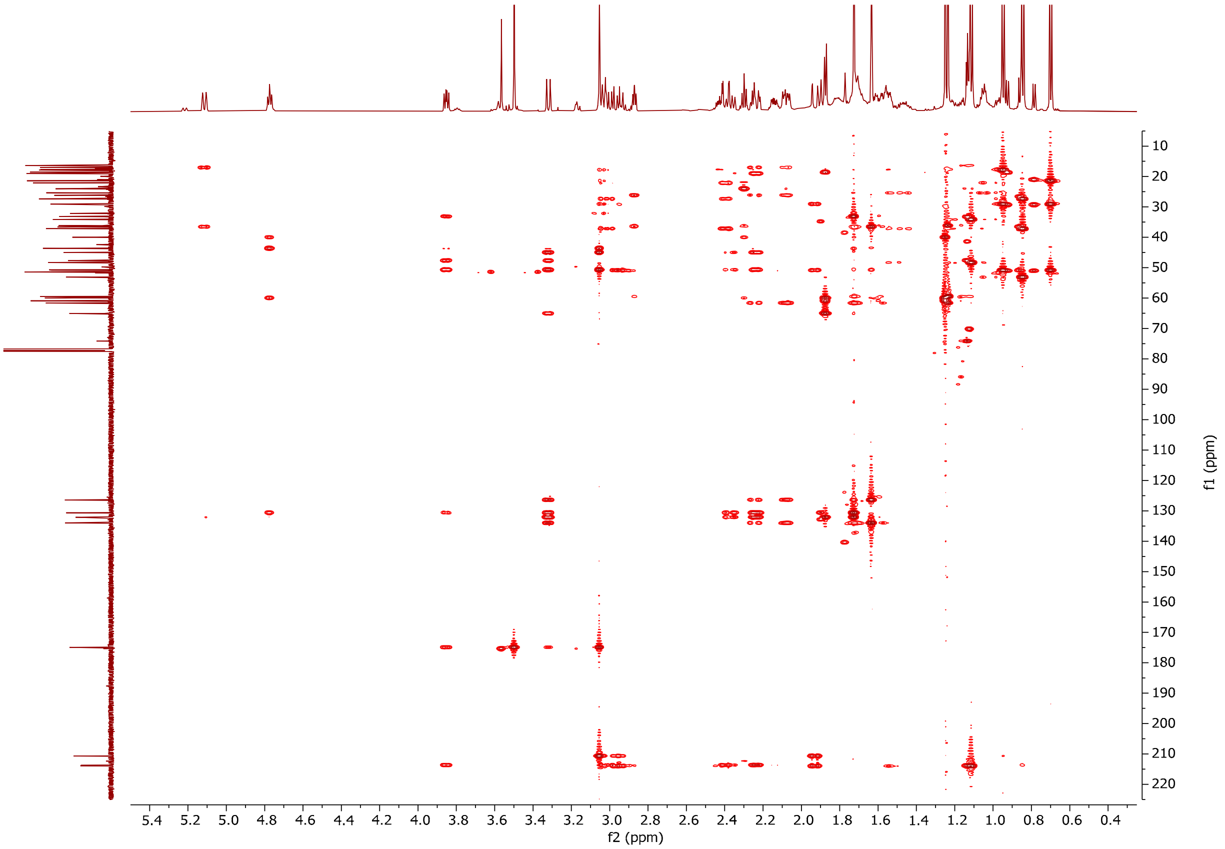

Figure S26. HMBC spectrum of Ximaolide A (**3**) in $CDCl_3$

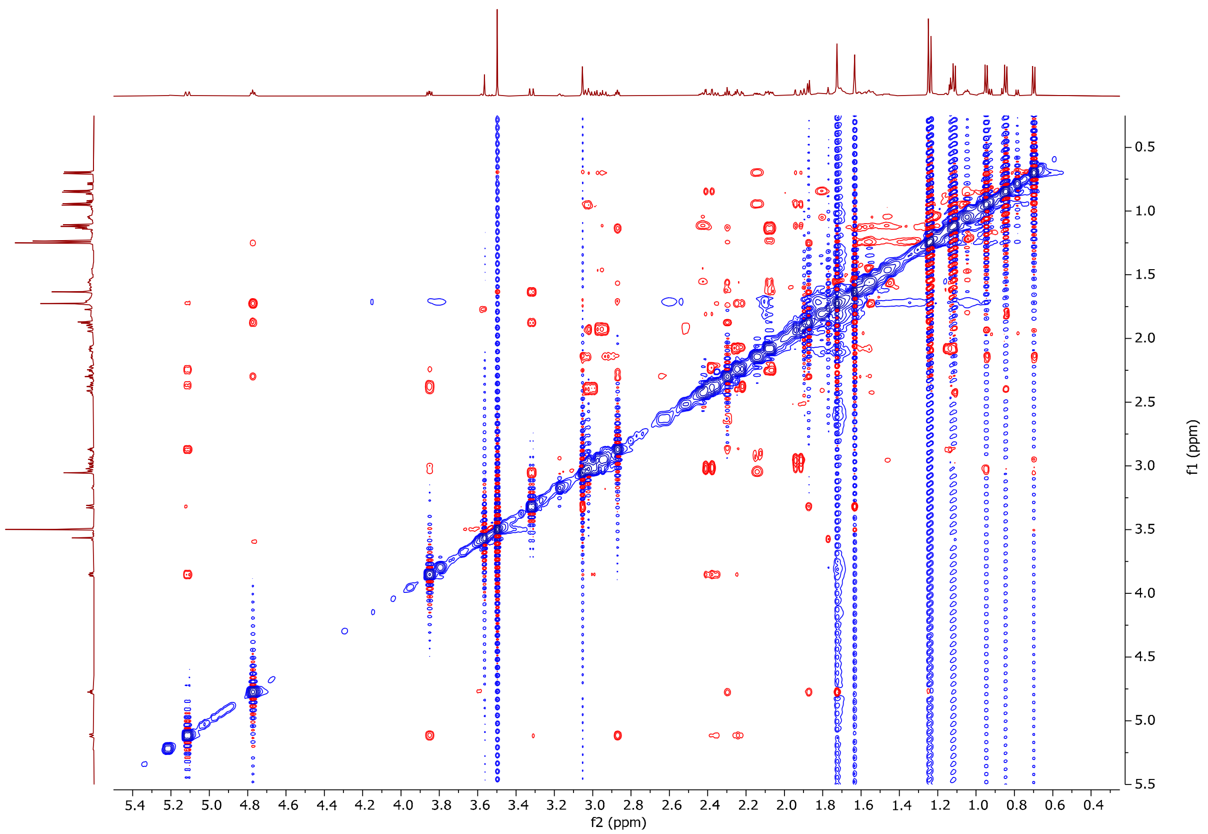

Figure S27. NOESY spectrum of Ximaolide A (**3**) in $CDCl_3$

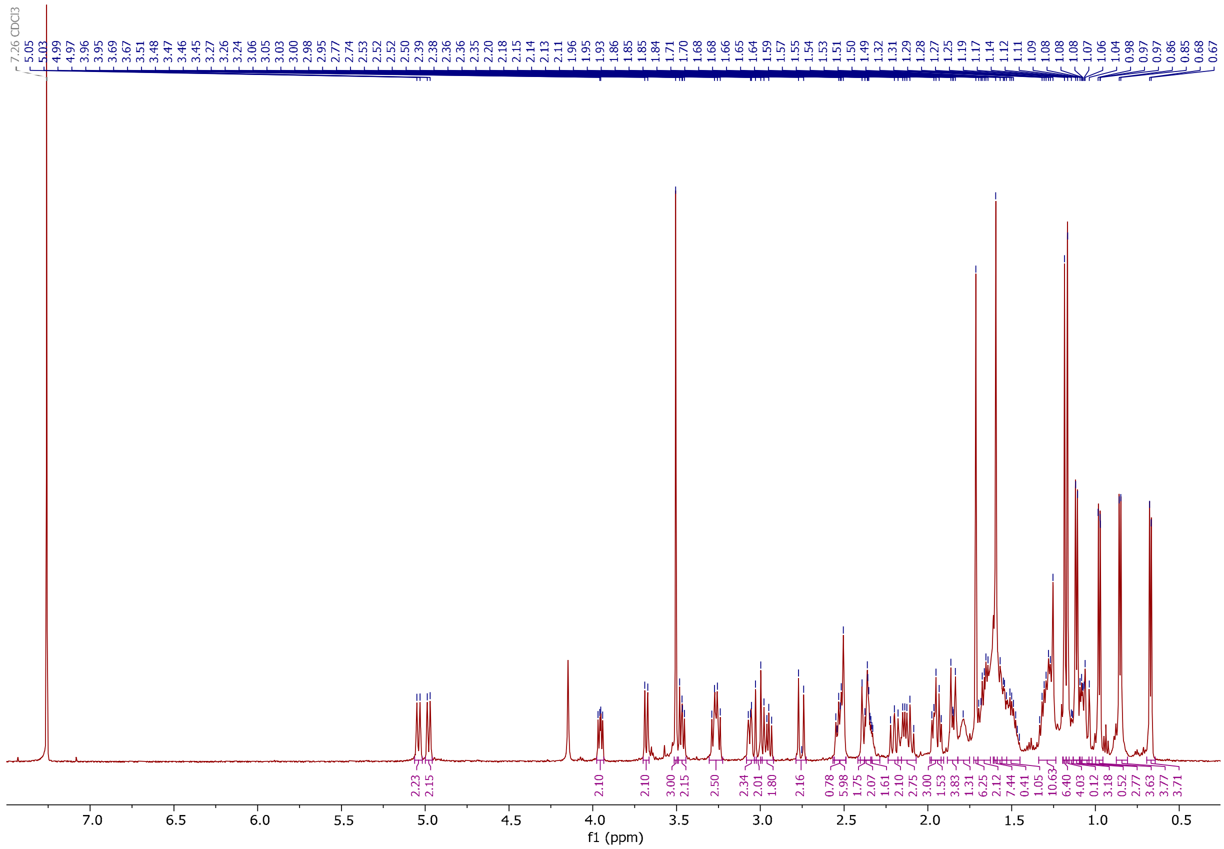

Figure S28. ^{1}H NMR spectrum of Methyl tortuoate D (**4**) in $CDCl_3$ at 600 MHz

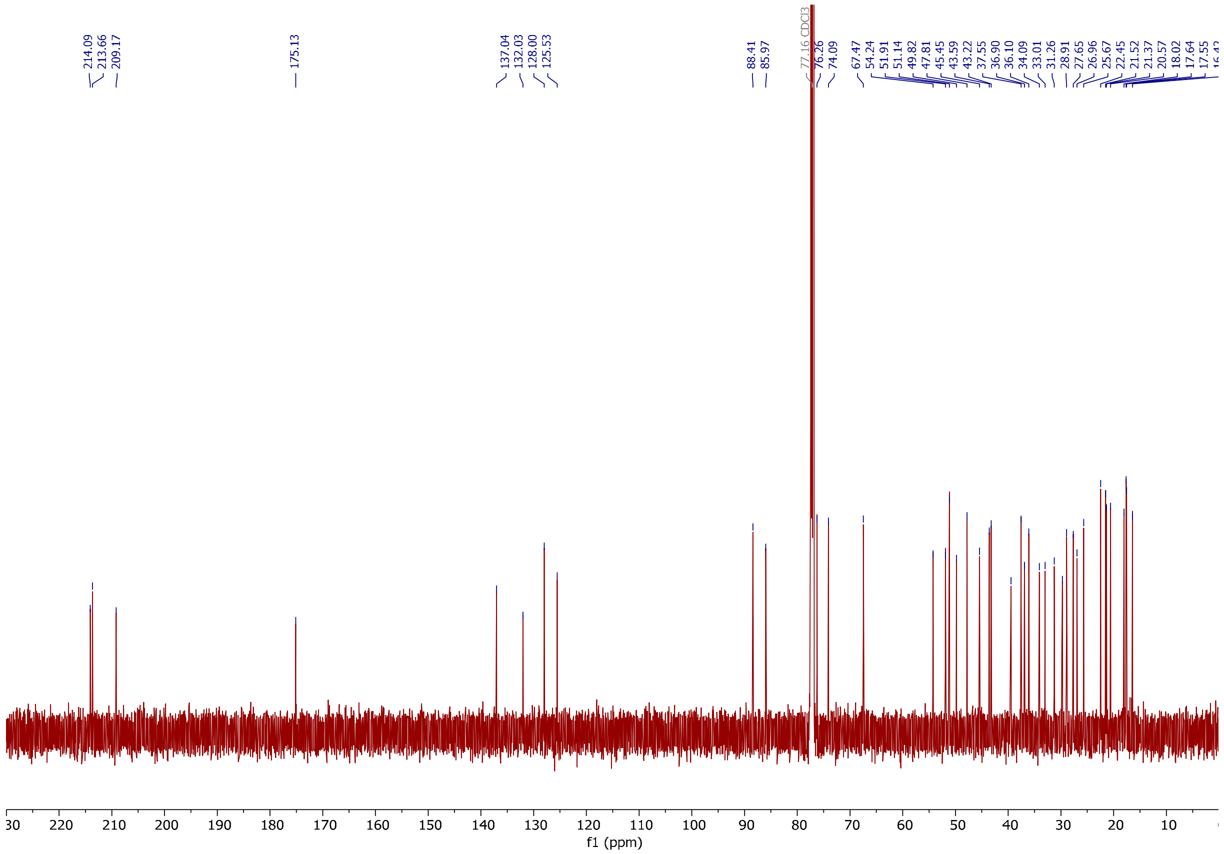

Figure S29. ^{13}C NMR spectrum of Methyl tortuoate D (**4**) in $CDCl_3$ at 150 MHz

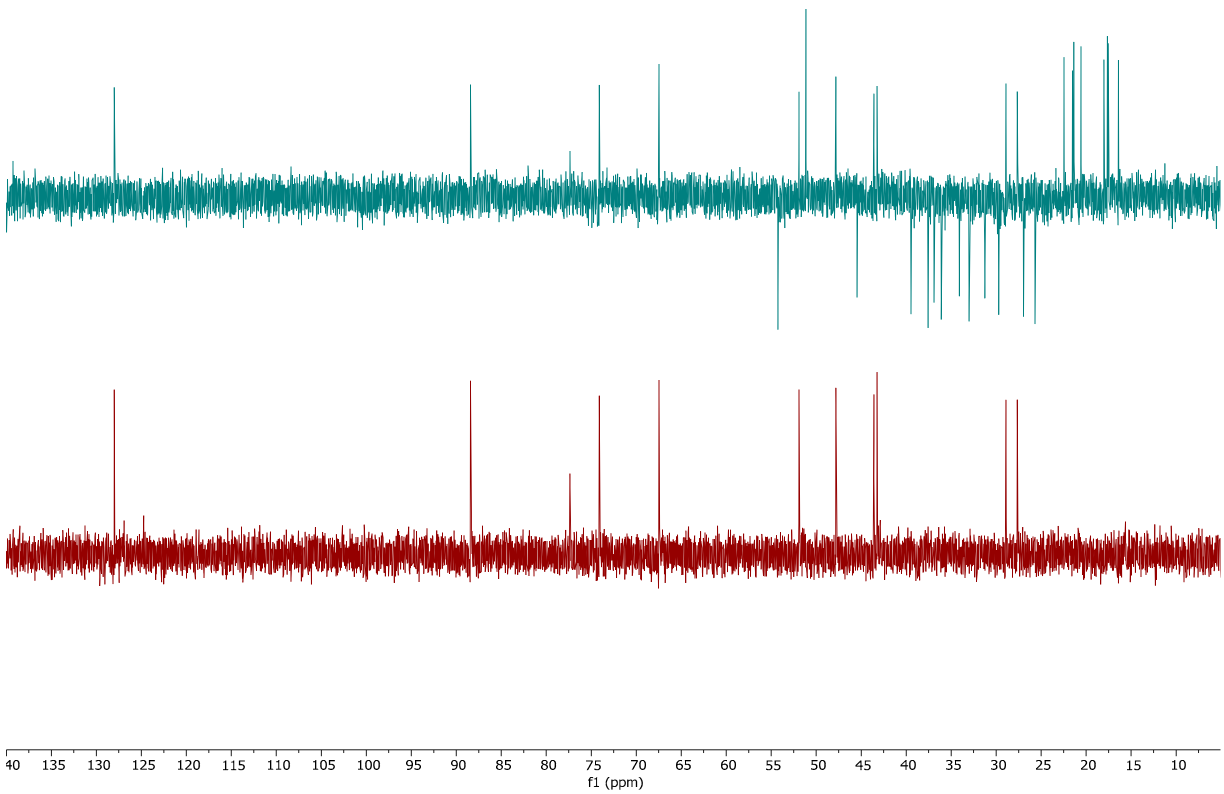

Figure S30. DEPT spectrum of Methyl tortuoate D (**4**) in $CDCl_3$ at 150 MHz

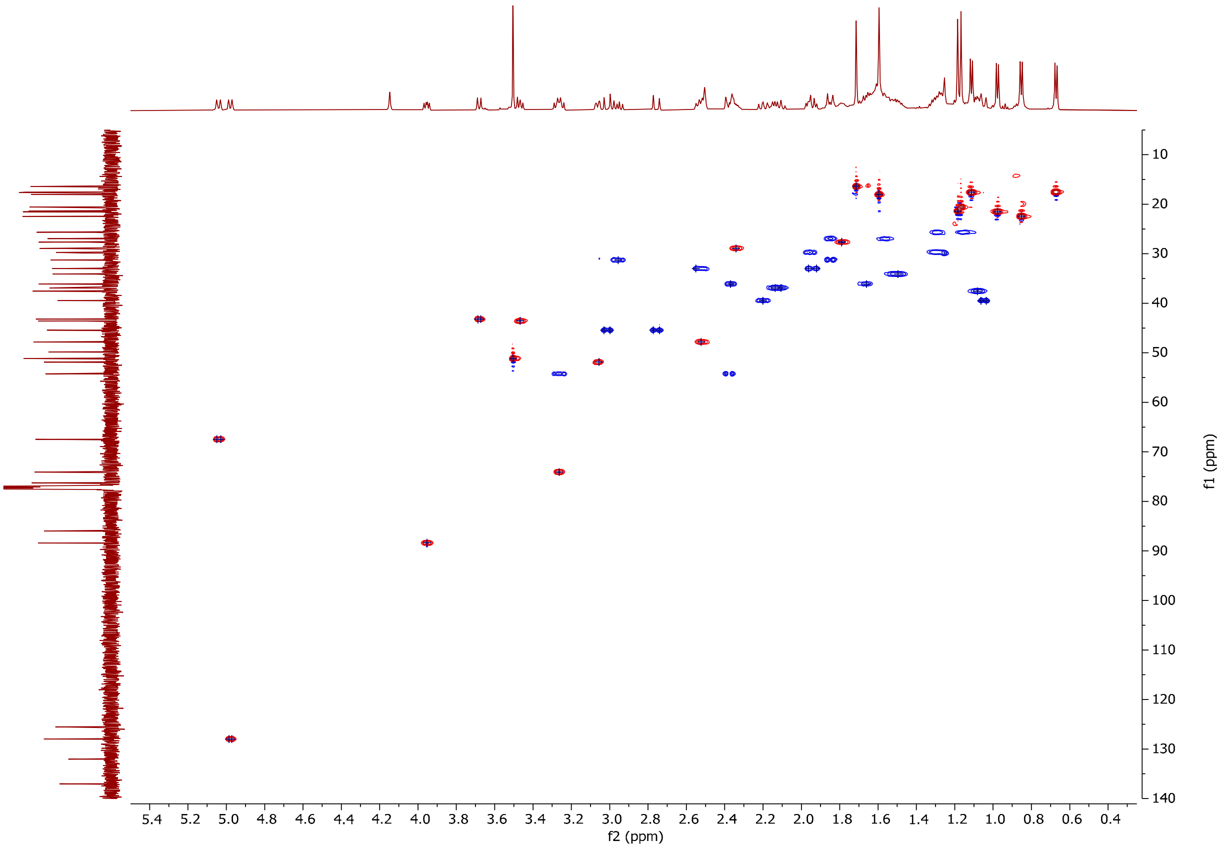

Figure S31. HSQC spectrum of Methyl tortuoate D (**4**) in $CDCl_3$

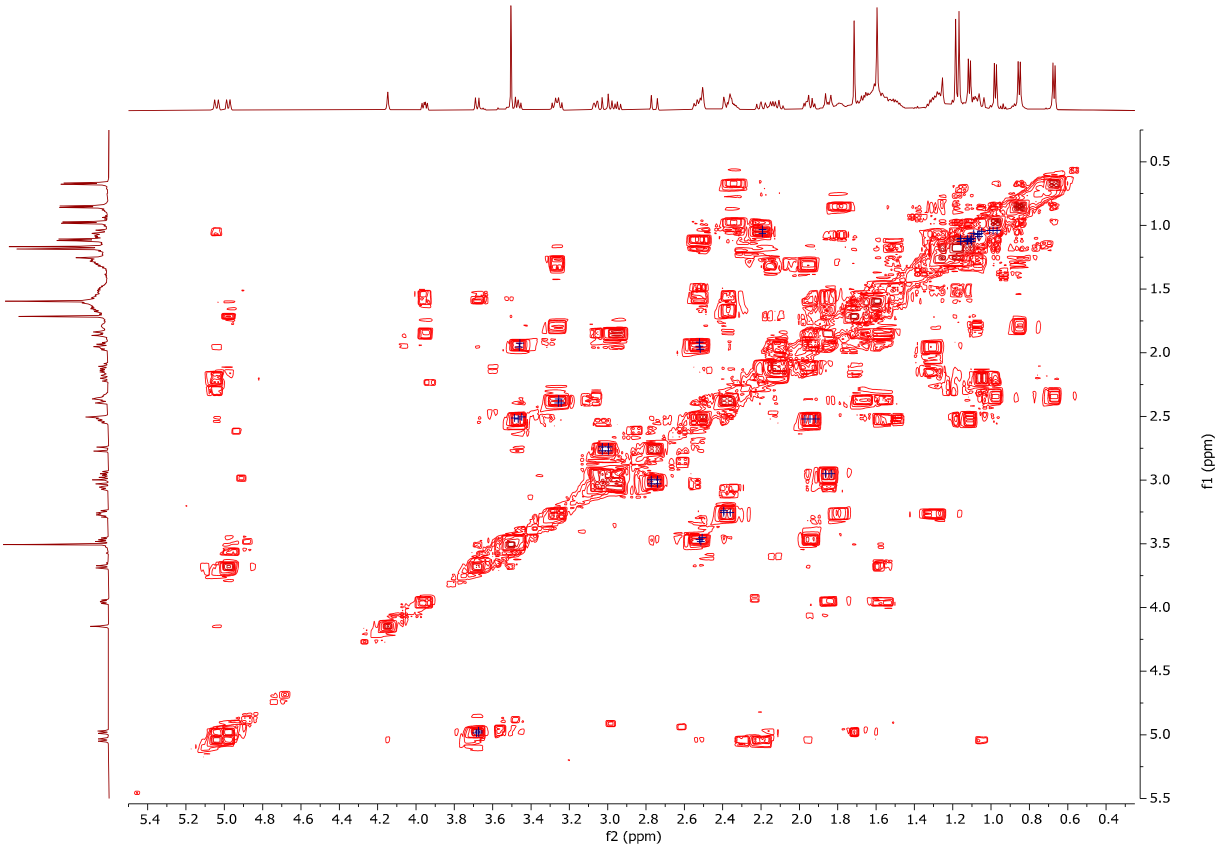

Figure S32. COSY spectrum of Methyl tortuoate D (**4**) in $CDCl_3$

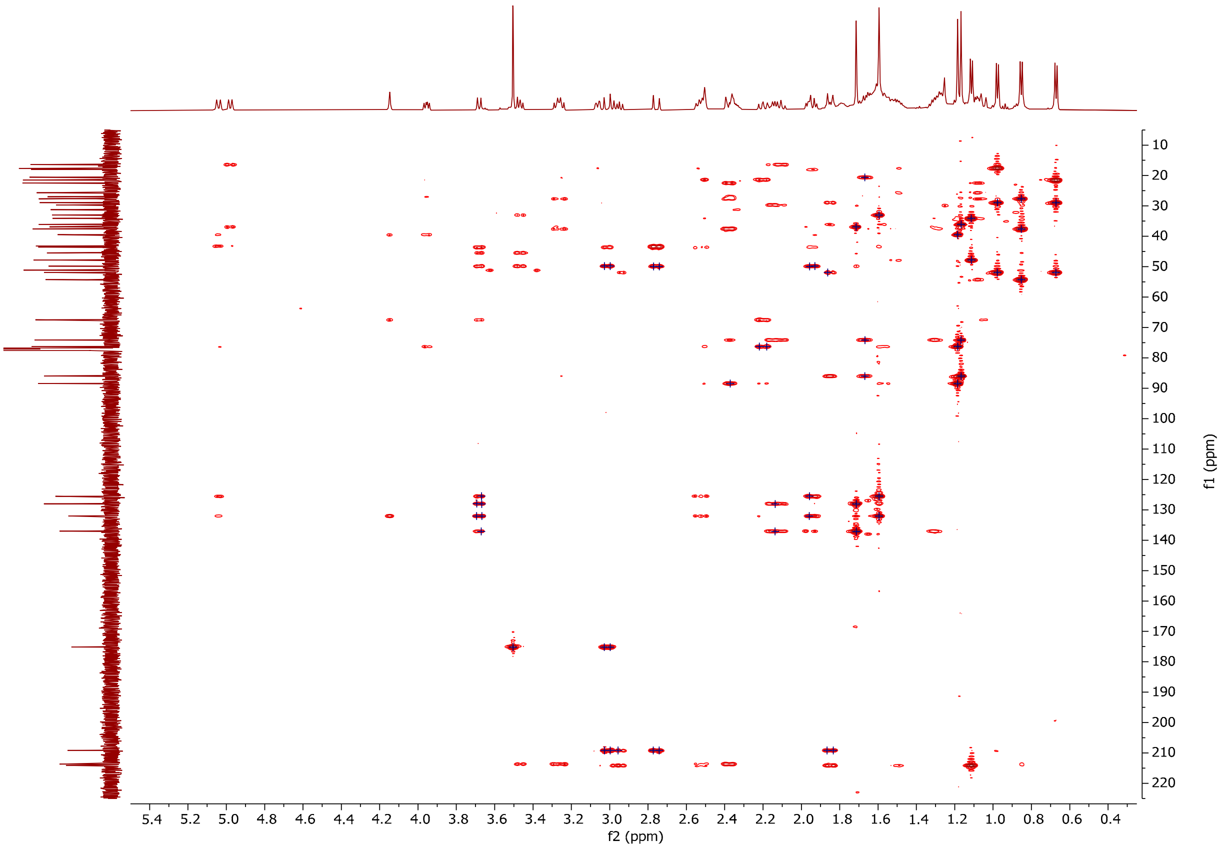

Figure S33. HMBC spectrum of Methyl tortuoate D (**4**) in $CDCl_3$

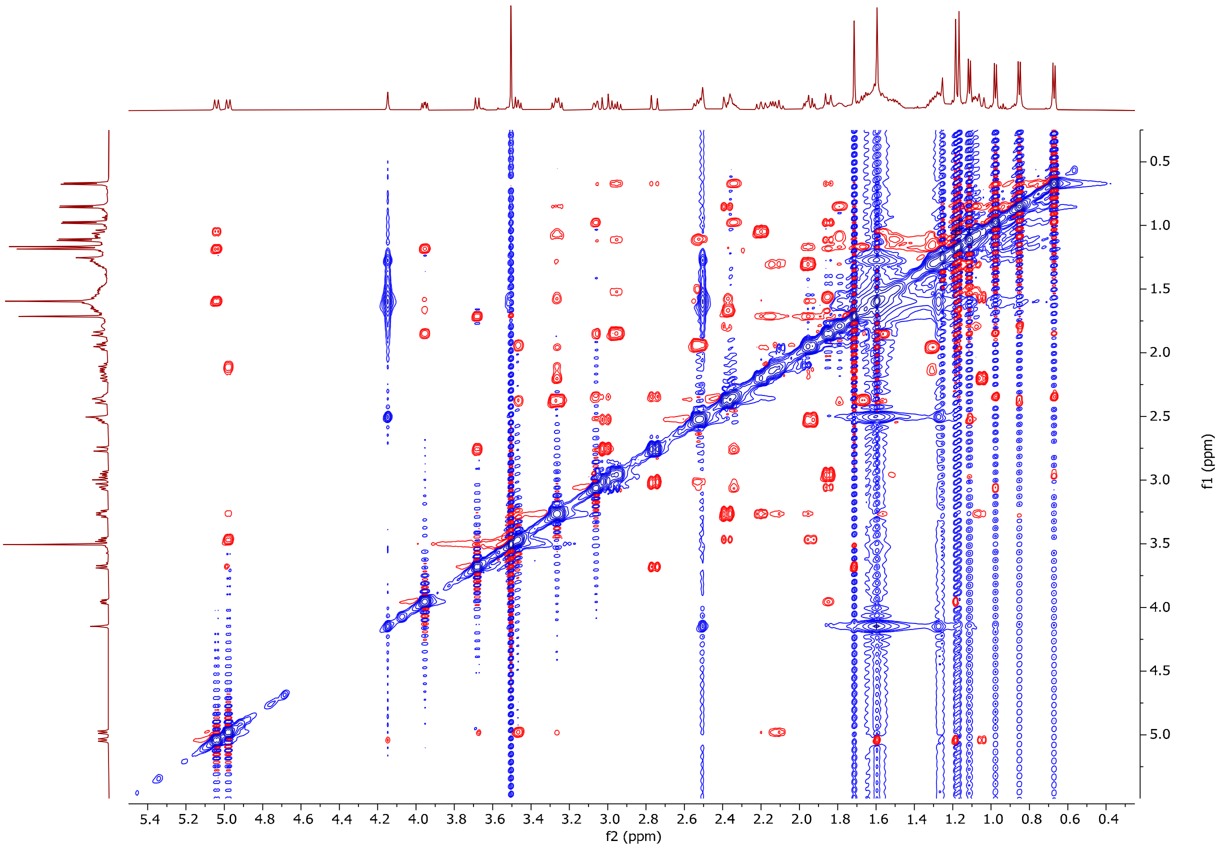

Figure S34. NOESY spectrum of Methyl tortuoate D (**4**) in $CDCl_3$

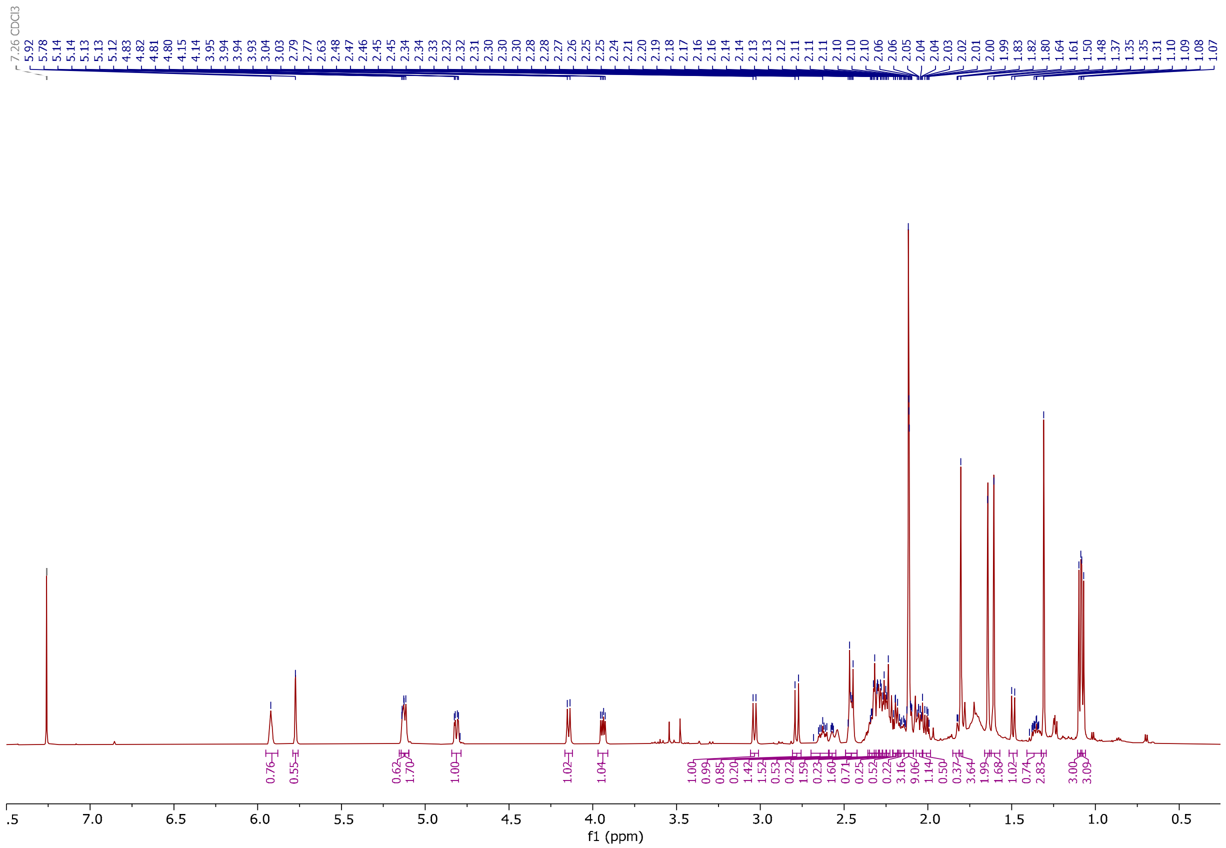

Figure S35. ^{1}H NMR spectrum of Glaucumolide A (**5**) in $CDCl_3$ at 600 MHz

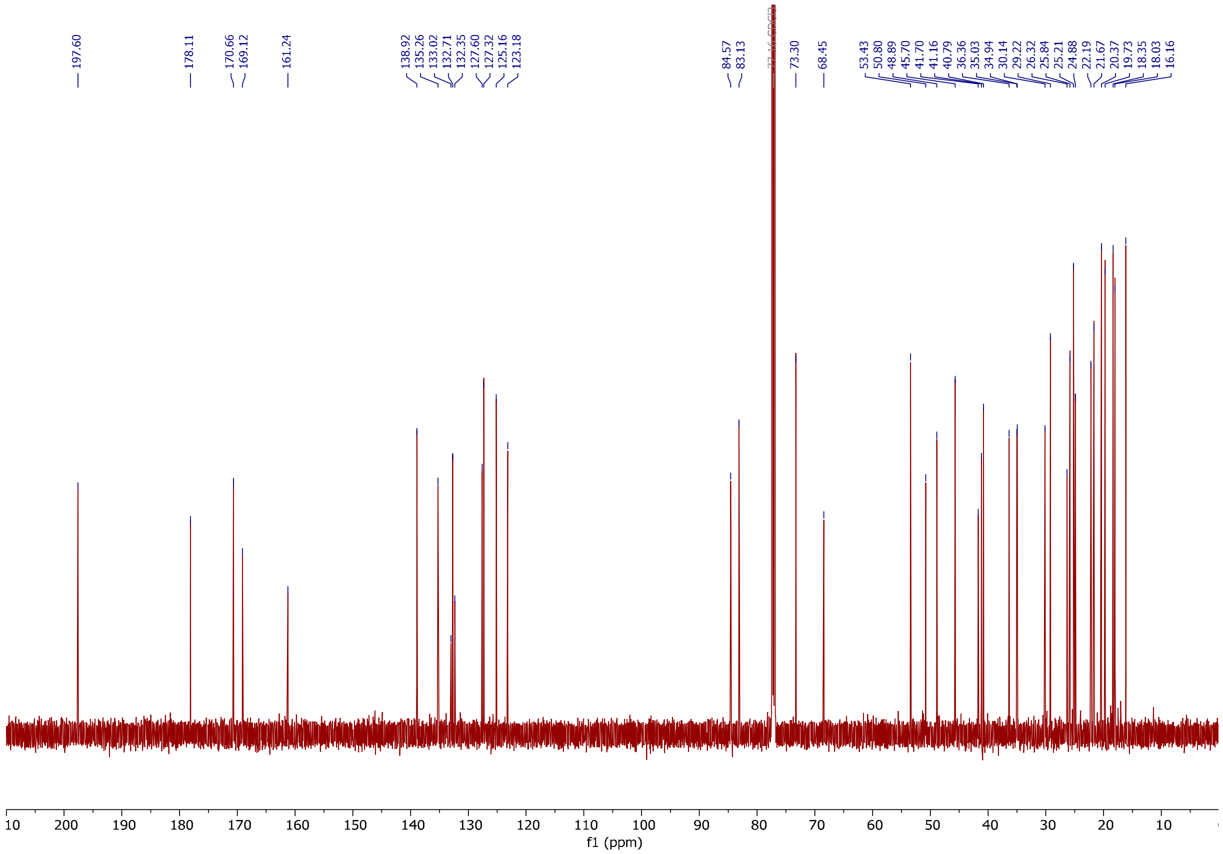

Figure S36. ^{13}C NMR spectrum of Glaucumolide A (**5**) in $CDCl_3$ at 150 MHz

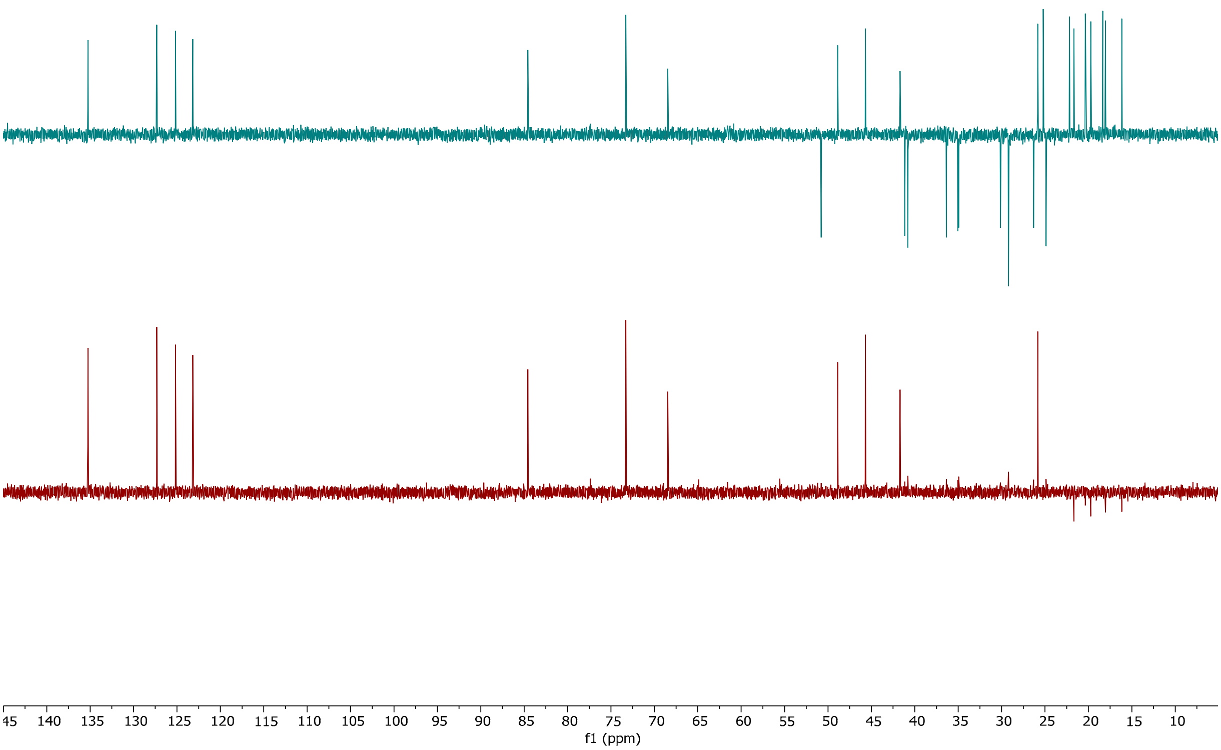

Figure S37. DEPT spectrum of Glaucumolide A (**5**) in $CDCl_3$ at 150 MHz

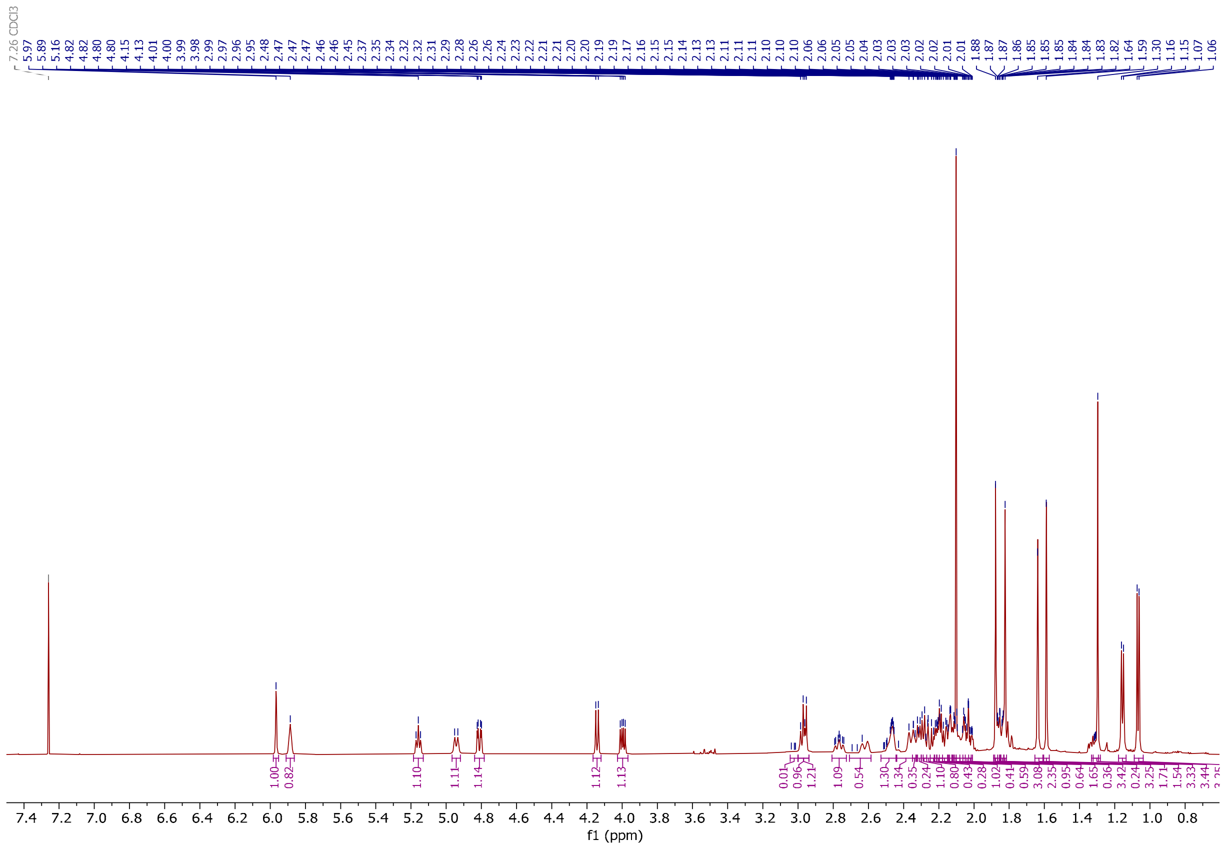

Figure S38. ^{1}H NMR spectrum of Glaucumolide B (**6**) in $CDCl_3$ at 600 MHz

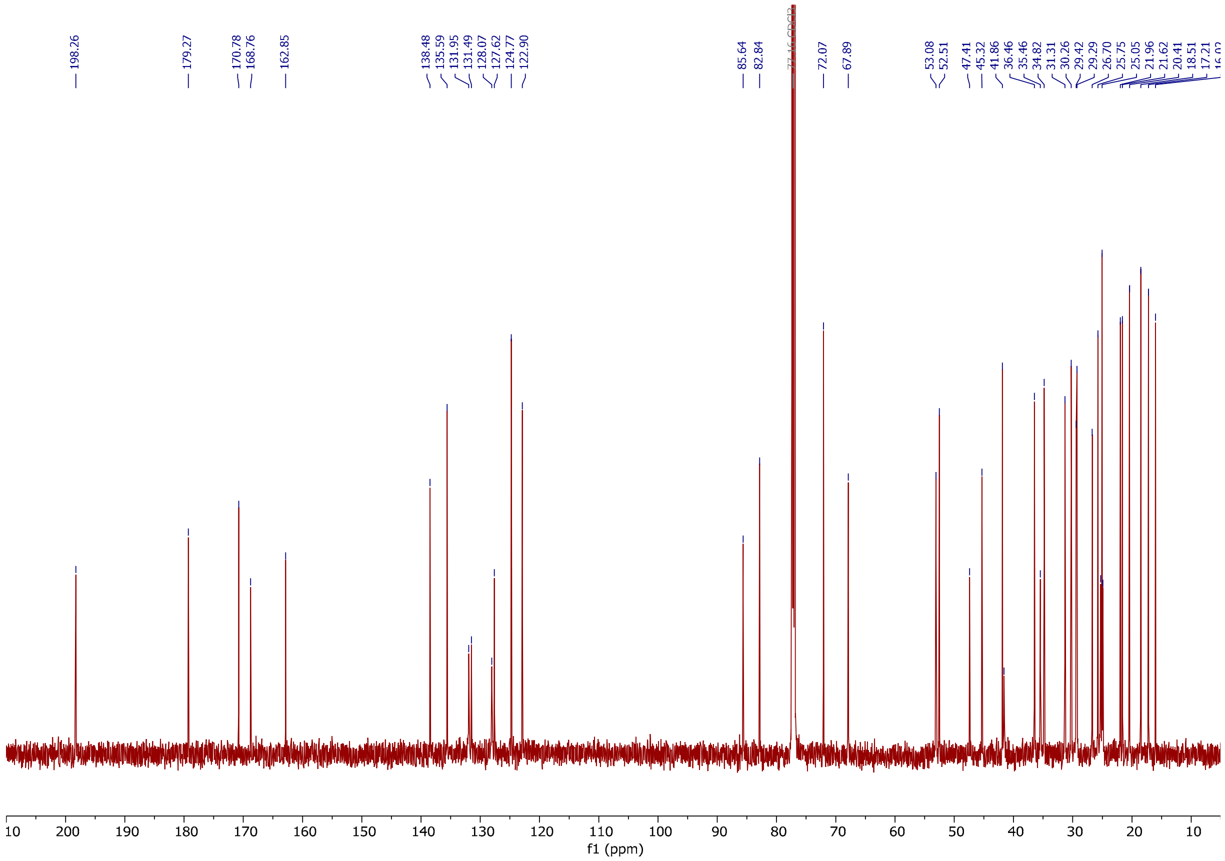

Figure S39. ^{13}C NMR spectrum of Glaucumolide B (**6**) in $CDCl_3$ at 150 MHz

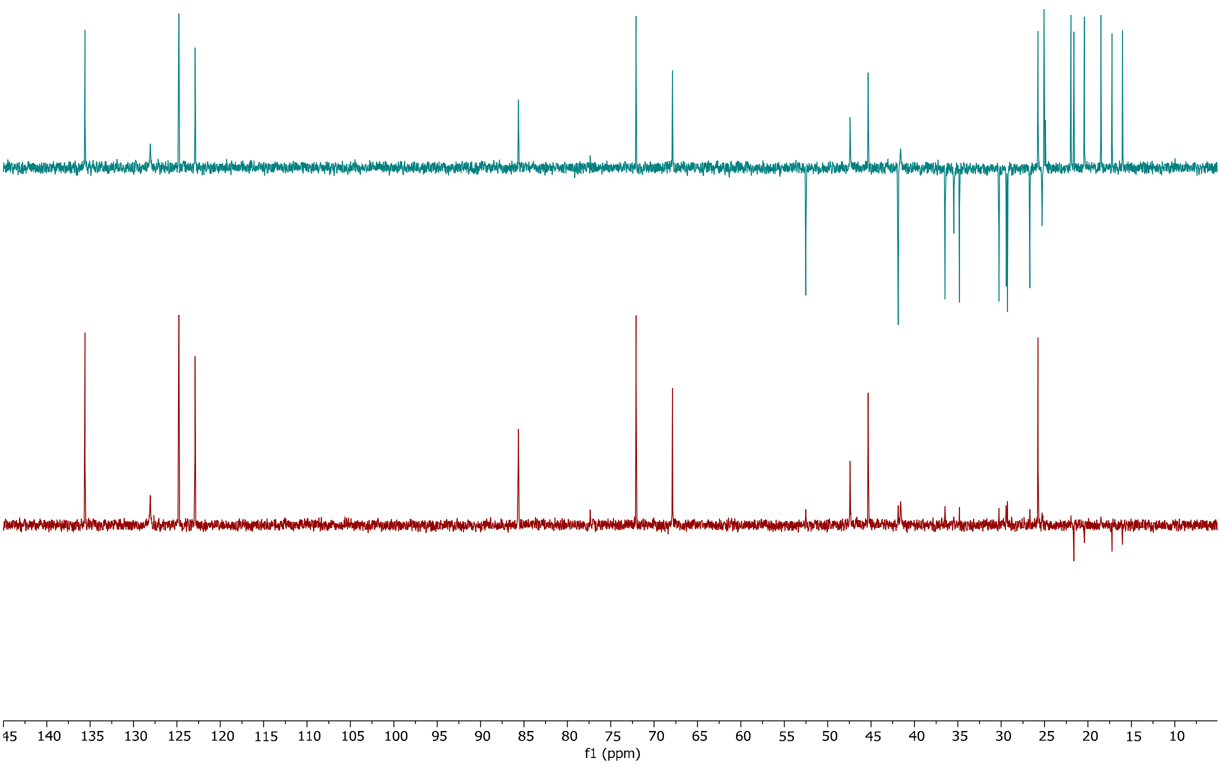

Figure S40. DEPT spectrum of Glaucumolide B (**6**) in $CDCl_3$ at 150 MHz

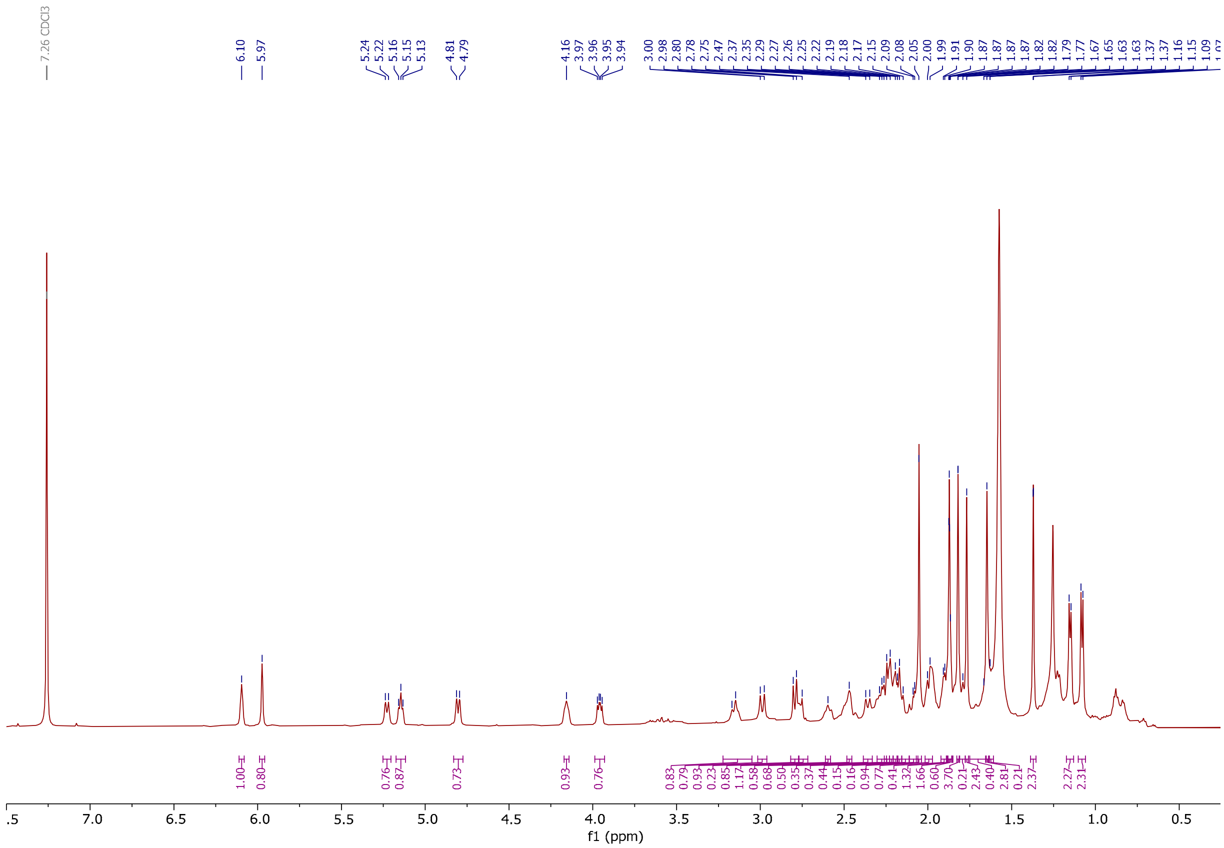

Figure S41. ^{1}H NMR spectrum of Bistrochelide A (**7**) in $CDCl_3$ at 600 MHz

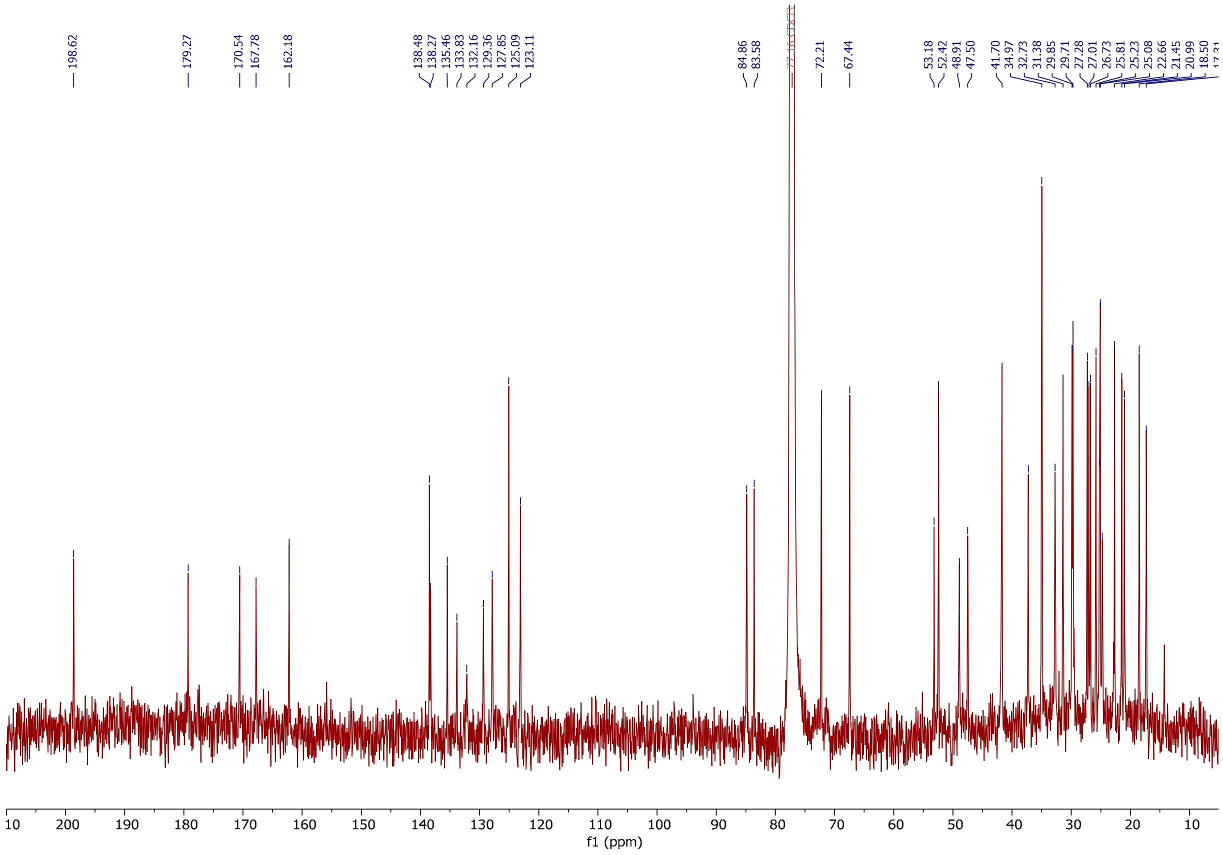

Figure S42. ^{13}C NMR spectrum of Bistrochelide A (**7**) in $CDCl_3$ at 150 MHz

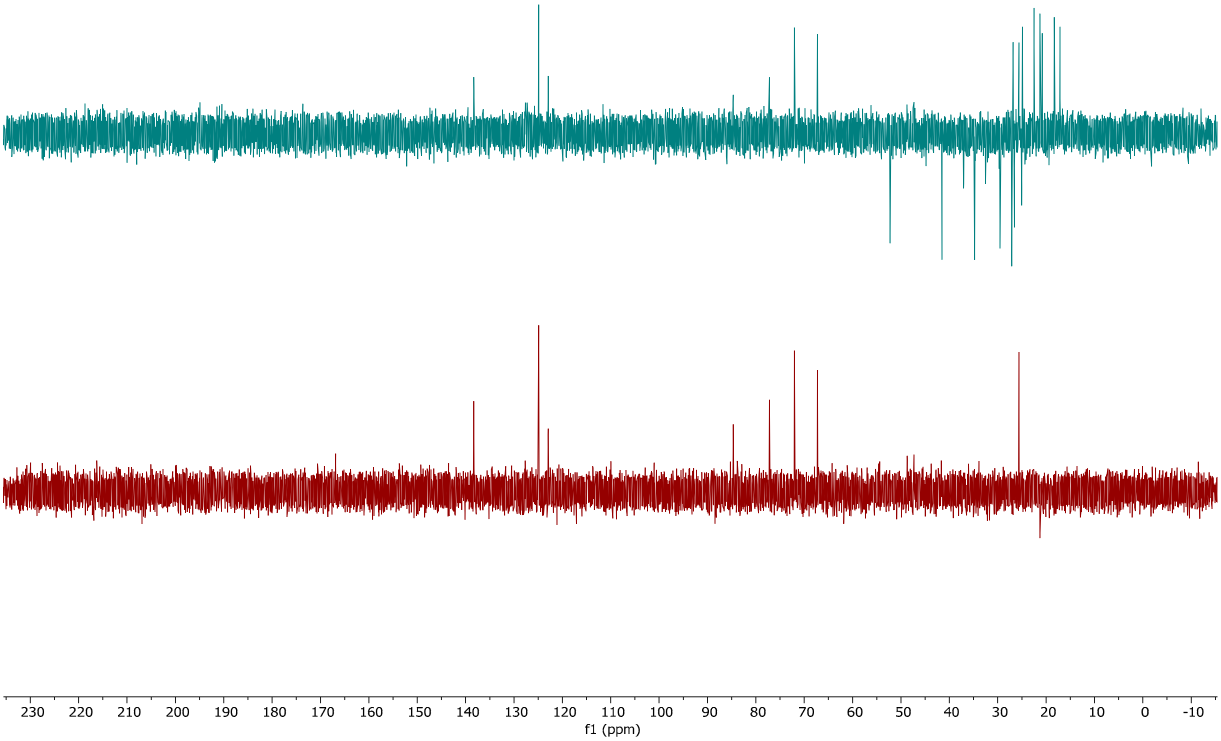

Figure S43. DEPT spectrum of Bistrochelide A (7) in $CDCl_3$ at 150 MHz

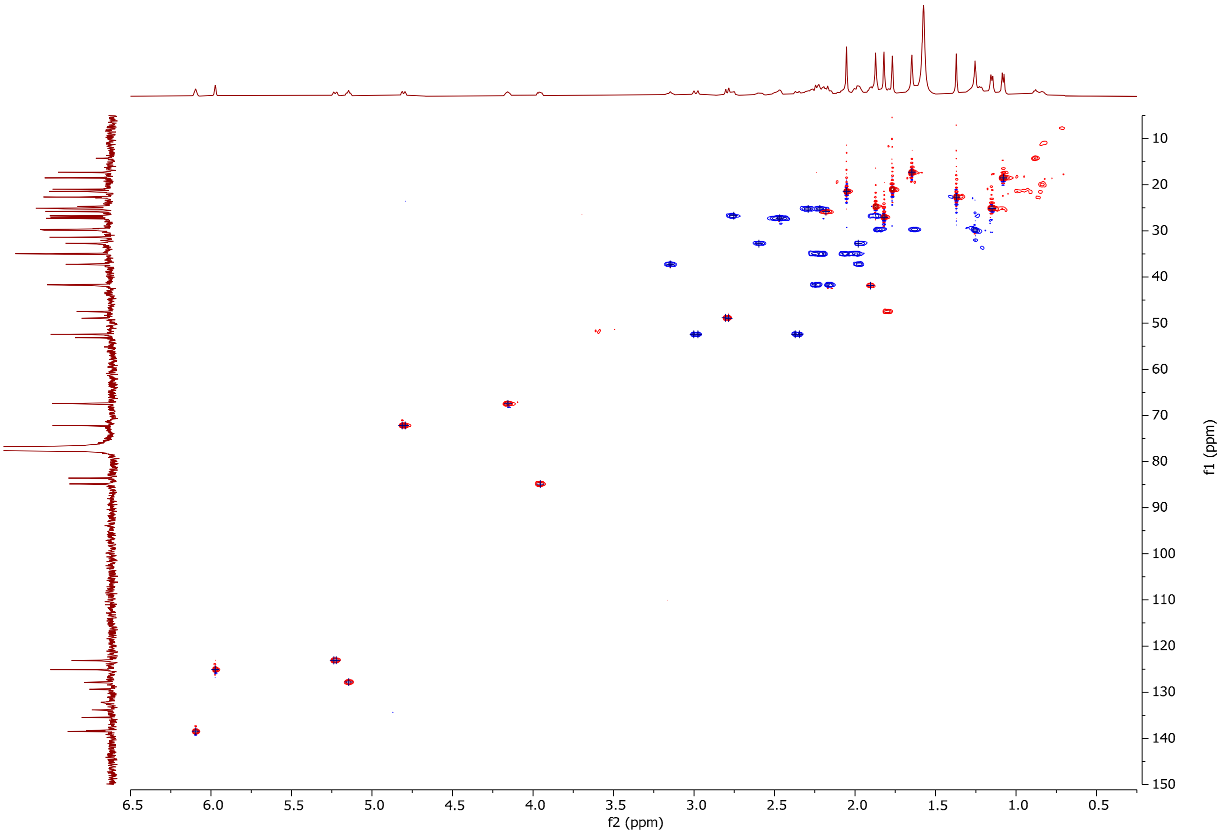

Figure S44. HSQC spectrum of Bistrochelide A (7) in $CDCl_3$

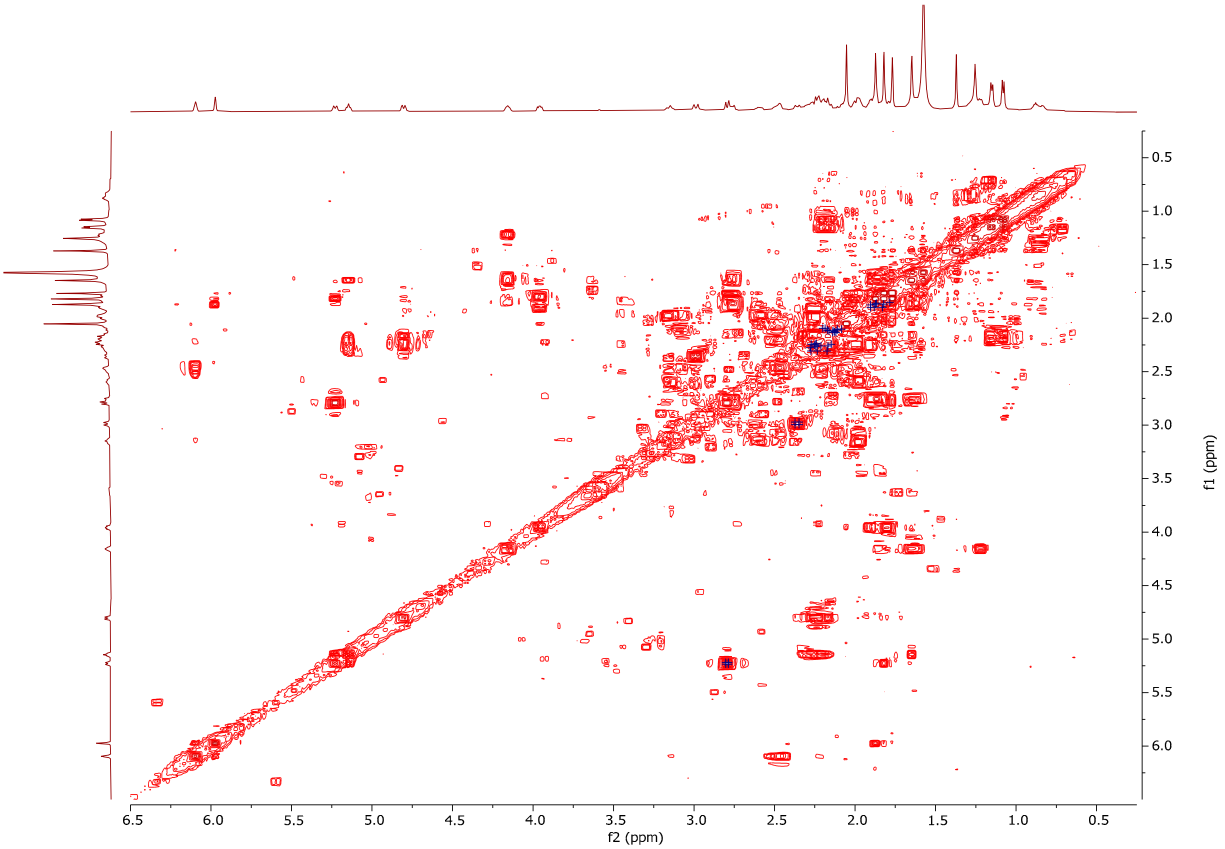

Figure S45. COSY spectrum of Bistrochelide A (7) in $CDCl_3$

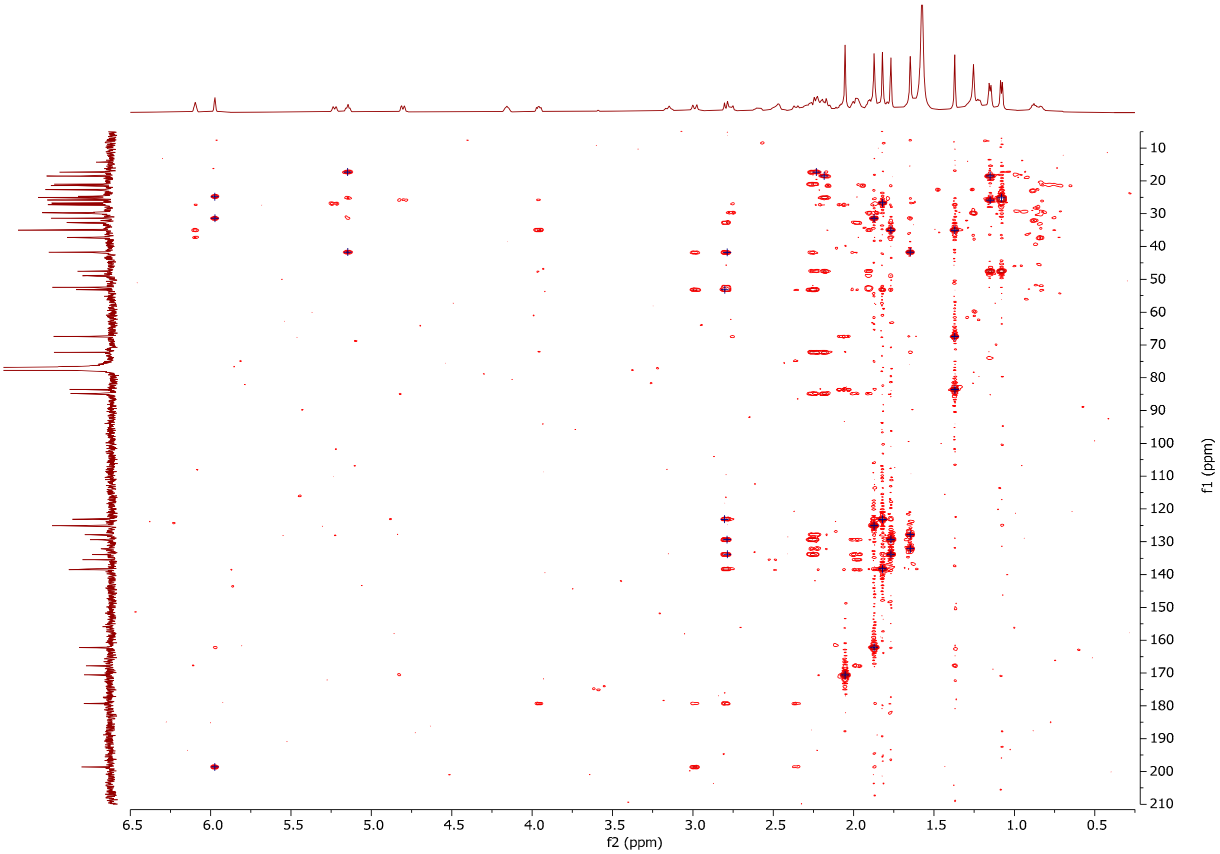

Figure S46. HMBC spectrum of Bistrochelide A (**7**) in $CDCl_3$

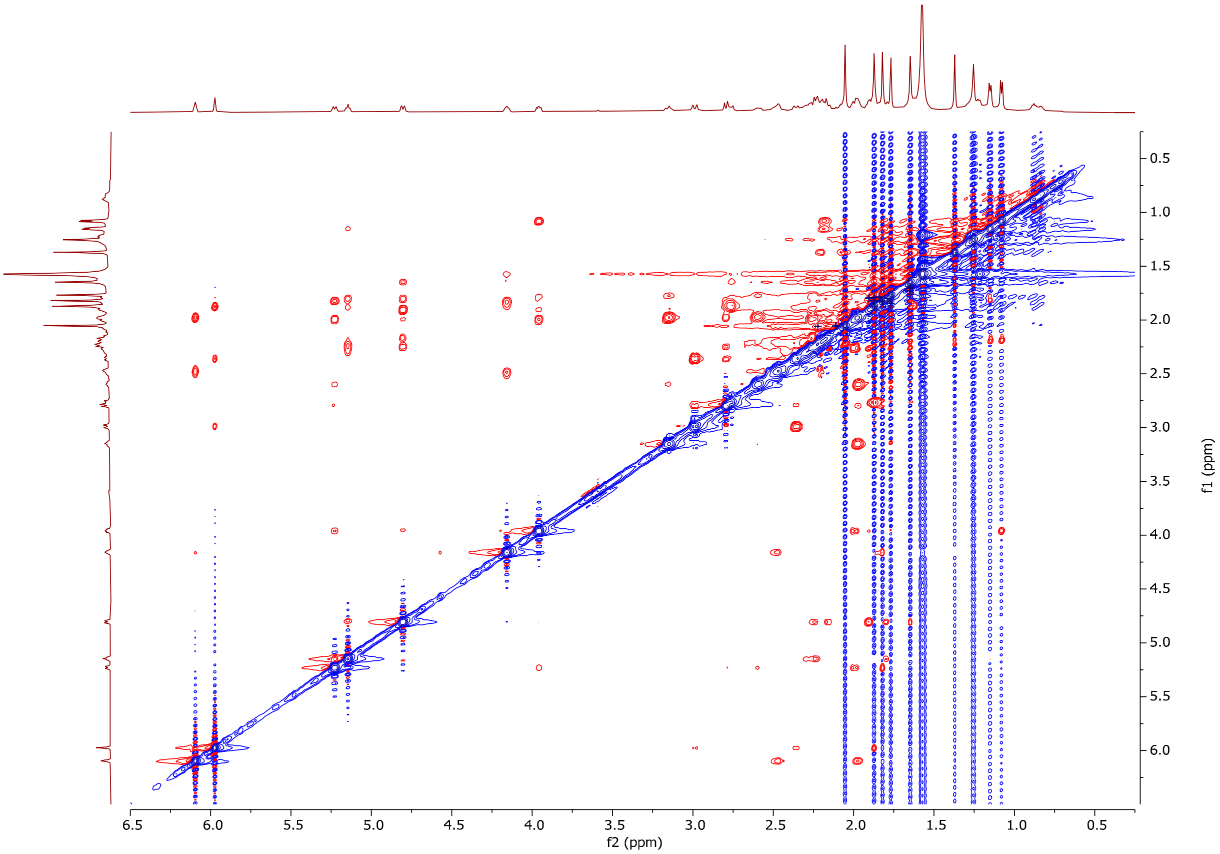

Figure S47. NOESY spectrum of Bistrochelide A (**7**) in $CDCl_3$

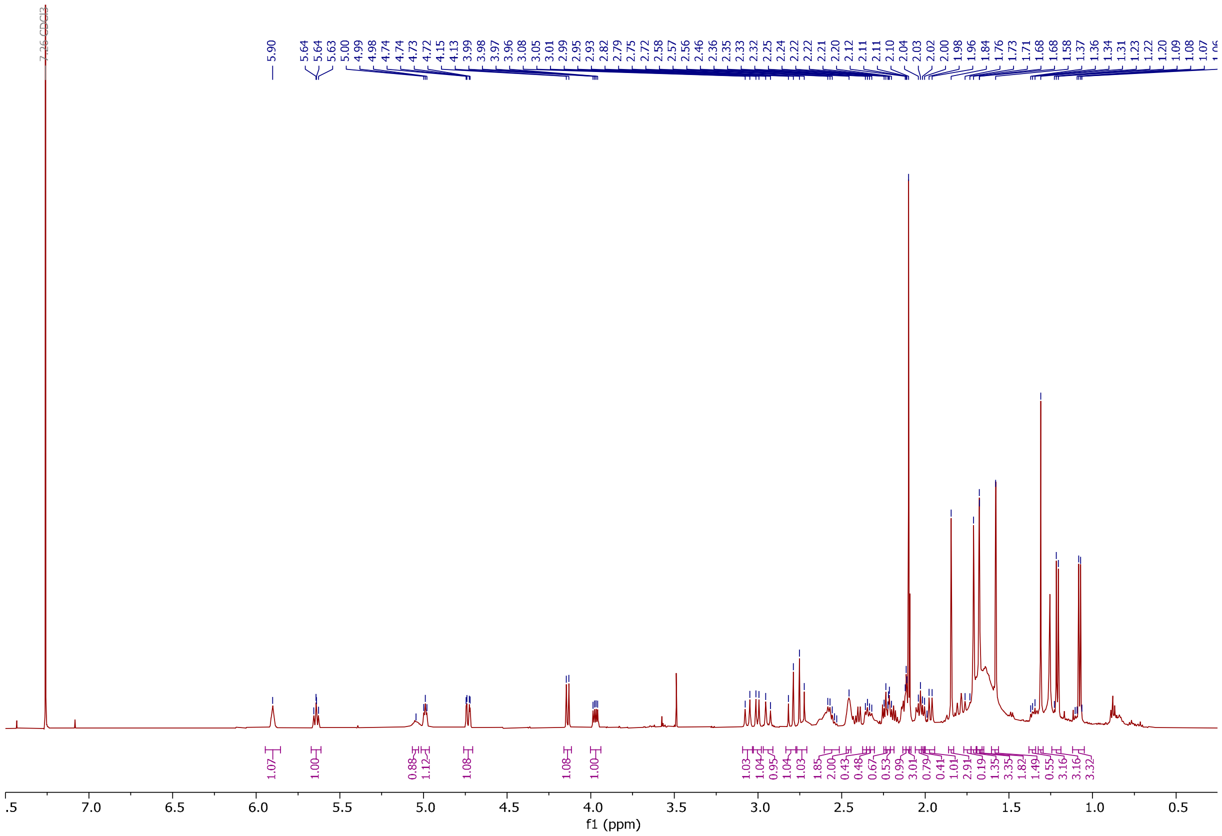

Figure S48. ^{1}H NMR spectrum of Bistrochelide B (**8**) in $CDCl_3$ at 600 MHz

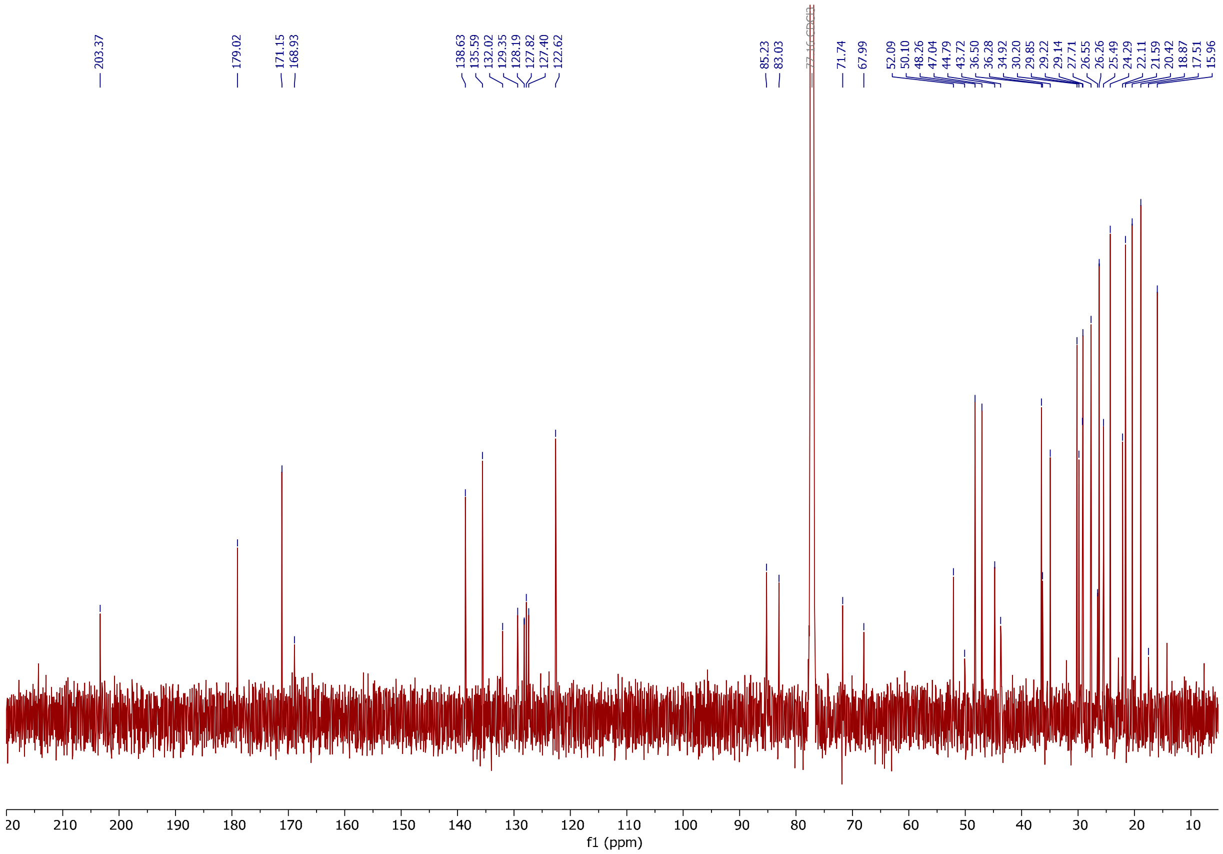

Figure S49. ^{13}C NMR spectrum of Bistrochelide B (**8**) in $CDCl_3$ at 150 MHz

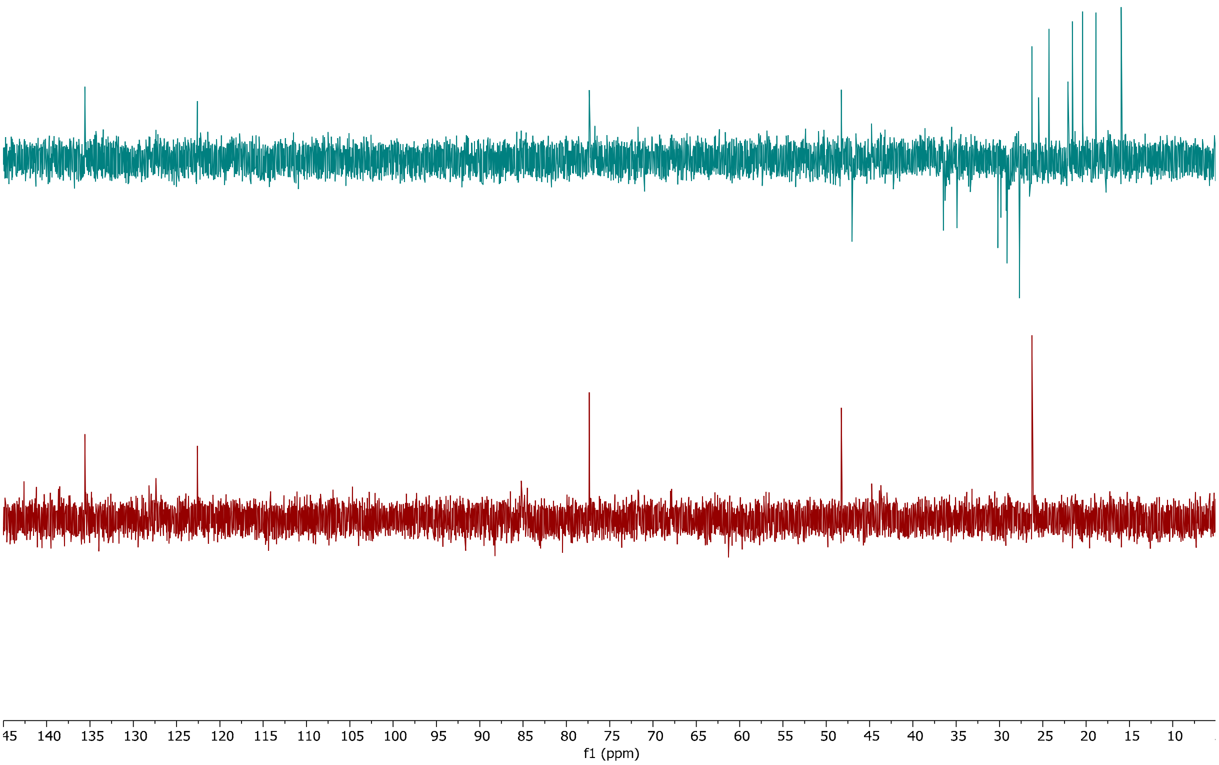

Figure S50. DEPT spectrum of Bistrochelide B (**8**) in $CDCl_3$ at 150 MHz

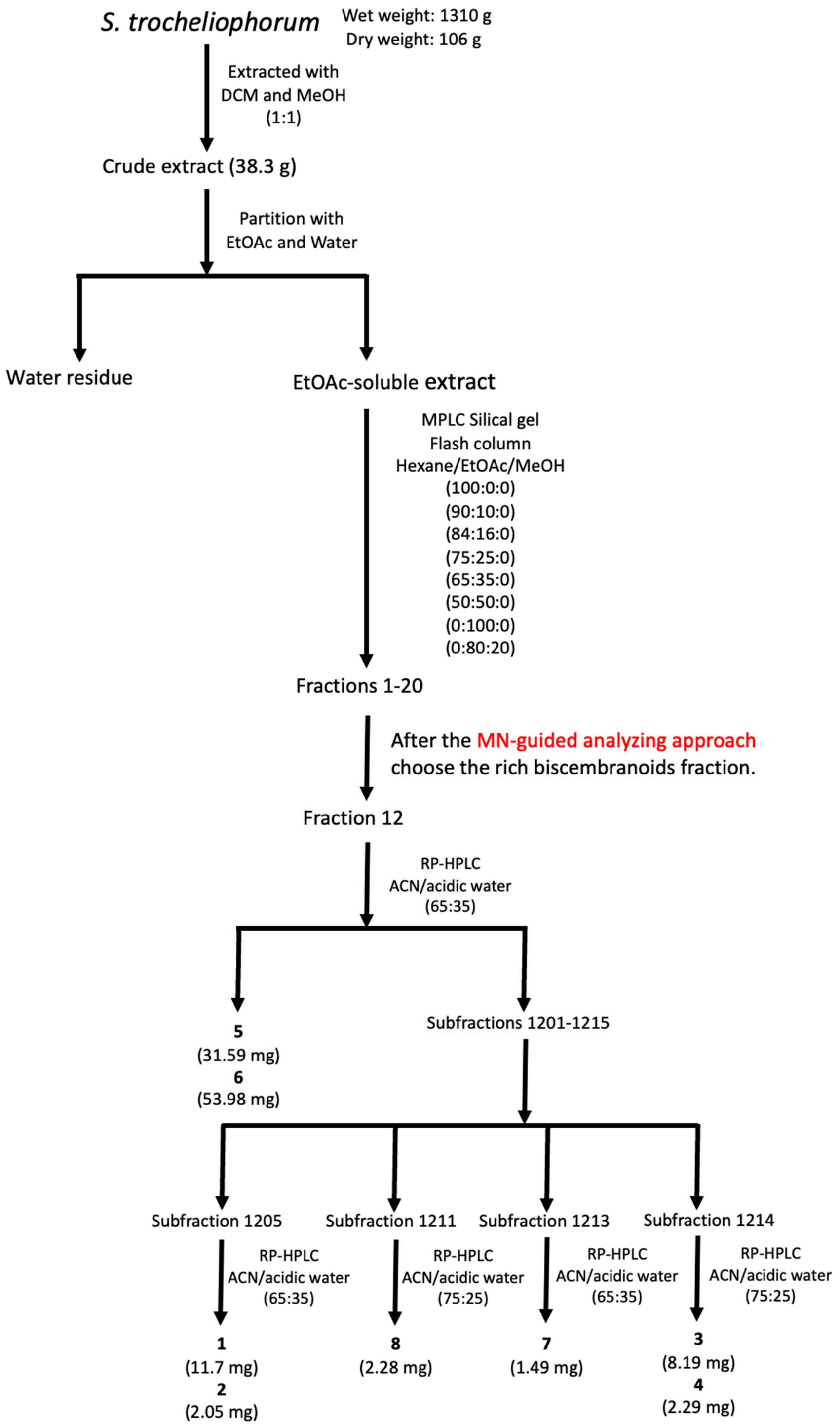

Figure S51. The process of isolation from soft coral *Sarcophyton trocheliophorum*

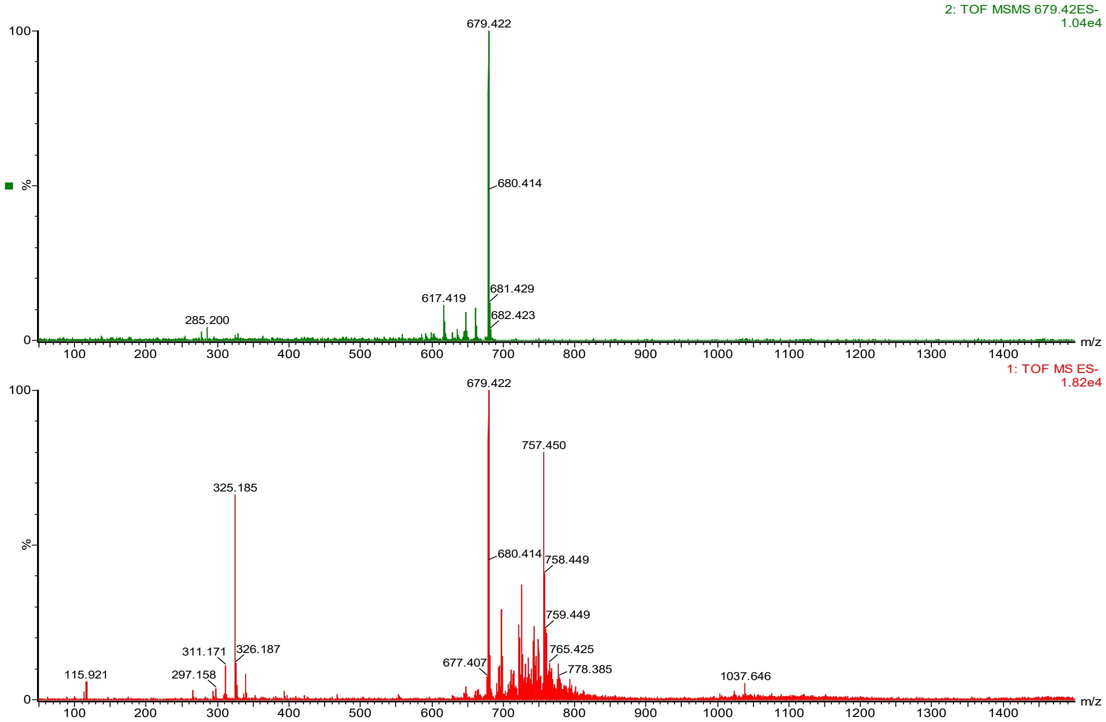

Figure S52. The LC-MS/MS fragment in negative mode of (**1**)

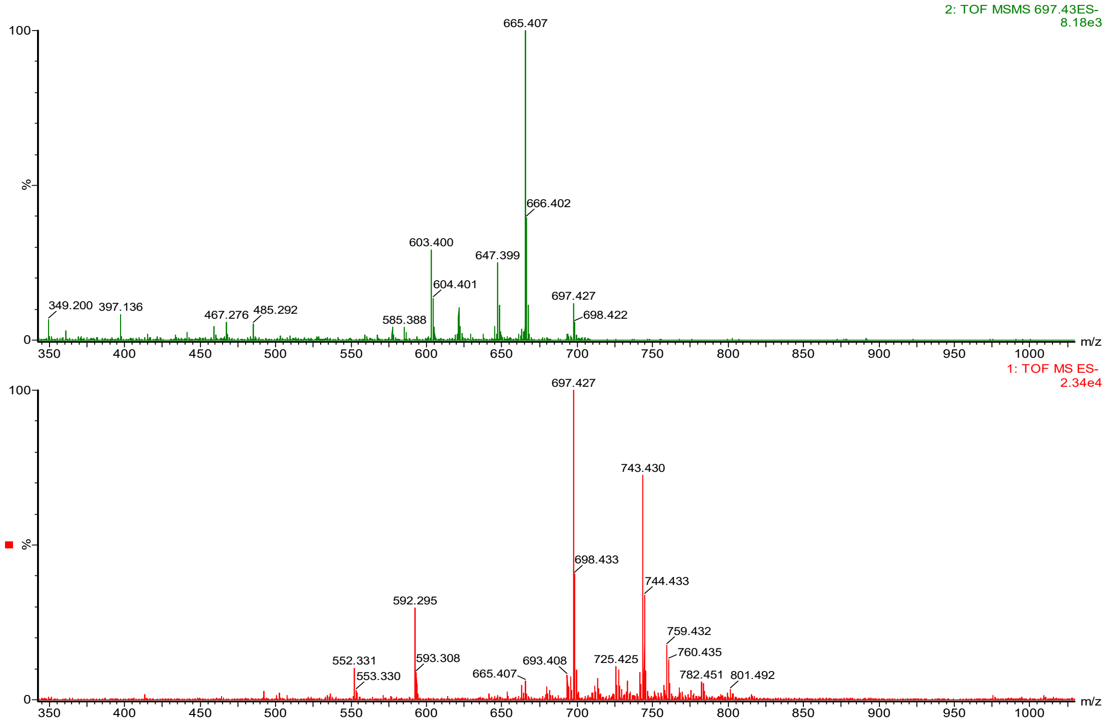

Figure S53. The LC-MS/MS fragment in negative mode of (**2**)
